# The Working Alliance Inventory's Measurement Properties: A Systematic Review

**DOI:** 10.3389/fpsyg.2022.945294

**Published:** 2022-07-15

**Authors:** Davy Paap, Yasmaine H. J. M. Karel, Arianne P. Verhagen, Pieter U. Dijkstra, Jan H. B. Geertzen, Grieteke Pool

**Affiliations:** ^1^Department of Rehabilitation Medicine, University Medical Center Groningen, University of Groningen, Groningen, Netherlands; ^2^Department of Rheumatology and Clinical Immunology, University Medical Center Groningen, University of Groningen, Groningen, Netherlands; ^3^Department of Physical Therapy, Saxion University of Applied Science, Enschede, Netherlands; ^4^Center of Expertise Caring Society 3.0, Avans University of Applied Science, Breda, Netherlands; ^5^Department General Practice, Erasmus Medical Centre University, Rotterdam, Netherlands; ^6^Discipline of Physiotherapy, Graduate School of Health, University of Technology Sydney, Sydney, NSW, Australia; ^7^Department of Oral and Maxillofacial Surgery, University Medical Center Groningen, University of Groningen, Groningen, Netherlands; ^8^Section Health Psychology, Faculty of Medical Sciences, University Medical Centre Groningen, University of Groningen, Groningen, Netherlands

**Keywords:** therapeutic alliance, systematic review, psychometric, COSMIN systematic review, Working Alliance Inventory, measurement properties, systematic literature review

## Abstract

Measurement properties of the Working Alliance Inventory (WAI) and its various translations and adaptations for specific target groups have been investigated for over 30 years. No systematic review analyzing studies on measurement properties of the WAI has been conducted to date. COnsensus-based Standards for the selection of health Measurements INstruments (COSMIN) were developed for conducting high-quality systematic reviews on measurement properties in a transparent and standardized way. Aim of this study was to systematically review studies on measurement properties of the WAI, and its adapted versions, within psychotherapy, and other healthcare contexts using COSMIN criteria. PsycINFO, Medline, and EMBASE were searched (1989–2021). In all phases of the review procedure, study selection, data extraction, risk of bias assessment, rating of the quality of measurement properties, and rating of the quality of evidence for measurement properties, disagreement between reviewers was resolved by discussion. Results on validity, internal structure, reliability, construct validity, and responsiveness were analyzed. In total 66 studies were included. In most studies, evidence for measurement properties was according to COSMIN criteria, insufficient, lacking, or conflicting. Content validity was rated insufficient because neither patients nor healthcare professionals were involved in the development and validation process. Hence evidence for content validity of the WAI is unknown. Conflicting evidence was found for structural validity. Evidence for internal consistency could not be established. Limited evidence was found for inter-rater reliability and convergent validity. Conflicting evidence was also found for test-retest reliability and divergent validity. COSMIN criteria exposed persistent problems in validation studies of the WAI. These findings may indicate that measurement properties of the WAI are not up to current standards, or that COSMIN criteria may be less appropriate for assessing measurement properties of the WAI, or it could indicate both. The results of this systematic review suggest that WAI outcomes should be interpreted with caution and further research is needed regarding the content validity and hypotheses development. For the future, the theoretical framework underlying the measurement of the working alliance needs to be studied in psychotherapy and other health contexts, and tested in methodologically sound studies.

**Systematic Review Registration:**

https://www.crd.york.ac.uk/PROSPERO/, identifier CRD42019051428.

## Introduction

The quality of the (therapeutic) working alliance has been regarded as an important general predictor of treatment outcomes in professional helping alliances, regardless of the specific context or intervention used (Horvath et al., 2011; Del Re et al., 2012). According to Bordin (1979), a working alliance between client/patient and professional helper comprises, irrespective of the (healthcare) context, three factors: agreement on goals, agreement on tasks, and development and quality of the therapeutic bond. Bordin's conceptualization of the construct working alliance originates from psychotherapeutic theory, in which this alliance was presumed to be an important vehicle for influencing treatment effects and was seen as a catalyst for change. In his original article, Bordin suggests that the aforementioned three factors may be generalizable to all types of disciplines and treatment relationships (Bordin, 1979) because a working alliance between a client/patient seeking change and a change agent (professional helper) occurs also in those healthcare contexts.

In the past three decades, research on the influence of the working alliance on treatment outcomes has increasingly been conducted in psychotherapy but also in other fields, including medicine, rehabilitation, physiotherapy, education, nursing, social work, psychology, and forensic science (Flückiger et al., 2018; Horvath, 2018). The biopsychosocial paradigm that underpins the role of psychological and social factors in the treatment of clients/patients with both somatic and psychological or social problems has become more dominant in medical healthcare. As a result, the idea of the working alliance also gained momentum in medical healthcare. The biopsychosocial paradigm implies the need for person-centered care and collaborative attunement between helper and client/patient (Wade and Halligan, 2017; Holopainen et al., 2020).

Within psychotherapy, several meta-analyses have shown positive associations between the reported quality of the working alliance and treatment outcomes [effect sizes (ES) ≈ 0.26], across a broad spectrum of treatments and domains (Martin et al., 2000; Horvath et al., 2011; Flückiger et al., 2018; Del Re et al., 2021). Also in other fields positive associations between the quality of the working alliance and treatment outcomes were found, with effect sizes (ES) ranging from 0.19 to 0.32 (Norcross and Lambert, 2014; Babatunde et al., 2017). However, the presumed role of the working alliance has been questioned in these findings (Crits-Christoph et al., 2020). Not only can alliance ratings be influenced by confounders such as satisfaction with the treatment, symptom change, and other contextual factors (DeRubeis et al., 2006; Webb et al., 2012; Falkenström et al., 2013), also the construct of the working alliance remains psychometrically unclear (Horvath and Greenberg, 1989; Elvins and Green, 2008; Hall et al., 2010; Horvath, 2018).

The original 36-item WAI (Horvath and Greenberg, 1989) was based on Bordin's theory (Bordin, 1979), and the items were theoretically formulated on the basis of the three aforementioned factors of the working alliance. The WAI-S (WAI-short form, 12 items) followed shortly thereafter, using confirmatory factor analysis (Tracey and Kokotovic, 1989). Replication research resulted in the WAI-SR (WAI-short revised form, 12 items) (Hatcher and Gillaspy, 2006). Compared with the WAI and the WAI-S, the WAI-SR demonstrated a better representation of the three alliance factors and an improved fit in confirmatory factor analysis, in part because negatively worded items were excluded (Hatcher and Gillaspy, 2006). The WAI has also been adapted for other target populations than psychotherapy patients. However, Horvath (2018) noted that on the one hand the growing number of working alliance measures may reflect dissatisfaction with existing measures on the nature and impact of the helping relationship. On the other hand, it may also reflect confusion about the concept of the working alliance, due to its fluid and unbounded nature. So, besides the WAI, also other alliance measures may suffer from methodological problems.

Concerning the WAI, until now it has remained unclear which factor structure fits the best and whether the presumed theoretical structure can sufficiently be confirmed across different studies. Some studies confirmed a three-factor structure (e.g., Horvath and Greenberg, 1989; Tracey and Kokotovic, 1989; Busseri and Tyler, 2003; Munder et al., 2009), while others confirmed a two-factor structure that combined task and goal factors (e.g., Andrusyna et al., 2001).

Measurement properties of the WAI and its various adaptations for specific target groups have been studied for over 30 years (Horvath and Greenberg, 1989). However, outcomes of these measurement properties studies are quite diverse. To the best of our knowledge, no systematic review analyzing studies on measurement properties of the WAI has been conducted to date. A systematic review of existing measurement studies may identify the methodological qualities of these studies and provide an up-to-date overview of the actual measurement properties of the WAI. Such a review can help with the interpretation of WAI outcomes and with assessing feasibility of applying the WAI in clinical practice. Additionally, it may offer suggestions for future research on measurement properties.

In the past decade, COnsensus-based Standards for the selection of health Measurements INstruments (COSMIN) were developed (Terwee et al., 2012; Mokkink et al., 2016). The aim was to offer researchers tools for conducting high-quality systematic reviews on measurement properties of Patient-Reported Outcome Measures (PROMs), in a transparent and standardized way (Prinsen et al., 2018). The development of these standards was based on international Delphi studies, which aimed to reach consensus on definitions and assessments of measurements properties (Mokkink et al., 2010a,b; Terwee et al., 2012; Gagnier et al., 2021). COSMIN criteria were first developed for evaluation of outcome measures in biomedical healthcare. In recent years the use of these criteria has broadened to other healthcare contexts, for instance empathy measurements in autistic and non-autistic adults (Harrison et al., 2020), anxiety in people with psychosis (Smith et al., 2021), and attachment measures in middle childhood and adolescence (Jewell et al., 2019).

This study aimed to systematically review studies that evaluate measurement properties of the WAI and its adapted versions, in the context of psychotherapy and other healthcare contexts, to obtain an up-to-date overview of the measurement qualities of the WAI and its adapted versions. After selection of eligible measurement studies using recent COSMIN criteria, this review evaluated: (1) content validity (including ceiling and floor effects); (2) internal structure (including structural validity, internal consistency, and cross-cultural validity/measurement invariance); and (3) remaining measurement properties (test-retest reliability, measurement error, criterion/construct validity, and responsiveness).

## Methods

### Design

This systematic review was conducted according to recent COSMIN guidelines (Prinsen et al., 2018) and is reported according to the Preferred Reporting Items for Systematic Reviews and Meta-Analyses (PRISMA) statement (Moher, 2009). The protocol of this review was registered in the International Prospective Register of Systematic Reviews (PROSPERO); registration number: CRD42019051428.

In all phases of the review procedure, study selection, data extraction, risk of bias assessment, rating of the quality of measurement properties, and rating of the quality of evidence for measurement properties, disagreement between reviewers (DP and YK) was resolved by discussion. In case of persistent disagreement, a third reviewer (PD) participated in the discussion and a binding decision.

COSMIN manuals (Prinsen et al., 2018; Terwee et al., 2018) recommend including professionals in the review team, who have experience with the construct and the target population. Since the aim of the study was to evaluate measurements properties of the WAI in several settings, this systematic review was conducted by a multidisciplinary research team. It included a psychotherapist/clinical psychologist, physiotherapists, a clinical health scientist, a rehabilitation specialist, and epidemiologists. All team members have expertise in research into the working alliance and most members have expertise in conducting systematic reviews.

### Data Sources and Searches

The databases PsycINFO, Medline, and EMBASE were searched for relevant studies. The search strategy used blocks of search terms related to the following aspects: (1) Construct of interest: Working alliance; (2) Measurement of interest: Working Alliance Inventory; and (3) Measurement properties: validity, floor and ceiling effects, factor structure, reliability, responsiveness, and interpretability (Supplementary Material 1).

The search was conducted from 1989, the publication year of the development and content validity study by Horvath & Greenberg, up to May 2021. The search strategy for this review was developed in collaboration with a medical information specialist from the University Medical Center Groningen, the Netherlands. The PubMed search filter described in Supplementary Material 1 was used for finding relevant studies. It has a sensitivity of 97.4% (Terwee et al., 2009). Reference lists of the included studies were screened for studies that were missed in the database search.

No language or type of population restrictions were set. Included studies that were written in languages the authors could not read were translated (*n* = 2).

### Eligibility Criteria and Selection of the Studies

A study was eligible for this review when it assessed one or more measurement properties of the WAI and/ or an adapted version of the WAI (Mokkink et al., 2010a). Studies that used the WAI as an outcome measure or that used the WAI for validation of other instruments were excluded. Two independent reviewers (DP and YK) assessed titles and abstracts for eligibility, followed by full-text assessment.

### Data Extraction

Data were extracted using data extraction form of COSMIN. It included country, language, study design, number and type of response categories, mean scores of the WAI, field of profession, target population, study sample characteristics, and results of the measurement properties evaluation. Data were extracted by one reviewer (DP) and checked for accuracy by a second reviewer (YK). The data extraction process was piloted on two studies included in the review.

### Assessment of Risk of Bias

Two reviewers independently used the COSMIN taxonomy and definitions, to assess risk of bias and to evaluate the performance of measurement properties in each included study (Mokkink et al., 2010b; Prinsen et al., 2018; Terwee et al., 2018). The sequence of the measurement property evaluation was: (1) content validity, including ceiling and floor effects; (2) internal structure, including structural validity, internal consistency, and cross-cultural validity and measurement invariance; and (3) remaining measurement properties (test-retest reliability, measurement error, criterion/construct validity, and responsiveness) (Prinsen et al., 2018).

The COSMIN checklist was used to assess the methodological quality of the included studies (Mokkink et al., 2010a). For each study, each item on the checklist was scored (i.e., inadequate, doubtful, adequate, or very good). COSMIN suggests to report the lowest score (Terwee et al., 2012).

### Evaluation of Measurement Properties

Measurement properties concerning results of the WAI and all its adapted versions were rated for each study, according to COSMIN criteria for good measurement properties. Details of the criteria are shown in Table 1 (Prinsen et al., 2018). In addition, for each study the quality of each measurement property was scored on a three-point rating scale (i.e., sufficient, indeterminate, or insufficient) (Prinsen et al., 2018).

**Table 1:** COSMIN Criteria for good measurement properties according to Prinsen et al. (2018).

**Measurement property**	**Rating**	**Criteria**
Content validity (including face validity)	+	All items refer to relevant aspects of the construct to be measured AND are relevant for the target population AND are relevant for the purpose of the measurement instrument AND together comprehensively reflect the construct to be measured AND all items are comprehensible to the target population
	?	Not all information for “+” reported
	-	Criteria for “+” not met
Structural validity	+	**CTT** CFA: CFI or TLI or comparable measure > 0.95 OR RMSEA <0.06 OR SRMR < 0.08^a^ **IRT/Rasch** No violation of unidimensionality^b^: CFI or TLI or comparable measure > 0.95 OR RMSEA <0.06 OR SRMR < 0.08 *AND* no violation of local independence: residual correlation among the items after controlling for dominant factor <0.20 OR Q3's <0.37 *AND* *no violation of monotonicity: adequate looking graphs OR item scalability > 0.30* *AND* *adequate model fit* IRT: χ^2^ > 0.001 Rasch: infit and outfit mean squares ≥ 0.5 and ≤ 1.5 OR Z-standardized values >−2 and <2
	?	CTT: not all information for “+” reported IRT/Rasch: model fit not reported
	-	Criteria for “+” not met
Internal consistency	+	At least low evidence^c^ for sufficient structural validity^d^ AND Cronbach's alpha(s) ≥ 0.70 for each unidimensional scale or subscale^e^
	?	Criteria for “At least low evidence^c^ for sufficient structural validity^d^” not met
	-	At least low evidence^c^ for sufficient structural validity^d^ AND Cronbach's alpha(s) < 0.70 for each unidimensional scale or subscale^e^
Reliability	+	ICC or weighted Kappa ≥ 0.70
	?	ICC or weighted Kappa not reported
	-	ICC or weighted Kappa < 0.70
Measurement error	+	SDC or LoA <MIC^d^
	?	MIC not defined
	-	SDC or LoA > MIC^d^
Hypotheses testing for construct validity	+	The result is in accordance with the hypothesis^f^
	?	No hypothesis defined
	-	The result is not in accordance with the hypothesis^f^
Cross-Cultural validity/ measurement invariance	+	No important differences found between group factors (such as age, gender, language) in multiple group factor analysis OR no important DIF for group factors (McFadden's *R*^2^ < 0.02)
	?	No multiple group factor analysis OR DIF analysis performed
	-	Important differences between group factors OR DIF was found
Criterion validity	+	Correlation with gold standard ≥ 0.70 OR AUC ≥ 0.70
	?	Not all information for “+” reported
	-	Correlation with gold standard < 0.70 OR AUC < 0.70
Responsiveness	+	The result is in accordance with the hypothesis^f^ OR AUC ≥ 0.70
	?	No hypothesis defined
	-	The result is not in accordance with the hypothesis^f^ OR AUC < 0.70

*The COSMIN criteria for good measurement properties according to Prinsen et al. (2018) and are based on, e.g., Terwee et al. (2007) and Prinsen et al. (2018)*.

*AUC, area under the curve; CFA, confirmatory factor analysis; CFI, comparative fit index; CTT, classical test theory; DIF, differential item functioning; ICC, intraclass correlation coefficient; IRT, item response theory; LoA, limits of agreement; MIC, minimal important change; RMSEA, root mean square error of approximation; SEM, standard error of measurement; SDC, smallest detectable change; SRMR, standardized root mean residuals; TLI, Tucker–Lewis index*.

*“+” = sufficient, “–” = insufficient, “?” = indeterminate*.

a *To rate the quality of the summary score, the factor structures should be equal across the studies*.

b *Unidimensionality refers to a factor analysis per subscale, while structural validity refers to a factor analysis of a (multidimensional) patient/therapists/observer reported outcome measure*.

c *As defined by the grading the evidence according to the GRADE approach*.

d *This evidence may come from different studies*.

e *The criteria “Cronbach alpha < 0.95” was deleted, as this is relevant in the development phase of a PROM and not when evaluating an existing PROM*.

f *The results of all studies should be taken together and it should then be decided whether 75% of the results is in accordance with the hypotheses*.

For studies conducted by one of the reviewing authors (DP or YK), assessment of risk of bias and evaluation of measurement properties was checked by a third independent reviewer.

### Data Synthesis of the Included Studies

The GRADE-approach was used to determine the overall quality of evidence for the measurement properties (Prinsen et al., 2018). This approach considers the following determinants: risk of bias of the studies (methodological quality of the studies), inconsistency of results between studies (i.e., unexplained inconsistency of results across studies), directness of evidence (i.e., evidence from different types of populations), and precision of evidence (i.e., sample size of the available studies). The overall quality of evidence was rated as strong, moderate, limited, conflicting, or unknown. When information regarding a measurement property was unclear or insufficient, the scoring of the methodological quality was downgraded from strong to moderate or limited or to conflicting, in case of inconsistency in results, or indirect results, or to unknown, in case of lack of reporting; therefore insufficient for rating the evidence.

## Results

**Figure 1:**
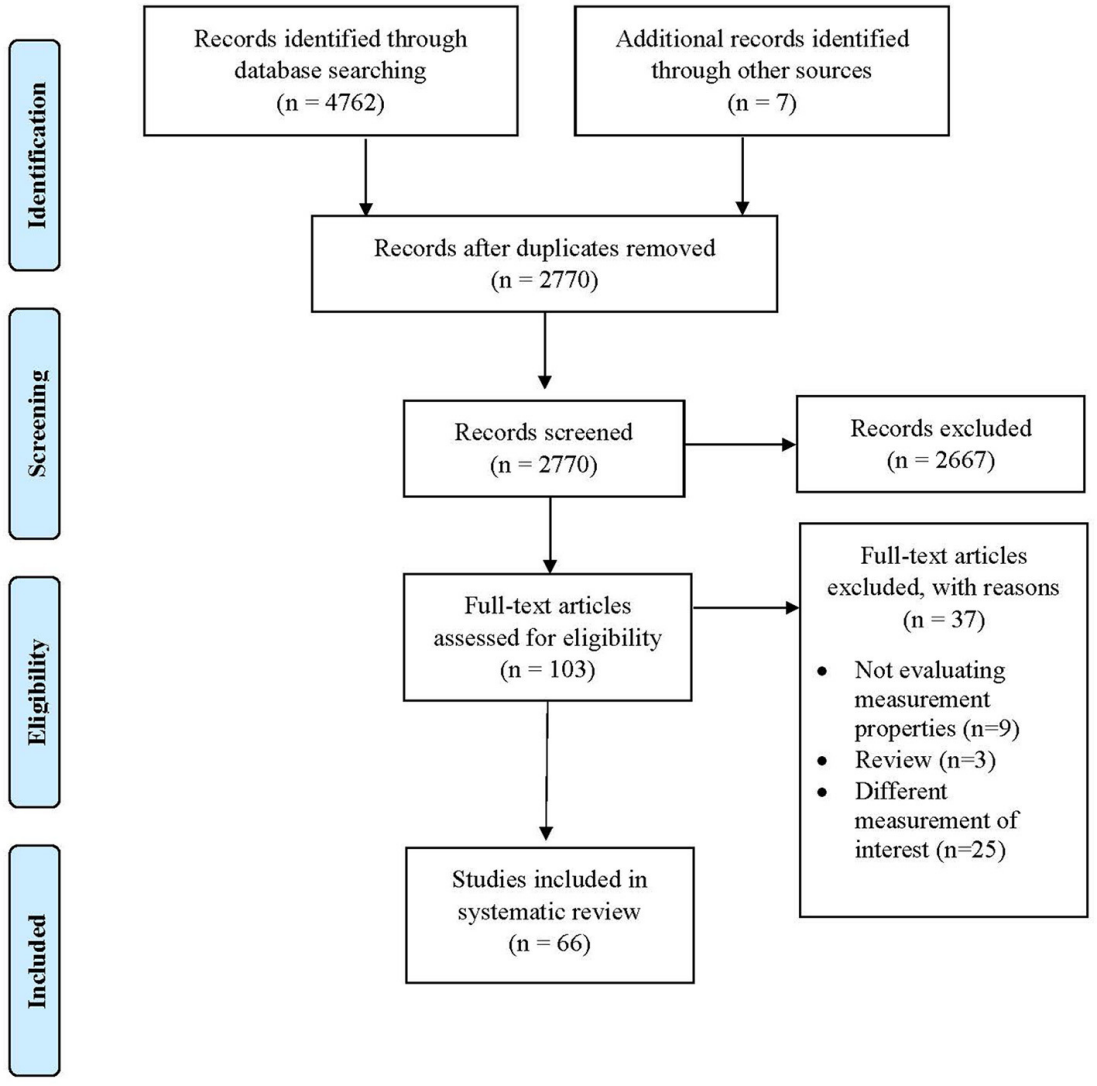
Prisma flow diagram.

**Figure 2:**
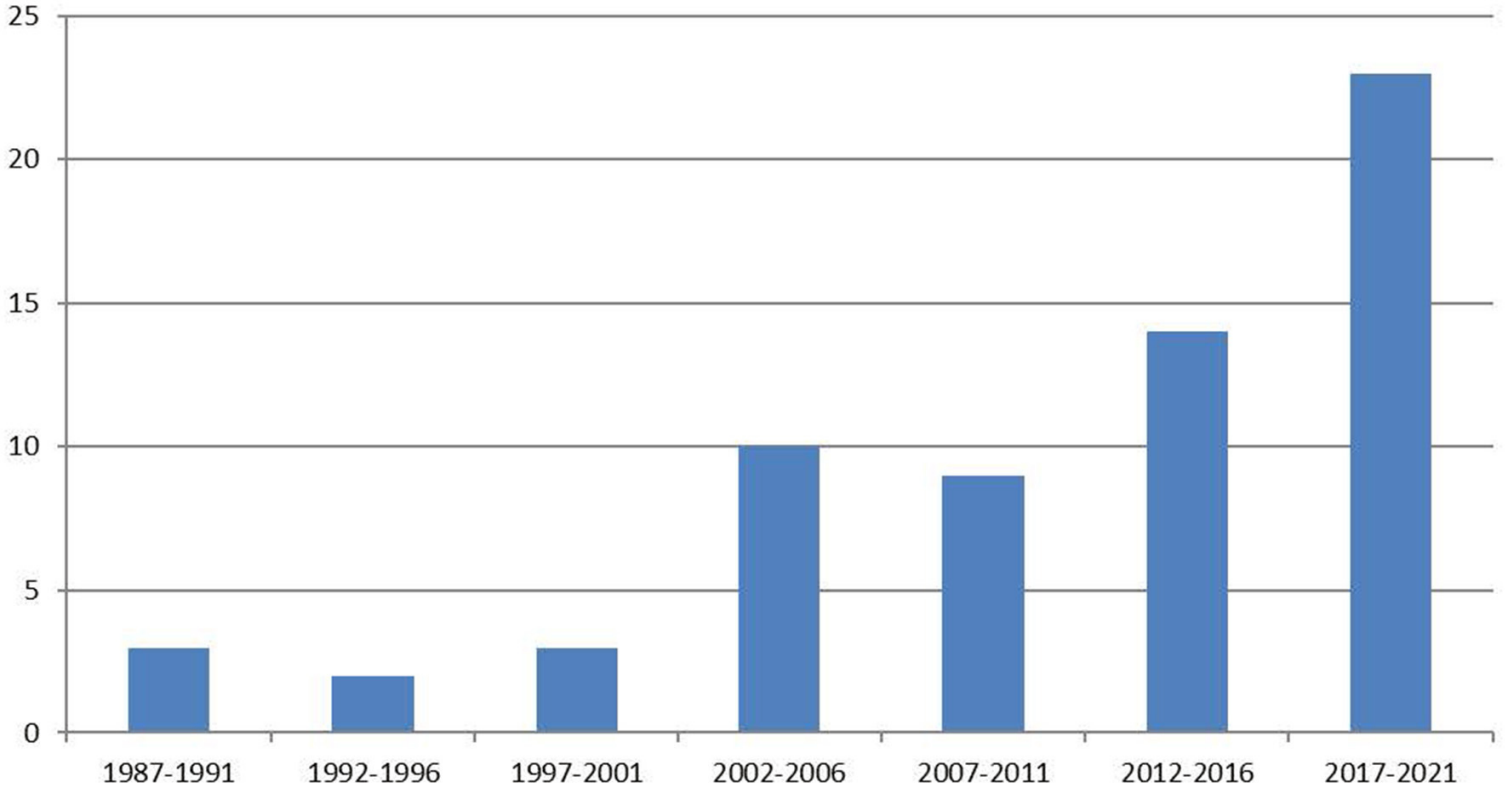
Number of Working Alliance Inventory measurement properties studies over time.

The database search resulted in 4,762 studies. After removing duplicates, a total of 2,770 studies remained, of which 66 met the eligibility criteria for inclusion (Figure 1 and Supplementary Material 2). The frequency of studies published on measurement properties of the WAI has increased over time (Figure 2).

### Study Characteristics

Sample sizes of the 66 studies included in this review ranged from eight to 1,786 participants. Mean age ranged from six to 98 years (Supplementary Table 1). Measurement properties of the WAI were evaluated in 23 different countries and in 16 different languages. Most studies were performed in the USA (*n* = 22), followed by Spain (*n* = 7), and the Netherlands (*n* = 5). The WAI-measurement studies concerned 16 different professional contexts. The contexts of psychotherapy (*n* = 24) and psychology (*n* = 19) were the most frequent. Other contexts included physiotherapy (*n* = 14), education (*n* = 2), child protection service (*n* = 2), and general practice (*n* = 2).

After the first study by Horvath and Greenberg (1989), 44 different versions of the WAI have been developed. Reasons for adaption of the original study included usability in different contexts, reduction of items, and adaptation for different languages. In the different versions of the WAI, four types of answering scales were reported: a seven-point scale, six-point scale, five-point scale, and a Visual Analog Scale. The seven-point scale was the most frequently used. In most studies, relatively high mean scores were reported for both the domain scores and the total scores. Age and gender of participants varied depending on the target groups of the studies, but these variables were not taken into account in the assessment of the included studies. As advised by the COSMIN criteria variables concerning cross-cultural validity/measurement invariance received specific attention. An overview of the methodological evaluation of the WAI and adapted versions is shown in Table 2.

**Table 2:** Overview of the Methodological evaluation of the WAI and all adapted versions (in total *n* = 66).

				**Cont V**		**Struct C**		**Int C**		**Mea Inv**		**Reliab**		**Construct V**	
**Author (Publ. year)**	**WAI-Version**	**Setting**	**Country**	**ROB**	**RS**	**ROB**	**RS**	**ROB**	**RS**	**ROB**	**RS**	**ROB**	**RS**	**ROB**	**RS**
Horvath and Greenberg (1989)	WAI-P	Psychotherapy	Canada	I	-			D	±					D	±
	WAI-T			I	-			D	-						
Tichenor and Hill (1989)	WAI-P	Psychotherapy	USA					I	±						
	WAI-T							I	±						
	WAI-O			I	-			I	±			I	+	I	±
Tracey and Kokotovic (1989)	WAI-P	Psychotherapy	USA			I	-								
	WAI-T					I	-								
Hatcher et al. (1995)	WAI-P	Psychotherapy	USA			I	+							I	±
	WAI-T					I	+							I	±
Hatcher and Barends (1996)	WAI-P	Psychotherapy	USA			A	-							I	±
Hatcher (1999)	WAI-T	Psychotherapy	USA			D	-	D	±					D	±
Andrusyna et al. (2001)	WAI-S-O	Psychology	USA			A	-								
Cecero et al. (2001)	WAI-P	Psychology	USA					V	±						
	WAI-T							V	±						
	WAI-O							V	±			I	+	I	±
Fenton et al. (2001)	WAI-P	Psychology	USA												
	WAI-T														
	WAI-O											I	+	I	±
Stiles et al. (2002)	WAI-P	Psychology	USA											D	+
	WAI-T													D	+
	WAI-O													D	+
Busseri and Tyler (2003)	WAI-P	Psychology	USA					V	±					I	±
	WAI-T							V	±					I	±
	WAI-S-P							V	±					I	±
	WAI-S-T							V	±					I	±
Santibáñez (2003)	IAT-S-P	Psychotherapy	Chile			I	-	V	-						
	IAT-S-T					I	-	V	±						
Corbella and Botella (2004)	WATOCI	Psychotherapy	Spain			A	-	V	±						
Goldberg et al. (2004)	WAI-S-P	Psychiatric rehabilitation	USA					D	-			I	+		
	WAI-S-T							D	-			I	+		
Ely et al. (2005)	WAICC	Hematologic disorders	USA	D	-			I	±			I	-		
Guédeney et al. (2005)	WAI-P	Social work	France	I	-									I	±
	WAI-T			I	-									I	±
Bedregal et al. (2006)	TAC	Psychology	USA	I	-	D	-							I	+
Corbiére et al. (2006)	WAI-S-P	Psychotherapy	Canada	D	-			V	-						
	WAI-S-T			D	-			V	±						
Hatcher and Gillaspy (2006)	WAI-SR-P	Psychotherapy	USA			A	-	V	±	D	±			I	±
Soygüt and Işikli (2008)	WAI-P	Psychotherapy	Turkey	I	-	I	-	A	±					I	±
	WAI-T			I	-	I	-	A	±					I	±
Wilmers et al. (2008)	WAI-SR-P	Psychotherapy	Germany	I	-	V	-							D	±
Soygüt and Uluc (2009)	WAI-O	Psychotherapy	Turkey	I	-	I	-	I	±			I	+		
Stinckens et al. (2009)	WAV-12-P	Psychotherapy	Belgium			V	-	D	±					I	±
Munder et al. (2009)	WAI-SR-P	Psychotherapy	Germany			V	-	V	±	I	+			I	±
Perdrix et al. (2010)	WAI-SR-P	Career counseling	Switzerland	I	-	A	-	V	±						
Tatman and Love (2010)	WAI-SR-P	Offender therapy	USA			V	-	D	±			D	±	D	-
Corbella et al. (2011)	WAI-S-P	Psychotherapy	Spain			A	-	V	±						
Ross et al. (2011)	WAI-S-P	Offender therapy	New Zealand			I	-								
	WAI-S-T					I	-								
	WAI-S-O					I	-								
Hall et al. (2012)	WATOCI	Physiotherapy	Australia			V	±	I	-						
Vóhringer et al. (2013)	WAI-O	Psychotherapy	Chile	I	-			D	±					I	?
Andrade-González and Fernández-Liria (2015)	WAI-P	Psychology	Spain	I	-			D	±					I	±
	WAI-T			I	-			D	±					I	±
Falkenström et al. (2015a)	WAI-SR-P	Psychology	Sweden/USA			V	±			D	-				
Falkenström et al. (2015b)	SAI-P	Psychology	Sweden/USA			D	±	V	±	D	±			D	+
Lamers et al. (2015)	WAI-12-P	Residential psychiatry	Netherlands	I	-	A	-	A	±					D	+
	WAI-12-Team			I	-	A	-	A	±					D	+
Miragall et al. (2015)	WAI-VAR-P	Psychotherapy	Spain	I	-	A	-	A	±					I	±
														I	±
Smits et al. (2015)	WAV-12-S-P	Psychotherapy	Belgium			V	-	D	±					D	-
Toste et al. (2015)	CWAI-P	Education	USA			V	+							D	±
														D	±
	CWAI-T					V	-								
Andrade-González et al. (2016)	WAI-S-P	Psychology	Spain					D	±					I	±
	WAI-S-T							D	±					I	±
Figueiredo et al. (2016)	WAI-CA-P	Psychology	Portugal	I	-			V	±					I	±
Hukkelberg and Ogden (2016)	WAI-S-P	Psychology	Norway			V	-								
Hsu et al. (2016)	WAI-S-P	Psychology	Hong Kong			D	-					I	-		
Mallinckrodt and Tekie (2016)	BAI-P	Psychotherapy	USA			V	±	D	±					I	±
Araujo et al. (2017)	WAI-S-P	Physiotherapy	Brazil	I	-			D	-			I	±	I	±
	WAI-S-T			I	-			D	-			I	±	I	±
Hukkelberg and Ogden (2017)	WAI-S-P	Psychology	Norway			V	+	V	+						
Hsu and Yu (2017)	WAI-S-T	Psychology	Honk Kong			I	-	V	±						
Killian et al. (2017)	WAI-S-P	Child protection service	USA			D	-	V	±					I	±
	WAI-S-T					D	-	V	±					I	±
	WAI-S-O					D	-	V	±					I	±
Bat Or and Zilcha-Mano (2018)	AT-WAI-P	Art therapy	Israel	I	-	A	-	V	±					I	±
Chen et al (2018)	WAI-SR-P	Psychotherapy	China	I	-			V	±					I	±
Gulum et al (2018)	WAI-S-P	Psychotherapy	Turkey			I	-	D	-						
	WAI-S-T					I	-	D	-						
Karel et al. (2018)	WAV-12-P	Physiotherapy	Netherlands	I	-	V	-	V	±						
Paap et al. (2018)	WAI-SR-P-ReD	Rehabilitation	Netherlands	D	-	V	-	V	±					A	±
Santirso et al. (2018)	WAI-S-O	Psychotherapy	Spain			D	±	V	±			A	+	I	±
Sturgiss et al. (2018)	WAI-P-GP	General practice	Australia	D	±	D	±	V	±					I	+
Takasaki et al. (2019)	WAI-S-P	Physiotherapy	Japan			D	±	V	±			D	±		
Penedo et al. (2019)	WAI-I-P	Psychology	Switzerland	D	-	V	-	V	±					V	±
Paap et al. (2019)	WAI-SR-P-ReD	Rehabilitation	Netherlands											V	+
Petek et al (2019)	WAI-SR-P	Family medicine	Slovenia	D	-										
	WAI-SR-T			D	-										
Warlick et al. (2018)	WAIT-12-P	Tobacco counseling	USA	I	-	V	-	V	±			I	+	D	+
	WAIT-3-P			I	-			V	±			I	+		
Hatcher et al. (2020)	WAI-S-T-IRT	Psychology	USA			V	±			D	-				
Herrero et al. (2020)	WAI-SR-TECH	Psychology	Multi center	I	-	A	-							D	+
Hunik et al. (2021)	WAI-P-GP	General practice	Australia			I	-	V	±					I	+
Miloff et al. (2020)	VTAS-P	Psychology	Sweden	I	-	I	-	A	-					D	±
Milot-Lapointe et al. (2020)	WAI-S-P	Career counseling	Canada			I	-	V	-	D	±				
Knowles et al. (2020)	CWAI-P	Education	USA			V	+	D	±						
	CWAI-T					V	-	D	±						
Cirasola et al. (2021)	WAI-S-P	Youth psychotherapy	UK			V	-			D	±				
	WAI-S-T					V	-			I	±				
Prusinski (2021)	WAI-P	Psychotherapy	Poland	I	-	V	-	V	±						
	WAI-T			I	-	V	-	V	±						

*Cont V, Content validity; Constr V, Construct validity; Cross, cross-sectional study; Int C, Internal Consistency; Long, Longitudinal study; Mea Inv, Measurement invariance; Reliab, reliability; Struct V, Structural validity; ROB, Risk of Bias (Methodological quality) according to the COSMIN checklist (Mokkink et al., 2016), assessed as V, very good; A, adequate; D, doubtful; or I, insufficient. RS, Rating score of the measurement property according to the COSMIN criteria (Prinsen et al., 2018), see Table 1; rated as +, sufficient. ?, Indeterminate. -, insufficient. WAI-Versions, AT-WAI, Art Therapy-Working Alliance Inventory; BAI, Brief Alliance Inventory; CWAI, Classroom Working Alliance Inventory; IAT, Inventario de Alianza de Trabajo; O, Observer form or observer; P, Patient form or patient (Patients included students, sex offenders, parents, rehabilitation patients, psychiatry patients, parents, families and so forth, anyone who was the client receiving treatment); TAC, Therapeutic Alliance with Clinician; TSF, Twelve-step facilitation; Transl, Translation study; VTAS, Virtual Therapist Alliance Scale; WAI-I, Working Alliance Inventory Internet interventions; WAI-IRT, Working Alliance Inventory Item Response Theory; WAICC, Working Alliance Inventory for Chronic Care (20 versions); WAI-CA, Working Alliance Inventory for Children and Adolescents; WAI, Working Alliance Inventory; WAI-GP, Working Alliance Inventory General Practice; WAI-ReD, Working Alliance Inventory Rehabilitation Dutch Version; WAI-S, Working Alliance Inventory Short Form; WAI-SR, Working Alliance Inventory Short Form Revised; WAI-SR-TECH, Working Alliance Inventory Short Form Revised for online Interventions; WAI-VAR, Working Alliance Inventory applied to virtual and augmented reality; WAIT, Working Alliance Inventory for Tobacco; WAV-12, Werk Alliantie Vragenlijst; WATOCI, Working Alliance Theory of Change Inventory. WAIT, Working Alliance Inventory for Tobacco*.

### Content Validity

Content validity concerns “the degree to which the content of a measurement instrument is an adequate reflection of the construct to be measured” (Supplementary Table 2) (Mokkink et al., 2010b).

After the first development and content validity study by Horvath & Greenberg in 1989, 25 studies evaluated content validity of the WAI, including 32 adapted version (Supplementary Table 2). Other studies (*n* = 12) reduced the number of items or did not analyze content validity. Therefore, these studies were not included in Supplementary Table 2. The first WAI study was performed according to the standards of that time (Horvath, 2018). However, based on current COSMIN criteria, this original study is now assessed as inadequate. The context of use lacked a clear description. The sample size of the quantitative study did not meet current standards, and a qualitative study was not conducted prior to the development of the quantitative study. Furthermore, the sample of the pilot test (*n* = 29 graduate students) did not represent the target population (psychotherapy clients). Also, the WAI concerns a patient-reported measure, and in the original study participants were not asked about the relevance, comprehensibility, and comprehensiveness of the WAI (Horvath and Greenberg, 1989).

The methodological quality of the 25 content validity studies was assessed as doubtful (*n* = 5) and inadequate (*n* = 20) (Supplementary Table 2). The main reason for these ratings was a general lack of involvement of patients and professionals in the evaluation of aspects of content validity. In nine of the 25 studies, patients were involved in evaluating at least one of the aspects relevant to content validity (relevance, comprehensibility, and/or comprehensiveness). One study evaluated all aspects of content validity (Sturgiss et al., 2018). Although three studies used a qualitative approach during the development phase, they lacked detail on the exact qualitative method applied (Figueiredo et al., 2016; Karel et al., 2018; Paap et al., 2018).

In four studies ceiling effects were explicitly reported (Hukkelberg and Ogden, 2016; Araujo et al., 2017; Paap et al., 2018; Takasaki et al., 2019). Two of these studies reported ceiling effects in all items of the WAI (Araujo et al., 2017; Takasaki et al., 2019); one study reported ceiling effects in half of the items (Hukkelberg and Ogden, 2016); and one study reported ceiling effects in all domain scores of the WAI (Paap et al., 2018).

In conclusion, based on the COSMIN criteria for content validity, none of the included studies could be rated sufficient. This means that evidence for content validity currently is unknown.

### Internal Structure

Internal structure refers to “how the different items of the measurement instrument are related, which is important to know for deciding how items might be combined into a scale or subscale” (Prinsen et al., 2018). In case of the WAI, internal structure concerns aspects of the working alliance, comprising goals, tasks, and bond. In this review, several aspects of internal structure were examined: structural validity (3.4.1), internal consistency (3.4.2), and cross-cultural validity and measurement invariance (3.4.3) (Supplementary Tables 3–5).

#### Structural Validity

Structural validity refers to “the degree to which the scores of a measurement instrument are an adequate reflection of the dimensionality of the construct to be measured” (Mokkink et al., 2010b).

Fifty-one studies reported on structural validity (the dimensionality) of the WAI. In total, 73 analyses were performed, including confirmative (*n* = 49), explorative (*n* = 21), and Rasch-analyses (*n* = 3) (Supplementary Table 3). The methodological quality of 29 studies was rated as very good, 14 studies were rated adequate, 15 studies doubtful, and 15 inadequate. Doubtful scores were mainly caused by lack of information concerning methods used to assess structural validity and handling of missing data. Inadequate ratings were mostly caused by small sample sizes.

Confirmatory factor analysis to determine the best-fitting structure of the WAI and all adapted versions was conducted in 49 studies. Results were conflicting. A three-factor structure was reported in 22 studies, of which 10 had good methodological quality. A two-factor structure was reported in 16 studies, of which 11 had good methodological quality. A bi-level structure was reported in seven studies, of which five had good methodological quality. Finally, a one-factor structure was found in two studies; both had good methodological quality. Conflicting results on the best-fitting model for a factor structure of the WAI were found in the psychotherapy context and other healthcare contexts.

In total, four out of 51 studies reported an adequate model fit, according to the COSMIN criteria for sufficient structural validity (Hatcher et al., 1995; Toste et al., 2015; Hukkelberg and Ogden, 2017; Knowles et al., 2020). The study of Hatcher et al. (1995) confirmed a three-factor structure (bond, task, and goal) as the best-fitting model; however, the methodological quality of this study was inadequate because of its small sample size. The other three studies with an adequate fit confirmed a bi-factor structure (hierarchical model) or a two-factor structure (*n* = 2) as the best-fitting model. The methodological quality of these studies was very good (Toste et al., 2015; Hukkelberg and Ogden, 2017; Knowles et al., 2020). The methodological quality of six studies concerning structural validity was rated indeterminate (in Supplementary Table 3 indicated with a ?), because they did not report on criteria information.

#### Internal Consistency

Internal consistency refers to “the degree of interrelatedness among items” (Mokkink et al., 2010b). A total of 52 studies reported on internal consistency; 72 analyses were conducted in these studies (Supplementary Table 4). The methodological quality of 30 studies was rated as very good. Four studies were rated adequate (due to sample sizes *n* < 100. Sixteen studies were rated doubtful due to small sample sizes (*n* < 50), lack of clarity on structural validity, or because internal consistency statistics were not calculated for each domain of the WAI. Two studies were rated inadequate because no Cronbach's alpha or omega were presented.

The criteria for sufficient internal consistency are a Cronbach's alpha ≥ 0.70 and at least low evidence for sufficient structural validity (sufficient validity = sufficient model fit, tested with confirmatory factor analyses or IRT/RASH analyses). Only one study met both criteria for internal consistency (Hukkelberg and Ogden, 2017). In 64 different analyses of the WAI and its adapted versions a Cronbach's alpha > 0.70 was found. However, because criteria for sufficient structural validity were not met, these studies were rated indeterminate. Seven studies found a Cronbach's alpha < 0.70 (insufficient). Consequently, although many studies reported strong interrelatedness of the items of the WAI and all adapted versions, internal consistency could not be established.

#### Cross-Cultural Validity/Measurement Invariance

Cross-cultural validity/measurement invariance refers to “the degree to which the performance of the items on a translated or culturally adapted measurement instrument are an adequate reflection of the performance of the items of the original version of the measurement instrument” (Mokkink et al., 2010b).

Seven studies analyzed cross-cultural validity/measurement invariance. Four studies tested model fit across different study samples. Three studies tested longitudinal measurement invariance across different treatment sessions (Supplementary Table 5). In six out of seven studies, the methodological quality was rated as doubtful, due to lack of clarity on relevant group characteristics and lack of information on the statistical method used. One study was rated inadequate, due to the small sample size (Munder et al., 2009). Three studies (Hatcher and Gillaspy, 2006; Munder et al., 2009; Falkenström et al., 2015a) assessed model fit across different study samples of the WAI-SR-Patient form (P) within the context of psychotherapy. The results of these three studies were conflicting. The first study reported a sufficient fit, but its methodological quality was rated as doubtful. The second study found no differences across the different study samples, but the methodological quality of this study was rated as inadequate. The third study found differences in fit across different samples, but the methodological quality was rated as doubtful.

One study analyzed the invariance between different versions of the WAI-S-Therapist form (T), WAI-SR-T and a WAI-S-T-item response theory version, in diverse study samples (Hatcher et al., 2020). All three measures showed sufficient fit and were confirmed in the other samples. The WAI-S-T- Item Response Theory (IRT) version fit was slightly better, but differences were minor. No version showed sufficient measurement invariance. However, the methodological quality of this study was doubtful.

Two studies, both with doubtful methodological quality, analyzed longitudinal measurement invariance of the WAI-S-P (Milot-Lapointe et al., 2020; Cirasola et al., 2021). Both studies found no significant differences across treatment sessions. The longitudinal measurement invariance of Session Alliance Inventory Patient Form (SAI-P) was tested within 10 different treatment sessions. The first session was not included in one study (with doubtful methodological quality). Factor loadings were stable, except for a few minor deviations (Falkenström et al., 2015b).

Altogether, seven studies reported on measurement invariance of the WAI. The results of these studies were inconsistent. Only one study was rated sufficient, but its methodological quality was rated as inadequate (Munder et al., 2009). The other studies were rated indeterminate (*n*=5*)* or insufficient (*n* = 2).

### Remaining Measurement Properties

#### Reliability and Measurement Error

Reliability refers to “the extent to which scores for clients/therapists/observers who have not changed are the same for repeated measurement under several conditions, e.g., using different sets of items from the same measurement instrument (internal consistency) over time (test-retest), by different persons on the same occasion (inter-rater), or by the same persons (i.e., raters or responders) on different occasions (intra-rater)” (Mokkink et al., 2010b).

Twelve studies reported on reliability and/or measurement error of the WAI and its adapted versions (Supplementary Table 6). Four studies tested inter-rater reliability and eight studies tested test-retest reliability. The methodological quality of one study was rated as adequate (Santirso et al., 2018). All other studies were rated inadequate (*n* = 9*)* or doubtful (*n* = 2), because small sample sizes (*n* = 6) or because the interval between assessments was not appropriate (*n* = 3). Example of inappropriate interval; studies with a measurement interval of 1 or 2 months, several treatment sessions between the measurements might have taken place, interference of such in-between sessions can be assumed, and therefore such a study may be rated as inappropriate. Studies were scored doubtful due to lack of information on several factors, including time interval, type of intra-class correlation coefficient (ICC), or use of a Pearson correlation coefficient (Tatman and Love, 2010; Hsu et al., 2016).

All four studies that tested inter-rater reliability (using WAI-(short) observer form) reported sufficient reliability of the WAI. Also, all studies testing test-retest reliability reported sufficient reliability (ICCs >0.70). Two studies testing test-retest reliability did not calculate an ICC (Ely et al., 2005; Hsu et al., 2016). In conclusion, reliable findings were reported for stability of scores over time, but evidence was limited for test-retest reliability and inter-rater reliability.

#### Construct Validity (Hypotheses Testing)

Construct validity refers to “the degree to which the scores of a measurement instrument are consistent with hypotheses, for instance, with regard to internal relationships, relationships to scores of other instruments, or differences between relevant groups, based on the assumption that the measurement instrument validly measures the construct to be measured” (Mokkink et al., 2010b).

A total of 40 studies analyzed construct validity of the WAI and its adapted versions (Supplementary Table 7). Most studies provided evidence for construct validity by testing associations of the WAI with other related measurements and/or association with treatment outcomes. For both types of associations heterogeneity in comparisons was found. More than 70 different comparator instruments were used. The methodological quality of most of these studies was rated as inadequate (*n* = 24) or doubtful (*n* = 10). Only few studies were rated adequate (*n* = 1) or very good (*n* = 2; Paap et al., 2019; Penedo et al., 2019). However, the study rated as adequate lacked a description of important characteristics of subgroups such as age, gender, and context setting (Paap et al., 2018). Studies were rated as inadequate due to lack of reporting on prior hypotheses, lack of clarity on how the hypotheses were tested regarding construct validity, and lack of information on measurement properties of the comparator instrument or the use of inappropriate statistical methods. Studies were rated as doubtful when hypotheses were formulated that could be interpreted in different ways.

Hypotheses on convergent, divergent, and discriminative validity were formulated in 22 studies. In 10 studies, hypotheses were confirmed; in six studies hypotheses regarding convergent validity were confirmed; in eight studies hypotheses regarding divergent validity were confirmed; and in one study hypotheses regarding discriminative validity were confirmed (Stiles et al., 2002; Bedregal et al., 2006; Falkenström et al., 2015b; Lamers et al., 2015; Sturgiss et al., 2018; Warlick et al., 2018; Paap et al., 2019). The methodological quality of studies in which hypotheses were confirmed was rated as inadequate (*n* = 3) (Bedregal et al., 2006; Sturgiss et al., 2018; Hunik et al., 2021), doubtful (*n* = 6) (Stiles et al., 2002; Falkenström et al., 2015b; Lamers et al., 2015; Warlick et al., 2018; Herrero et al., 2020; Miloff et al., 2020) or very good (*n* = 1) (Paap et al., 2019). In two studies (Tatman and Love, 2010; Smits et al., 2015), hypotheses on divergent validity were rejected. Methodological quality of these studies was rated as doubtful, because of lack of description about important characteristics of subgroups and lack of clarity on how hypotheses were tested. Other studies formulated no (*n* = 18) or ambiguous hypotheses (*n* = 10). In three studies, the results of testing hypotheses were conflicting; some hypotheses were confirmed others were not (Smits et al., 2015; Toste et al., 2015; Paap et al., 2018). Therefore, the overall rating for construct validity of the studies that tested hypotheses was rated as indeterminate.

In conclusion, there is limited evidence for convergent validity, conflicting evidence for divergent validity, and unknown evidence for discriminative validity of the WAI and its adapted versions.

#### Responsiveness

Responsiveness concerns “the ability of the measurement instrument to detect change over time in the construct to be measured” (Mokkink et al., 2010b).

Because only one study analyzed responsiveness of the WAI-S-P (Araujo et al., 2017), responsiveness was not included in the overview of the evaluation (Table 2). Furthermore, the quality of that particular study was rated as inadequate, because no prior hypotheses were formulated, information on the construct measured by the comparator instrument was lacking, and information on the measurement properties of the comparator instrument was missing (Supplementary Table 8). Consequently, the quality of responsiveness of the WAI could not be determined in this review.

## Discussion

For several decades now, the WAI has been a widely used instrument, aimed to measure the perceived strength of the working alliance in psychotherapy and in several other healthcare contexts. To establish an overview of measurement properties of the WAI, this systematic review included studies that analyzed measurement properties of both the original versions of the WAI, and the versions adapted for other healthcare contexts. The review was conducted according the COSMIN criteria that were specifically developed to guide systematic reviews of studies on measurement properties (Prinsen et al., 2018). A total of 66 studies published between 1989 and 2021 were included. The publication-rate of such studies has increased remarkably over time, which underscores the importance of an overview of the measurement properties of the WAI. Most of the included studies did not, or only partially, meet the COSMIN criteria. Evidence for measurement properties was insufficient, lacking, or conflicting. Content validity was generally rated insufficient, because neither patients nor healthcare professionals were involved in the evaluation process; therefore, evidence for this aspect remains unknown. Conflicting evidence was found for the structural validity of the WAI. Evidence concerning internal consistency could not be established. Limited evidence was found for inter-rater reliability and convergent validity. Conflicting evidence was found for test-retest reliability and divergent validity. In conclusion, analysis of 66 studies that reported on measurement properties of the WAI and its adapted versions showed that they were generally not in agreement with current COSMIN criteria (Prinsen et al., 2018).

### Content Validity of the WAI

To interpret the findings regarding content validity of the WAI, it is important to keep in mind that the WAI intends to embody Bordin's (1979) theory of the working alliance. This theory concerns the structure and functioning of the working relationship in terms of goals, tasks, and the therapeutic bond. Therefore, Horvath & Greenberg asked non-patients, including alliance researchers, to determine whether the questions reflected the dimensions of this theory in their item development of the WAI (Horvath and Greenberg, 1989). Besides input from experts, the original item development was not based on qualitative research or on cognitive interviews with the target population (i.e., psychotherapy clients) to determine the content validity of the WAI regarding relevance, comprehensibility, and comprehensiveness of the items of the WAI. Although during the development of the WAI researchers were of course not informed by the current (COSMIN) criteria, the theoretical base of the WAI remains a fundamental problem. This problem is reflected in the outcomes of this systematic review; because all adapted versions of the WAI are based on the original theory-based items, the content validity of the WAI and its adapted versions could not be confirmed in this systematic review, and consequently had to be rated as unknown.

Content validity is considered to be the most important measurement property of a questionnaire (Terwee et al., 2018). If no or insufficient evidence is available regarding content validity, it remains unknown whether the instrument adequately reflects the underlying construct that it intends to measure. As a consequence, the interpretation of all other results is difficult, and the generalizability of various study findings is hampered (Mokkink et al., 2016). Furthermore, it should be noted that in many of the included studies ceiling effects were probably present; such studies show high mean scores combined with large standard deviations. Ceiling effects are present when more than 15% of the participants achieve the highest possible score (McHorney and Tarlov, 1995). When ceiling effects are present, participants with the highest scores cannot be distinguished (de Vet et al., 2011). Further, ceiling effects can be an indication that the content validity of the measurement instrument is not adequate (de Vet et al., 2011; Streiner et al., 2015).

In general, previous qualitative studies shows that patients report their subjective experiences of their working alliance (e.g., satisfaction, overall experiences and so forth) when they fill out an alliance measure concerning their treatment with a therapist (Bedi, 2006; MacFarlane et al., 2015; Paap et al., 2021). The outcome of the current review concerning content validity may be concerns not only the WAI, but may concern difficulties in content validity of alliance measures in general. The WAI is a theory-based measure; therefore, perhaps other criteria than the COSMIN criteria need to be developed and applied for appraising the quality of content validity. However, Strauss and Smith (2009) postulated that an analysis of the concept of validity should start from theories about the construct, followed by the formulation of hypotheses concerning relationships of that construct with other constructs, or hypotheses about values of that construct (de Vet et al., 2011). As noted by Horvath (2018), the construct working alliance and related constructs need to be clarified, because they remain theoretically unclear. Therefore, further development of theory concerning the (therapeutic) working alliance and related constructs is needed (Horvath, 2018). Empirical research can contribute to validation and further development of this theory.

Concerning content validity, qualitative research may offer insight into how respondents perceive the impact, value, and relevance of the working alliance. This approach can clarify whether the way clients/patients (or therapists) see themselves and their positions in psychotherapy or other healthcare contexts corresponds with Bordin's (1979) theory: do clients/patients see themselves as engaged in a mutual working relationship with their therapist with whom they share the same identified goals and tasks? Results of earlier qualitative research showed that clients/patients do not think this way about a working relationship (Bedi, 2006; MacFarlane et al., 2015; Paap et al., 2021). Furthermore, the WAI is applied in several other contexts besides psychotherapy, and the measurement studies in this systematic review all used the theory of Bordin concerning the working alliance. However, Bordin's theory may be is not generalizable to other healthcare contexts, such as rehabilitation, general practice, physiotherapy, or education. Therefore, not only the theory itself, but also its validation needs to be further explored in psychotherapy and other healthcare contexts.

### Internal Structure

The methodological quality of studies concerning internal structure was good or adequate in almost half of the studies. Their findings concerning the best fitting factor model for a structure were, however, quite heterogeneous. As a result, these findings are rated as inconsistent. Only four studies were in accordance with the COSMIN criteria for sufficient model fit for structural validity (Hatcher et al., 1995, Hukkelberg and Ogden 2017; Toste et al., 2015; Knowles et al., 2020). Three of these studies met all measurement criteria and found a bi-factor structure (hierarchical structure) and a two-factor structure to be the best-fitting model (Toste et al., 2015; Hukkelberg and Ogden, 2017; Knowles et al., 2020). Within the included studies no < 15 different criteria were applied to assess goodness of fit, which illustrates the heterogeneity regarding interpretation of outcomes concerning confirmatory factor analyses. However, COSMIN criteria for sufficient structural validity are very strict. When criteria of Hu and Bentler (1999) for an adequate fit would have been applied to the studies, thirteen studies instead of four would have fulfilled criteria (Hu and Bentler, 1999).

The strictness of COSMIN criteria concerning internal consistency (at least limited evidence for sufficient structural validity should be met) also affected the relatively lower ratings in this systematic review. However, studies mainly reported strong interrelatedness of the items, this may indicate a strong internal consistency.

Seven studies reported on measurement invariance, and the results of these studies were inconsistent. However, these studies were mainly rated lower due to lack of reporting on details. Therefore, the current review may underestimate the existing measurement invariance of the WAI and its adapted versions.

### Remaining Measurement Properties

In general, the reliability of the WAI was rated as good, but evidence for test-retest reliability and inter-rater reliability was limited, due to the methodological quality of the studies. For example, the risk of bias was rated higher in these studies due to lack of adequate reporting of ICCs. Only two out of 12 studies described which formula or model was used.

Construct validity was analyzed in 40 studies. In the majority of these studies, hypotheses were not explicitly and/or adequately described. Hypotheses such as predictions about the direction and magnitude of expected correlations were often lacking, which made it difficult to draw conclusions from those studies. Most studies based their evidence for construct validity on a correlation with another working alliance measure, or on therapeutic outcomes. However, Horvath (2018) already noted that this type of evidence is problematic for testing the construct validity of a theory, due to a lack of falsifiable hypotheses. Bordin (1979) suggested that the construct working alliance is generalizable to all types of healthcare professions and that differences in the working alliance occur predominantly in the domains of tasks and goals. In contrast, Flückiger et al. (2018) argued that the construct may benefit from a distinction between relational elements presumed to be common to all forms of therapy, and those specific relevant to a certain type of healthcare (Flückiger et al., 2018). Thus, first further theoretical development of the construct in general is needed, before its relevance or specific issues for other contexts than psychotherapy can be determined.

Since this review followed the COSMIN-approach, only studies that specifically aimed to investigate measurement properties of the WAI were included (de Vet et al., 2011; Prinsen et al., 2018). However, Flückiger et al. (2018) found more studies (*n* = 295; including over 30,000 participants) that reported correlations between strength of the alliance and outcomes. A majority of these studies used the WAI. Eventually, these studies might provide evidence for predictive validity, and some of them may also include potentially relevant information on other measurement properties (e.g., internal consistency). However, they were not included in this review because this would have increased the risk of bias; these studies were not primarily designed to investigate these measurement properties of the WAI. Including these studies would therefore probably not contribute to the level of evidence regarding measurement properties of the WAI. It would also require a full-text screening of all studies using the WAI and its adapted versions, which is a very time-consuming process that cannot be standardized easily (Prinsen et al., 2018). Finally, it would have led to an unwieldy number of studies for this systematic review, making it harder for future researchers to replicate this review (Prinsen et al., 2018; Jewell et al., 2019).

Although outcomes of the WAI have been assessed longitudinally in many studies (Xu and Tracey, 2015; Kivlighan Jr et al., 2016; David et al., 2021; Hasson-Ohayon et al., 2021), responsiveness could not be analyzed in the current, review because only one study investigated this measurement property (Araujo et al., 2017). More studies on responsiveness are needed, especially when the working alliance is considered to be a mediator and/or moderator of therapeutic change or treatment outcome.

### COSMIN Methodology in Psychotherapy Research

The COSMIN initiative aims to develop new and to update existing methodology criteria, based on broad consensus. The COSMIN criteria have been introduced only quite recently and were initially developed for use in biomedical healthcare and research, and for measuring constructs such as health related quality of life, symptom status, or functional status (Prinsen et al., 2018; Terwee et al., 2018). Recently, the methodology has also been used in systematic reviews in other healthcare contexts (Jewell et al., 2019; Harrison et al., 2020; Smith et al., 2021). Concerning this review, it should be taken in mind that many studies on measurement properties of the WAI (including the development study) were performed before the COSMIN criteria were published, which means that authors of earlier studies were not aware of these criteria and/or did not use them. Also, it has not yet been established whether these standards are generally suitable for all kinds of patient-(therapist) reported measures.

The COSMIN criteria take the measurement properties of PROMs as a starting point, with respect to the content validity of a given measure (Terwee et al., 2012). The WAI, on the other hand, intends to embody Bordin's theory of the working alliance. As such, there may be a (partial) lack of fit between research regarding measurement properties of a questionnaire like the WAI on the one hand, and the COSMIN criteria on the other. It should be taken in mind that, although scores of the WAI are frequently correlated to treatment outcomes, the WAI was from its beginning not intended to be an outcome measure. Furthermore, COSMIN criteria may be not properly tuned to instruments that measure social and interactional relationships, such as psycho- and psychological- therapeutic relationships. Therefore, we recommend for future research (for example in a Delphi study) to evaluate the COSMIN criteria for use in different types of patient reported measures, other than PROMs. For example, in the past years, adaptations to COSMIN criteria have been made for the fields of Rheumatology (Outcome Measures in Rheumatoid Arthritis Clinical Trials) (Boers et al., 2014), and Dermatology (Home Harmonizing Outcome Measures for us in Eczema) (Schmitt et al., 2014).

Still, based on the results of this review, discussion is needed concerning the meaning and relevance of the underlying construct of the WAI and, as a consequence, concerning its use as a mediating/moderating factor in treatment outcomes. Furthermore, content validity is a crucial criterion, and lack of evidence for this aspect poses a fundamental problem. Based on the results of this systematic review, it cannot be concluded which items do, or do not, reflect the construct of the WAI adequately. Assessment of the other measurement properties might provide indirect evidence for content validity. Also, insight into the quality of research regarding all measurement properties, according to adequate criteria, can help future investigators with conducting their research. Such developments may also imply discussion on the adequacy of COSMIN criteria for other fields than biomedical research. Last but not least, validation of a measurement instrument is an iterative process, in which results from previous studies are used in future studies to facilitate further development of theory as well as methodology. Such an approach may result in a stronger base for further validation of a construct and its measurement (de Vet et al., 2011).

### Limitations

Using the COSMIN criteria for reviewing the measurement properties of the WAI influenced this review and its outcomes. For example, these criteria make it difficult to distinguish between poor methodological quality vs. poor reporting of a study (Craxford et al., 2019). The criterion of counting the lowest score when assessing methodological quality of studies illustrates this issue. When a study is rated as very good on all but one criterion, which was rated inadequate, the overall score is inadequate, according to COSMIN criteria. In this review, it was not always clear whether the measurement of a specific methodological issue was not performed in a particular study, or simply not reported. In our opinion, researchers who plan to perform a study on measurement properties of a (already existing or new) scale should take into consideration all available measurement qualities.

Another limitation concerns the heterogeneity of measurement properties reported in the included studies, which did not provide the same amount of detail on every study included in this review. As a consequence, we may not have done justice to all efforts made in the past three decades of WAI research.

For the current review, the WAI was selected as a starter, because it is the most widely used questionnaire in research for measuring the working alliance, and its measurement properties are assessed most frequently (Horvath et al., 2011; Doran, 2016). Next to the WAI, there are over 70 other instruments that measure the construct working alliance (Flückiger et al., 2018), and new measures are being developed continuously. However, this review demonstrated that even a widely used questionnaire like the WAI may face fundamental problems concerning content validity. Adaptations to the procedures, as suggested by the COSMIN group, may be needed for patient (and/or therapist)-reported questionnaires concerning social phenomena, such as the working alliance. Thus, before reviewing measurement properties of all existing questionnaires concerning the working alliance, it is recommended that existing questionnaires are first reviewed on some key aspects (e.g., content validity, internal structure), before commencing an in-depth and time-consuming analysis of the total of measurement properties of all available measurement studies.

### Implications and Recommendations for the Future

The results of this systematic review have several implications. First, based on the findings concerning the measurement of the construct working alliance, there is a need for further development of a theoretical framework regarding the construct and subsequently the measurement of the working alliance (including other contexts than psychotherapy). Theory and validity testing is an iterative process in which tests of partially developed theories provide information that leads to elaboration as well as refinement of theory, which in turn provides a more sound basis for subsequent development of theory and construct validation (Strauss and Smith, 2009). In the majority of the studies included in this review, hypotheses regarding evaluating construct validity were not explicitly and/or adequately described. Therefore, to be able to develop robust measurement theory regarding the construct of the working alliance it is important in further research to formulate explicitly testable hypotheses. Second, to clarify the content validity of the WAI, there is a need for qualitative content validity studies. Within the working alliance, at least two parties (client/patient and therapist) play a complex and dynamic role. These parties each have fundamentally different positions and frames of references. The COSMIN group developed criteria for evaluating the content validity in patient-reported measures; these criteria can be used to increase the quality and the comparability of (content validity) measurement studies (Terwee et al., 2018). Third, this study highlights the relatively poor reporting in many of the included studies concerning measurement properties, on issues that are necessary according to current standards. Recently, the COSMIN group developed a reporting guideline, which can be generally used to improve reporting and which also promotes comparability of studies (Gagnier et al., 2021). Fourth, insufficient or low-quality evidence for measurement properties of the WAI, as appraised by the COSMIN criteria, should not be interpreted as evidence for insufficiency. Furthermore, the issue of measurement quality does not only concern measurement studies of the WAI; measurement properties of other alliance instruments should be evaluated as well, and comparisons between these measurements are required. Last, although COSMIN criteria are promising for the purpose of increasing the quality of reviews of PROMs, a discussion is needed on the question if and why COSMIN criteria are adequate for evaluation of measurement properties of instruments within the context of psychological research. However, first a more fundamental methodological discussion regarding the concept of validity is needed. The findings of this review may provide a starting point for such a discussion. Thereafter, an international consensus study, for example using the Delphi method, might be suitable to examine the appropriateness of using the current or an eventually adapted COSMIN criteria in the context of psychological research.

## Data Availability Statement

The original contributions presented in the study are included in the article/Supplementary Material, further inquiries can be directed to the corresponding author.

## Author Contributions

All authors listed have made a substantial, direct, and intellectual contribution to the work and approved it for publication.

## Conflict of Interest

The authors declare that the research was conducted in the absence of any commercial or financial relationships that could be construed as a potential conflict of interest.

## Publisher's Note

All claims expressed in this article are solely those of the authors and do not necessarily represent those of their affiliated organizations, or those of the publisher, the editors and the reviewers. Any product that may be evaluated in this article, or claim that may be made by its manufacturer, is not guaranteed or endorsed by the publisher.
