# Supplementary material for: The Working Alliance Inventory's Measurement Properties: A Systematic Review
Source: Front Psychol. 2022 Jul 15;13:945294. doi: 10.3389/fpsyg.2022.945294 (PMC9337219; doi:10.3389/fpsyg.2022.945294)
Supplement: Supplementary file 1 [file Data_Sheet_1.docx]

**Supplementary Material**

**Supplementary Material S1.** Search strategies.

**Pubmed**

(working alliance*[tiab] OR therapeutic alliance*[tiab] OR helping alliance*[tiab])
AND
(inventory[tiab] OR inventories[tiab] OR short[tiab] OR revised[tiab] OR questionnair*[tiab] OR score*[tiab] OR rating*[tiab] OR scale*[tiab])
AND
((instrumentation[sh] OR methods[sh] OR validation studies[pt] OR Comparative Study[pt] OR psychometrics[MH] OR psychometr* [tiab] OR clinimetr*[tw] OR clinometr*[tw] OR “outcome assessment (health care)”[MH] OR “outcome assessment”[tiab] OR “outcome measure*”[tw] OR “observer variation”[MH] OR “observer variation”[tiab] OR “Health Status Indicators”[MH] OR “reproducibility of results”[MH] OR reproducib*[tiab] OR “discriminant analysis”[MH] OR reliab*[tiab] OR unreliab*[tiab] OR valid*[tiab] OR coefficient[tiab] OR homogeneity[tiab] OR homogeneous[tiab] OR “internal consistency”[tiab] OR (cronbach*[tiab] AND (alpha[tiab] OR alphas[tiab])) OR (item[tiab] AND (correlation*[tiab] OR selection*[tiab] OR reduction*[tiab])) OR agreement [tiab] OR precision[tiab] OR imprecision[tiab] OR “precise values”[tiab] OR testeretest[tiab] OR (test[tiab] AND retest[tiab]) OR (reliab*[tiab] AND (test[tiab] OR retest[tiab])) OR stability[tiab] OR interrater[tiab] OR inter-rater[tiab] OR intrarater[tiab] OR intrarater[tiab] OR intertester[tiab] OR inter-tester[tiab] OR intratester [tiab] OR intra-tester[tiab] OR interobserver[tiab] OR inter-observer [tiab] OR intraobserver[tiab] OR intraobserver[tiab] OR intertechnician[tiab] OR inter-technician[tiab] OR intratechnician[tiab] OR intra-technician[tiab] OR interexaminer[tiab] OR interexaminer[tiab] OR intraexaminer[tiab] OR intra-examiner[tiab] OR interassay[tiab] OR inter-assay[tiab] OR intraassay[tiab] OR intra-assay[tiab] OR interindividual[tiab] OR inter-individual[tiab] OR intraindividual[tiab] OR intra-individual[tiab] OR interparticipant[tiab] OR inter-participant[tiab] OR intraparticipant [tiab] OR intra-participant[tiab] OR kappa[tiab] OR kappa’s[tiab] OR kappas[tiab] OR repeatab*[tiab] OR ((replicab*[tiab] OR repeated [tiab]) AND (measure[tiab] OR measures[tiab] OR findings[tiab] OR result[tiab] OR results[tiab] OR test[tiab] OR tests[tiab])) OR generaliza*[tiab] OR generalisa*[tiab] OR concordance[tiab] OR (intraclass[tiab] AND correlation*[tiab]) OR discriminative[tiab] OR “known group”[tiab] OR factor analysis[tiab] OR factor analyses [tiab] OR dimension*[tiab] OR subscale*[tiab] OR (multitrait[tiab] AND scaling[tiab] AND (analysis[tiab] OR analyses[tiab])) OR item discriminant[tiab] OR interscale correlation*[tiab] OR error[tiab] OR errors[tiab] OR “individual variability”[tiab] OR (variability[tiab] AND (analysis[tiab] OR values[tiab])) OR (uncertainty[tiab] AND (measurement[tiab] OR measuring[tiab])) OR “standard error of measurement”[tiab] OR sensitiv*[tiab] OR responsive*[tiab] OR ((minimal[tiab] OR minimally[tiab] OR clinical[tiab] OR clinically [tiab]) AND (important[tiab] OR significant[tiab] OR detectable [tiab])AND (change[tiab] OR difference[tiab])) OR (small*[tiab] AND (real[tiab] OR detectable[tiab]) AND (change[tiab] OR difference [tiab])) OR meaningful change[tiab] OR “ceiling effect”[tiab] OR “floor effect”[tiab] OR “Item response model”[tiab] OR IRT[tiab] OR Rasch[tiab] OR “Differential item functioning”[tiab] OR DIF[tiab] OR “computer adaptive testing”[tiab] OR “item bank”[tiab] OR “crosscultural equivalence”[tiab]))

**PsychInfo**
((working OR therapeutic OR helping) N1 alliance*)

AND

(inventory OR inventories OR short OR revised OR questionnair* OR score* OR rating* OR scale*)
AND
(instrumentation OR “validation studies” OR “comparative study” OR psychometrics OR psychometr* OR clinimetr* OR clinometr* OR “outcome assessment” OR “outcome assessment” OR “outcome measure*” OR “observer variation” OR “observer variation” OR “health status indicators” OR “reproducibility of results” OR reproducib* OR “discriminant analysis” OR reliab* OR unreliab* OR valid* OR coefficient OR homogeneity OR homogeneous OR “internal consistency” OR cronbach* OR alpha OR alphas OR item OR selection* OR reduction* OR agreement OR precision OR imprecision OR “precise values” OR testeretest OR retest OR reliab* OR retest OR stability OR interrater OR inter-rater OR intrarater OR intrarater OR intertester OR inter-tester OR intratester OR intra-tester OR interobserver OR inter-observer OR intraobserver OR intraobserver OR interexaminer OR interexaminer OR intraexaminer OR intra-examiner OR interassay OR inter-assay OR intraassay OR intra-assay OR interindividual OR inter-individual OR intraindividual OR intra-individual OR interparticipant OR inter-participant OR intraparticipant OR intra-participant OR kappa* OR repeatab* OR replicab* OR repeated OR concordance OR intraclass OR discriminative OR “known group” OR “factor analysis” OR “factor analyses” OR dimension* OR subscale* OR multitrait OR scaling OR “item discriminant” OR “interscale correlation*” OR error OR errors OR “individual variability” OR variability OR measurement OR measuring OR “standard error of measurement” OR sensitiv* OR responsive* OR detectable OR “meaningful change” OR “ceiling effect” OR “floor effect” OR “item response model” OR IRT OR rasch OR “differential item functioning” OR DIF OR “computer adaptive testing” OR “item bank” OR “crosscultural equivalence”)

**Embase**((working OR therapeutic OR helping) NEXT/1 alliance*):ab,ti,de
AND
(inventory OR inventories OR short OR revised OR questionnair* OR score* OR rating* OR scale*):ab,ti,de
AND
(instrumentation OR “validation studies” OR “comparative study” OR psychometrics OR psychometr* OR clinimetr* OR clinometr* OR “outcome assessment” OR “outcome assessment” OR “outcome measure*” OR “observer variation” OR “observer variation” OR “health status indicators” OR “reproducibility of results” OR reproducib* OR “discriminant analysis” OR reliab* OR unreliab* OR valid* OR coefficient OR homogeneity OR homogeneous OR “internal consistency” OR cronbach* OR alpha OR alphas OR item OR selection* OR reduction* OR agreement OR precision OR imprecision OR “precise values” OR testeretest OR retest OR reliab* OR retest OR stability OR interrater OR “inter-rater” OR intrarater OR intrarater OR intertester OR “inter-tester” OR intratester OR “intra-tester” OR interobserver OR “inter-observer” OR intraobserver OR intraobserver OR interexaminer OR interexaminer OR intraexaminer OR “intra-examiner” OR interassay OR “inter-assay” OR intraassay OR “intra-assay” OR interindividual OR “inter-individual” OR intraindividual OR “intra-individual” OR interparticipant OR “inter-participant” OR intraparticipant OR “intra-participant” OR kappa* OR repeatab* OR replicab* OR repeated OR concordance OR intraclass OR discriminative OR “known group” OR “factor analysis” OR “factor analyses” OR dimension* OR subscale* OR multitrait OR scaling OR “item discriminant” OR “interscale correlation*” OR error OR errors OR “individual variability” OR variability OR measurement OR measuring OR “standard error of measurement” OR sensitiv* OR responsive* OR detectable OR “meaningful change” OR “ceiling effect” OR “floor effect” OR “item response model” OR IRT OR rasch OR “differential item functioning” OR DIF OR “computer adaptive testing” OR “item bank” OR “crosscultural equivalence”):ab,ti,de

**Supplementary Material S2.** References of included studies.

Andrade-González, N., Lahera, G., & Fernández-Liria, A. (2016). Patient-Therapist Perspective of the Working Alliance in Psychotherapy. *Psychiatric Quarterly*, *88*(3), 623–633. https://doi.org/10.1007/s11126-016-9477-4 LK

Andrade-González, Nelson, & Fernández-Liria, A. (2015). Spanish Adaptation of the Working Alliance Inventory (WAI). Psychometric properties of the patient and therapist forms (WAI-P and WAI-T). *Anales de Psicología*, *31*(2), 524–533. https://doi.org/10.6018/analesps.31.2.177961

Andrusyna, T. P., Tang, T. Z., DeRubeis, R. J., & Luborsky, L. (2001). The factor structure of the Working Alliance Inventory in cognitive-behavioral therapy. *The Journal of Psychotherapy Practice and Research*, *10*(3), 173–178.

Araujo, A. C., Oliveira, C. B., Ferreira, P. H., & Pinto, R. Z. (2017). Measurement properties of the Brazilian version of the Working Alliance Inventory (patient and therapist short-forms) and Session Rating Scale for low back pain. *Journal of Back and Musculoskeletal Rehabilitation*, *30*(4), 879–887. https://doi.org/10.3233/BMR-160563

Bat Or, M., & Zilcha-Mano, S. (2018). The art therapy working alliance inventory: The development of a measure. *International Journal of Art Therapy*, *24*(2), 76-87. https://doi.org/10.1080/17454832.2018.1518989

Bedregal, L. E., Paris, M., Añez, L. M., Shahar, G., & Davidson, L. (2006). Preliminary Evaluation of the Validity and Reliability of the Spanish Version of the Therapeutic Alliance with Clinician (TAC) Questionnaire. *Social Indicators Research*, *78*(1), 19–32. https://doi.org/10.1037/h0085885

Busseri, M. A., & Tyler, J. D. (2003). Interchangeability of the Working Alliance Inventory and Working Alliance Inventory, Short Form. *Psychological Assessment*, *15*(2), 193–197. https://doi.org/10.1037/1040-3590.15.2.193

Cecero, J. J., Fenton, L. R., Frankforter, T. L., Nich, C., & Carroll, K. M. (2001). Focus on therapeutic alliance: The psychometric properties of six measures across three treatments. *Psychotherapy: Theory, Research, Practice, Training*, *38*(1), 1–11. https://doi.org/10.1037/0033-3204.38.1.1

Chen, R. M., Chen, J. H., & Xu, Y. F. (2018). Reliability and validity of Chinese version of Working Alliance Inventory-Short Form Revised in the inpatients with schizophrenia. *Journal of Shanghai Jiaotong University (Medical Science)*, *38*(12), 1494–1498. https://doi.org/10.3969/j.issn.1674-8115.2018.12.018

Cirasola, A., Midgley, N., Fonagy, P., & Martin, P. (2021). The factor structure of the Working Alliance Inventory short-form in youth psychotherapy: an empirical investigation. *Psychotherapy Research : Journal of the Society for Psychotherapy Research*, *31*(4), 535–547. https://doi.org/10.1080/10503307.2020.1765041

Corbella, S., & Botella, L. (2004). Psychometric properties of the Spanish version of the Working Alliance Theory of Change Inventory (WATOCI). *Psicothema*, *16*(4), 702–705.

Corbella, S., Botella, L., Gómez, A. M., Herrero, O., & Pacheco, M. (2011). Características psicométricas de la versión española del Working Alliance Inventory-Short (WAI-S). *Anales de Psicología/Annals of Psychology*, *27*(2), 298–301.

Corbière, M., Bisson, J., Lauzon, S., & Ricard, N. (2006). Factorial validation of a French short-form of the Working Alliance Inventory. *International Journal of Methods in Psychiatric Research*, *15*(1), 36–45. https://doi.org/10.1002/mpr.27

Ely, B., Alexander, L. B., & Reed, M. (2005). The Working Alliance in Pediatric Chronic Disease Management: A Pilot Study of Instrument Reliability and Feasibility. *Journal of Pediatric Nursing*, *20*(3), 190–200. https://doi.org/10.1016/j.pedn.2005.01.007

Falkenström, F., Hatcher, R. L., & Holmqvist, R. (2015). Confirmatory Factor Analysis of the Patient Version of the Working Alliance Inventory–Short Form Revised. *Assessment*, *22*(5), 581–593. https://doi.org/10.1177/1073191114552472

Falkenström, F., Hatcher, R. L., Skjulsvik, T., Larsson, M. H., & Holmqvist, R. (2015). Development and validation of a 6-item working alliance questionnaire for repeated administrations during psychotherapy. *Psychological Assessment*, *27*(1), 169–183. https://doi.org/10.1037/pas0000038

Fenton, L. R., Cecero, J. J., Nich, C., Frankforter, T. L., & Carroll, K. M. (2001). Perspective is everything: The predictive validity of six working alliance instruments. *The Journal of Psychotherapy Practice and Research*, *10*(4), 262–268.

Figueiredo, B., Dias, P., Lima, V. S., & Lamela, D. (2016). Working Alliance Inventory for Children and Adolescents (WAI-CA). *European Journal of Psychological Assessment*, *35*(1), 22–28. https://doi.org/10.1027/1015-5759/a000364

Goldberg, R. W., Rollins, A. L., & McNary, S. W. (2004). The Working Alliance Inventory: Modification and use with people with serious mental illnesses in a vocational rehabilitation program. *Psychiatric Rehabilitation Journal*, *27*(3), 267–270. https://doi.org/10.2975/27.2004.267.270

Guédeney, N., Fermanian, J., Curt, F., & Bifulco, A. (2005). Testing the Working Alliance Inventory (WAI) in a French primary care setting. *Social Psychiatry and Psychiatric Epidemiology*, *40*(10), 844–852. https://doi.org/10.1007/s00127-005-0972-4

Gulum, I. V., Uluc, S., & Soygut, G. (2018). Psychometric Properties of the Turkish Working Alliance Inventory- Short Form. *Turkish Journal of Psychiatry*, *29*(1), 47–53. https://doi.org/10.5080/u18260

Hall, A. M., Ferreira, M. L., Clemson, L., Ferreira, P., Latimer, J., & Maher, C. G. (2012). Assessment of the therapeutic alliance in physical rehabilitation: a RASCH analysis. *Disability and Rehabilitation*, *34*(3), 257–266. https://doi.org/10.3109/09638288.2011.606344

Hatcher, R. (1999). Therapists’ views of treatment alliance and collaboration in therapy. *Psychotherapy Research*, *9*(4), 405–423.

Hatcher, R. L., Barends, A., Hansell, J., & Gutfreund, M. J. (1995). Patients’ and therapists’ shared and unique views of the therapeutic alliance: An investigation using confirmatory factor analysis in a nested design. *Journal of Consulting and Clinical Psychology*, *63*(4), 636–643. https://doi.org/10.1037/0022-006X.63.4.636

Hatcher, R. L., & Barends, A. W. (1996). Patients’ view of the alliance in psychotherapy: Exploratory factor analysis of three alliance measures. *Journal of Consulting and Clinical Psychology*, *64*(6), 1326–1336. https://doi.org/10.1037/0022-006X.64.6.1326

Hatcher, R. L., & Gillaspy, J. A. (2006). Development and validation of a revised short version of the working alliance inventory. *Psychotherapy Research*, *16*(1), 12–25. https://doi.org/10.1080/10503300500352500

Hatcher, R. L., Lindqvist, K., & Falkenström, F. (2020). Psychometric evaluation of the Working Alliance Inventory-Therapist version: Current and new short forms. *Psychotherapy Research : Journal of the Society for Psychotherapy Research*, *30*(6), 706–717. https://doi.org/10.1080/10503307.2019.1677964

Herrero, R., Vara, M. D., Miragall, M., Botella, C., García-Palacios, A., Riper, H., Kleiboer, A., & Baños, R. M. (2020). Working Alliance Inventory for Online Interventions-Short Form (WAI-TECH-SF): The Role of the Therapeutic Alliance between Patient and Online Program in Therapeutic Outcomes. *International Journal of Environmental Research and Public Health*, *17*(17). 6169. https://doi.org/10.3390/ijerph17176169

Horvath, A. O., & Greenberg, L. S. (1989). Development and validation of the Working Alliance Inventory. *Journal of Counseling Psychology*, *36*(2), 223–233. https://doi.org/10.1037/0022-0167.36.2.223

Hsu, S, Zhou, R. H., & Yu, C. K. C. (2016). A Hong Kong validation of working alliance inventory – short form – client. *Asia Pacific Journal of Counselling and Psychotherapy*, *7*(1–2), 69–81. https://doi.org/10.1080/21507686.2016.1193036

Hsu, Sharon, & Yu, C. K.-C. (2017). A Hong Kong study of working alliance inventory short form – therapist. *Asia Pacific Journal of Counselling and Psychotherapy*, *8*(2), 87–100. https://doi.org/10.1080/21507686.2017.1313285

Hukkelberg, S., & Ogden, T. (2017). General and specific factors of working alliance in parent training: A bifactor exploratory structural equation modelling approach. *Psychotherapy Research*, *29*(2), 267–276. https://doi.org/10.1080/10503307.2017.1330574

Hukkelberg, S. S., & Ogden, T. (2016). The short Working Alliance Inventory in parent training: Factor structure and longitudinal invariance. *Psychotherapy Research*, *26*(6), 719–726. https://doi.org/10.1080/10503307.2015.1119328

Hunik, L., Galvin, S., Olde Hartman, T., Rieger, E., Lucassen, P., Douglas, K., Boeckxstaens, P., & Sturgiss, E. (2021). Exploring the psychometric properties of the Working Alliance Inventory in general practice: a cross-sectional study. *BJGP Open*, *5*(1), bjgpopen20X101131. https://doi.org/10.3399/bjgpopen20X101131

Karel, Y., Thoomes-de Graaf, M., Scholten-Peeters, G., Ferreira, P., Rizopoulos, D., Koes, B. W., & Verhagen, A. P. (2018). Validity of the Flemish working alliance inventory in a Dutch physiotherapy setting in patients with shoulder pain. *Physiotherapy Theory and Practice*, *34*(5), 384–392. https://doi.org/10.1080/09593985.2017.1400141

Killian, M., Forrester, D., Westlake, D., & Antonopoulou, P. (2017). Validity of the Working Alliance Inventory Within Child Protection Services. *Research on Social Work Practice*, *27*(6), 704–715. https://doi.org/10.1177/1049731515596816

Knowles, C., Murray, C., Gau, J., & Toste, J. R. (2020). Teacher–student working alliance among students with emotional and behavioral disorders. *Journal of Psychoeducational Assessment*, *38*(6), 753–761. https://doi.org/10.1177/0734282919874268

Lamers, A., Delsing, M. J. M. H., van Widenfelt, B. M., & Vermeiren, R. R. J. M. (2015). A Measure of the Parent-Team Alliance in Youth Residential Psychiatry: The Revised Short Working Alliance Inventory. *Child & Youth Care Forum*, *44*(6), 801–817. https://doi.org/10.1007/s10566-015-9306-1

Mallinckrodt, B., & Tekie, Y. T. (2016). Item response theory analysis of Working Alliance Inventory, revised response format, and new Brief Alliance Inventory. *Psychotherapy Research*, *26*(6), 694–718. https://doi.org/10.1080/10503307.2015.1061718

Miloff, A., Carlbring, P., Hamilton, W., Andersson, G., Reuterskiöld, L., & Lindner, P. (2020). Measuring alliance toward embodied virtual therapists in the era of automated treatments with the Virtual Therapist Alliance Scale (VTAS): Development and psychometric evaluation. *Journal of Medical Internet Research*, *22*(3), e16660. https://doi.org/10.2196/16660

Milot-Lapointe, F., Le Corff, Y., & Savard, R. (2020). Factor structure of the short version of the Working Alliance Inventory and its longitudinal measurement invariance across individual career counseling sessions. *Journal of Career Assessment*, *28*(4), 693–705. https://doi.org/10.1177/1069072720925048

Miragall, M., Baños, R. M., Cebolla, A., & Botella, C. (2015). Working alliance inventory applied to virtual and augmented reality (WAI-VAR): psychometrics and therapeutic outcomes. *Frontiers in Psychology*, *6*, 1531. https://doi.org/10.3389/fpsyg.2015.01531

Munder, T., Wilmers, F., Leonhart, R., Linster, H. W., & Barth, J. (2009). Working Alliance Inventory-Short Revised (WAI-SR): psychometric properties in outpatients and inpatients. *Clinical Psychology & Psychotherapy*, *17*(3), 231–239. https://doi.org/10.1002/cpp.658

Paap, D., Schepers, M., & Dijkstra, P. U. (2019). Reducing ceiling effects in the Working Alliance Inventory-Rehabilitation Dutch Version. *Disability and Rehabilitation*, *42*(20), 2944–2950. https://doi.org/10.1080/09638288.2018.1563833

Paap, D., Schrier, E., & Dijkstra, P. U. (2018). Development and validation of the Working Alliance Inventory Dutch version for use in rehabilitation setting. *Physiotherapy Theory and Practice*, *35*(12), 1292–1303. https://doi.org/10.1080/09593985.2018.1471112

Penedo, J. M. G., Berger, T., Holtforth, M. grosse, Krieger, T., Schröder, J., Hohagen, F., Meyer, B., Moritz, S., & Klein, J. P. (2019). The Working Alliance Inventory for guided Internet interventions (WAI‐I). *Journal of Clinical Psychology*, *76*(6), 973-986 https://doi.org/10.1002/jclp.22823

Perdrix, S., de Roten, Y., Kolly, S., & Rossier, J. (2010). The Psychometric Properties of the WAI in a Career Counseling Setting: Comparison With a Personal Counseling Sample. *Journal of Career Assessment*, *18*(4), 409–419. https://doi.org/10.1177/1069072710374583

Petek, D., Pusnik, A., Selic, P., Cedilnik-Gorup, E., Trontelj, Z., Riou, M., & Yves Le Reste, J. (2019). Semantic and Cultural Equivalence of the Working Alliance Inventory Short-revised Scale for Therapeutic Alliance in Family Medicine: Lessons Learned in Slovenia. *Zdravstveno Varstvo*, *58*(1), 21–30. https://doi.org/10.2478/sjph-2019-0003

Prusiński, T. (2021). Patients’ and psychotherapists’ combined and separate evaluations of the psychotherapeutic relationship: The structure of working alliance and polish versions of the wai. *Journal of Contemporary Psychotherapy: On the Cutting Edge of Modern Developments in Psychotherapy*. https://doi.org/10.1007/s10879-021-09500-z

Ross, E. C., Polaschek, D. L. L., & Wilson, M. (2011). Shifting perspectives: a confirmatory factor analysis of the working alliance inventory (short form) with high-risk violent offenders. *International Journal of Offender Therapy and Comparative Criminology*, *55*(8), 1308–1323.

Santibáñez, P. (2003). La Alianza Terapéutica en Psicoterapia: El" Inventario de Alianza de Trabajo" en Chile. *Psykhe*, *12*(1), 109–118.

Santirso, F. A., Martín-Fernández, M., Lila, M., Gracia, E., & Terreros, E. (2018). Validation of the Working Alliance Inventory–Observer Short Version with male intimate partner violence offenders. *International Journal of Clinical and Health Psychology*, *18*(2), 152–161. https://doi.org/10.1016/j.ijchp.2018.02.003

Smits, D., Luyckx, K., Smits, D., Stinckens, N., & Claes, L. (2015). Structural characteristics and external correlates of the Working Alliance Inventory-Short Form. *Psychological Assessment*, *27*(2), 545–551. https://doi.org/10.1037/pas0000066

Soygüt, G., & Işikli, S. (2008). Assessment of the Therapeutic Alliance: Reliability and Validity of the Working Alliance Inventory. *Turkish Journal of Psychiatry*, *19*(4), 398–408.

Soygüt, G., & Uluc, S. (2009). Psychometric properties of the Turkish working alliance inventory-observer form. *Türk Psikiyatri Dergisi*, *20*(4), 367–375.

Stiles, W. B., Agnew-Davies, R., Barkham, M., Culverwell, A., Goldfried, M. R., Halstead, J., Hardy, G. E., Raue, P. J., Rees, A., & Shapiro, D. A. (2002). Convergent validy of the Agnew Relationship Measure and the Working Alliance Inventory. *Psychological Assessment*, *14*(2), 209–220. https://doi.org/10.1037/1040-3590.14.2.209

Stinckens, N., Ulburghs, A., & Claes, L. (2009). De werkalliantie als sleutelelement in het therapiegebeuren. *Meting Met Behulp van de WAV-12: De Nederlandse Vertaling van de Working Alliance Inventory. Tijdschrift Klininische Psychologie*, *39*, 44–60.

Takasaki, H., Miki, T., & Hall, T. (2019). Development of the Working Alliance Inventory-Short Form Japanese version through factor analysis and test–retest reliability. *Physiotherapy Theory and Practice*, *36*(3), 444–449. https://doi.org/10.1080/09593985.2018.1487492

Tatman, A. W., & Love, K. M. (2010). An Offender Version of the Working Alliance Inventory-Short Revised. *Journal of Offender Rehabilitation*, *49*(3), 165–179. https://doi.org/10.1080/10509671003666560

Tichenor, V., & Hill, C. E. (1989). A comparison of six measures of working alliance. *Psychotherapy: Theory, Research, Practice, Training*, *26*(2), 195–199. https://doi.org/10.1037/h0085419

Toste, J. R., Heath, N. L., McDonald Connor, C., & Peng, P. (2015). Reconceptualizing teacher-student relationships: Applicability of the working alliance within classroom contexts. *The Elementary School Journal*, *116*(1), 30–48. https://doi.org/https://doi.org/10.1086/683110

Tracey, T. J., & Kokotovic, A. M. (1989). Factor structure of the Working Alliance Inventory. *Psychological Assessment: A Journal of Consulting and Clinical Psychology*, *1*(3), 207–210. https://doi.org/10.1037/1040-3590.1.3.207

Vöhringer, C., Pérez, J. C., Martínez, C., Altimir, C., Dagnino, P., Suárez, N., & Krause, M. (2013). “ Working Alliance Inventory” Versión Observacional: Traducción, Adaptación y Validación al Castellano. *Terapia PsicolÓgica*, *31*(3), 301–311.

Warlick, C., Richter, K. P., Catley, D., Gajewski, B. J., Martin, L. E., & Mussulman, L. M. (2019). Two brief valid measures of therapeutic alliance in counseling for tobacco dependence. *Journal of Substance Abuse Treatment*, *86*, 60–64. https://doi.org/10.1016/j.jsat.2017.12.010

Wilmers, F., Munder, T., Leonhart, R., Herzog, T., Plassmann, R., Barth, J., & Linster, H. W. (2008). Die deutschsprachige Version des Working Alliance Inventory-short revised (WAI-SR)-Ein schulenübergreifendes, ökonomisches und empirisch validiertes Instrument zur Erfassung der therapeutischen Allianz. *Klinische Diagnostik Und Evaluation*, *1*(3), 343–358.

**Table S1.** Characteristics of the studies included in this systematic review (in total n=66).

| **Author**  **(publ. year, ascending)** | **WAI-Version, Scales, number items** | **Country/ Language** | **Study Design** | **Measure-ment Properties** | **Type of Likert Scale (anchors)/ Scoring range** | **Mean scores,**  **(**$\boldsymbol{\pm}$**SD)*#** | **Setting, Target population, Treatment** | ***n*** | **Female %** | **Age Years**  **Mean, (SD)*** |
| --- | --- | --- | --- | --- | --- | --- | --- | --- | --- | --- |
| Horvath (1989) | WAI-P  Bond, 12  Goal, 12  Task, 12 | Canada/  English | Pilot  Cross/  Long | Develop  Cont V  Int C  Constr V | 7-point scale (1=never and 7=always)/  1-7 | Development/  Pilot study -- (--) | Counseling psychology; university students; following the counseling psychology program | P 29 | -- | -- (--) |
|  |  |  |  |  |  | Study 1  -- (--) | Psychology; client-counseling dyads; range of theoretical orientations | P 29  T 29 | -- | -- (--) |
|  | WAI-T  Bond, 12  Goal, 12  Task, 12 |  |  |  |  | Study 2  -- (--) | Psychology; clients; following gestalt two- chair technique therapy | P 31 | 89 % | -- (--) |
|  |  |  |  |  |  | Study 3  -- (--) | Psychology; client-counseling dyads; range of theoretical orientations | P 25  T 25 | -- | -- (--) |
| Tichenor (1989) | WAI-P  Bond, 12  Goal, 12  Task, 12 | USA/  English | Long | Cont V  Int C  Reliab  Constr V | 7-point scale (1=never and 7=always)/  1-7 | -- (--) | Psychotherapy; clients with depression and anxiety problems; treated with different theoretical orientations | P 8 | 100 % | 42.4 (9.4) |
|  | WAI-T  Bond, 12  Goal, 12  Task, 12 |  |  |  |  | -- (--) |  | T 8 | 50 % | 46.4 (13.5) |
|  | WAI-O  Bond, 12  Goal, 12  Task, 12 |  |  |  |  | -- (--) |  | O 6 | 67 % | -- (--) |
| Tracey (1989) | WAI-P  Bond, 12  Goal, 12  Task, 12 | USA/  English | Cross | Struct V | 7-point scale (1=never and 7=always)/  1-7 | -- (--) | Psychotherapy; clients; treated with psychodynamic, humanistic, and CBT therapy | P 84 | 63 % | 22.0 (--) |
|  | WAI-T  Bond, 12  Goal, 12  Task, 12 |  |  |  |  | -- (--) |  | T 15 | 47 % | -- (--) |
| Hatcher (1995) | WAI-P  Bond, 12  Goal, 12  Task, 12 | USA/  English | Cross | Struct V  Constr V | 7-point scale (1=never and 7=always)/  1-7 | -- (--) | Psychotherapy; outpatients with neurotic disturbances and personality disorders; treated with psychodynamic psychotherapy | P 144 | 69 % | Mdn 26 (--) |
|  | WAI-T  Bond, 12  Goal, 12  Task, 12 |  |  |  |  | -- (--) |  | T 38 | 63 % | -- (--) |
| Hatcher (1996) | WAI-P  Bond, 12  Goal, 12  Task, 12 | USA/  English | Cross | Struct V  Constr V | 7-point scale (1=never and 7=always)/  1-7 | -- (--) | Psychotherapy; outpatients with depression, anxiety, and relationship problems; treated with psychodynamic psychotherapy | P 231  T 64 | 64 %  68% | Mdn 27  (--)  -- (--) |
| Hatcher (1999) | WAI-T  Bond, 12  Goal, 12  Task, 12 | USA/  English | Cross | Struct V  Int C  Constr V | 7-point scale (1=never and 7=always)/  1-7 | Sample 1  Total (5.8) | Psychotherapy; patients with a variety of diagnoses; treated with psychodynamic/CBT and eclectic/other therapies | P 251  T 251 | 72%  50% | 40.8 (--)  --(--) |
|  |  |  |  |  |  | Sample 2  Total 4.9) | Psychotherapy; patients with affective and anxiety disorders; treated with psychodynamic therapy | P 259  T 63 | 66%  70% | 28.2 (--)  --(--) |
| Andrusyna (2001) | WAI-S-O  Bond, 4  Goal, 4  Task, 4 | USA/ English | Cross | Struct V | 7-point scale (1=never and 7=always)/  1-7 | -- (--) | Psychology; outpatients with depression; treated with CBT | P 94  T 4  O 2 | 76 %  --  -- | 39.0 (--)  44.0 (--)  -- (--) |
| Cecero (2001) | WAI-O  Bond, 12  Goal, 12  Task, 12 | USA/ English | Long | Int C  Reliab  Constr V | 7-point scale (1=never and 7=always)/  1-7 | Bond 4.2 (1.1)  Goal 4.3 (1.0)  Task 4.4 (1.1)  Total 4.3 (1.0) | Psychology; patients with addiction problems; treated with CBT, TSF, or clinical management | O 6 | 83% | -- (--) |
|  | WAI-P  Bond, 12  Goal, 12  Task, 12 |  |  |  |  | Bond 5.7 (1.0)  Goal 5.7 (0.8)  Task 5.8 (0.9)  Total 5.7 (0.8) |  | P 60 | 25 % | -- (--) |
|  | WAI-T  Bond, 12  Goal, 12  Task, 12 |  |  |  |  | Bond 5.1 (0.5) Goal 5.1 (0.7)  Task 5.2 (0.6) Total 5.1 (0.6) |  | T 11 | 18 % | -- (--) |
| Fenton (2001) | WAI-O  Bond, 12  Goal, 12  Task, 12 | USA/ English | Long | Reliab  Constr V | 7-point scale (1=never and 7=always)/  1-7 | Total 4.4 (0.1) | Psychology; patients with cocaine and/or alcohol dependency; treated with CBT, TSF, or clinical management therapy | O 6 | 83% | -- (--) |
|  | WAI-P  Bond, 12  Goal, 12  Task, 12 |  |  |  |  | Total 5.8 (0.9) |  | P 46 | -- | -- (--) |
|  | WAI-T  Bond, 12  Goal, 12  Task, 12 |  |  |  |  | Total 5.1 (0.6) |  | T 6 | -- | -- (--) |
| Stiles (2002) | Study 1  WAI-P  Bond, 12  Goal, 12  Task, 12 | USA/  English | Long | Constr V | 7-point scale (1=never and 7=always)/  1-7 | Bond 5.8 (1.0)  Goal 5.5 (1.0)  Task 5.7 (0.9)  Total 5.7 (1.0) | Psychology; clients with depression; treated with psychodynamic-interpersonal therapy and CBT | P 18 | 61 % | 39.0 (--) |
|  | WAI-T  Bond, 12  Goal, 12  Task, 12 |  |  |  |  | Bond 5.8 (0.7)  Goal 5.3 (0.9)  Task 5.5 (0.9) Total 5.5 (0.8) |  | T 4 | 75% | -- (--) |
|  | Study 2  WAI-O  Bond, 12  Goal, 12  Task, 12 | USA/  English | Long | Constr V | 7-point scale (1=never and 7=always)/  1-7 | Bond 6.1 (0.5)  Goal 6.1 (0.5)  Task 6.0 (0.5)  Total 6.1 (0.5) | Psychology; clients with depression; treated with psychodynamic-interpersonal therapy and CBT | P 39  T 5  O 6 | 64 %  40 %  -- | 41.0 (--)  -- (--)  -- (--) |
| Busseri (2003) | WAI-P  Bond, 12  Goal, 12  Task, 12 | USA/ English | Long | Int C  Constr V | 7-point scale (1=never and 7=always)/  1-7 | Bond 5.9 (0.8)  Goal 5.8 (0.9)  Task 5.9 (0.8)  Total 5.9 (0.8) | Psychology; university students; treated for psychological problems; university counseling therapy | P 54 | -- | -- (--) |
|  | WAI-T  Bond, 12  Goal, 12  Task, 12 |  |  |  |  | Bond 5.8 (0.5)  Goal 5.3 (0.8)  Task 5.4 (0.7)  Total 5.5 (0.5) |  | T 18 | -- | -- (--) |
|  | WAI-S-P  Bond, 4  Goal, 4  Task, 4 |  |  |  |  | Bond 5.9 (1.0) Goal 5.9 (1.0)  Task 5.8 (1.0) Total 5.9 (0.9) |  | P 54 | -- | -- (--) |
|  | WAI-S-T  Bond, 4  Goal, 4  Task, 4 |  |  |  |  | Bond 5.8 (0.6) Goal 5.4 (0.8) Task 5.2 (1.0) Total 5.5 (0.8) |  | T 18 | -- | -- (--) |
| Santibánez (2003) | IAT-S-P  Bond, 4  Goal, 4  Task, 4 | Chile/  Spanish | Cross | Struct V  Int C  Constr V | 7-point scale (1=never and 7=always)/  4-84 | -- (--) | Psychotherapy; patients with different problems; treated with different therapies | P 72 | 72 % | -- (--) |
|  | IAT-S-T  Bond, 4  Goal, 4  Task, 4 |  |  |  |  | -- (--) |  | T 45 | 83 % | -- (--) |
| Corbella (2004) | WATOCI  Bond, 4  Goal, 4  Task, 4  Theory of change, 5 | Spain/ Spanish | Cross | Struct V  Int C | 7-point scale (1=never and 7=always)/  4-119 | -- (--) | Psychotherapy; outpatients with anxiety, depression, interpersonal/ relational difficulties; receiving outpatient psychotherapy | P 102 | 77 % | 30.0 (8.7) |
| Goldberg (2004) | WAI-S-P  Bond 4  Goal 4  Task 4 | USA/ English | Long | Reliab  Int C | 7-point scale (1=never and 7=always)/  4-84 | Bond 23.1 (4.6)  Goal 21.3 (3.7)  Task 20.9 (5.7)  Total 65.4 (12.3) | Psychiatric rehabilitation; patients with serious mental illness; two different approaches of vocational rehabilitation | P 64 | 41 % | 40.7 (8.5) |
|  | WAI-S-T  Bond, 4  Goal, 4  Task, 4 |  |  |  |  | Bond 21.8 (2.8) Goal 17.0 (4.8) Task 17.9 (4.2) Total 55.6 (11.0) |  | T 4 | -- | -- (--) |
| Ely (2005) | WAICC  10 different versions for  adolescents, 12 | USA/ English | Adapt/  Long | Int C  Reliab | 7-point scale (1=never and 7=always)/  36-252 | Total 236.1 (10.3) § | Chronic childhood hematologic disorders; patients with hematologic disorders, parents/guardians and healthcare providers; medically focused care | P 47 | 56 % | 13.9 (1.9) |
|  | WAICC  10 different versions for children, 36 |  |  |  | 5-point scale (1=never and 5=always)/ 12-60 | Total 53.3  (4.3) § |  | Par 43  T 4 | 97 %  50 % | -- (--)  -- (--) |
| Guédeney (2005) | WAI-P & WAI-T  Positive expectations  of the usefulness, 21    Absence of suspicion about the effects of help, 13 | France/  French | Transl/  Long | Struct V  Constr V | 7-point scale (1=never and 7=always)/ 36-252 | Total 173.2 (37.7) | Social work; clients with social dysfunction, anxiety, somatic disorders and depression; social work therapy | P 130 | 78 % | 31.8 (6.6) |
| Bedregal (2006) | TAC  Total, 9 | USA/ Spanish | Transl /  Cross | Cont V  Struct V  Int C  Constr V | Items [1 to 8] 7-point scale (1=never and 7= always)\| item [9] 6-point scale (1=extremely satisfied and 6=extremely dissatisfied)/ 1-7 | Total 6.5 (0.8) | Psychology; clients with a Hispanic ethnicity; treated with behavioral treatment | P 102 | 100 % | 43.7 (13.2) |
| Corbiѐre (2006) | WAI-S-P  Bond, 4  Goal, 4  Task, 4 | Canada/ French | Transl/  Cross | Cont V  Struct V | 7-point scale (1=never and 7=always)/  1-7 | Bond 6.2 (0.8) Goal 6.0 (0.9)  Task 5.8 (0.8) Total 5.9 (0.8) | Psychotherapy; clients with psychiatric problems; intensive multidisciplinary community program | P 150 | 73 % | 51.7 (14.0) |
|  | WAI-S -T  Bond, 4  Goal, 4  Task, 4 |  |  |  |  | Bond 6.2 (0.7) Goal 5.7 (0.9) Task 5.8 (0.9) Total 5.9 (0.8) |  | T 30 | 73 % | 44.5 (6.2) |
| Hatcher (2006) | WAI-SR-P  Bond, 4  Goal, 4  Task, 4 | USA/ English | Cross | Struct V  Intl C  Constr V  Mea Inv | 7-point scale (1=never and 7=always)/  1-7 | Sample 1  Bond 5.1 (1.0) Goal 4.8 (1.0) Task 5.1 (0.9) Total 5.2 (0.9) | Psychotherapy; clients with depression, anxiety and relationship problems; treated with psychodynamic psychotherapy | P 231 | 64 % | 28.5 (7.2) |
|  |  |  |  |  |  | Sample 2  Bond 5.9 (0.8) Goal 5.8 (0.8) Task 5.8 (0.8) Total 5.9 (0.8) | Psychotherapy; counseling and outpatients; mostly treated with psychodynamic psychotherapy and CBT | P 235 | 71 % | 28.4 (9.9) |
| Soygüt (2008) | WAI-P  Bond, 8  Goal, 8  Task, 8 | Turkey/ Turkish | Transl /  Cross | Cont V  Struct V  Int C  Constr V | 7-point scale (1=never and 7=always)/  1-7 | -- (--) | Psychotherapy; patients with various problem areas; treated with different theoretical approaches | P 63 | 60 % | 29.7 (8.9) |
|  | WAI-T  Bond, 8  Goal, 8  Task, 8 |  |  |  |  | -- (--) |  | T 21 | 76 % | -- (--) |
| Wilmers (2008) | WAI-SR-P  Bond, 4  Goal, 4  Task, 4 | Germany/  German | Transl/  Cross | Cont V  Struct V  Constr V | 5-point scale (1=never and 5=always)/  1-5 | -- (--) | Psychotherapy; in- and outpatients with depression and anxiety disorders; treated with integrative psychodynamic therapy and CBT | P 331 | 66 % | 36.9 (12.0) |
| Soygüt (2009) | WAI-O  Bond, 8  Goal, 8  Task, 8 | Turkey/  Turkish | Transl  / Cross | Cont V  Structl V  Int C  Reliab | 7-point scale (1=never and 7=always)/  1-7 | -- (--) | Psychotherapy, outpatients with depression and anxiety disorders; treated with CBT | O 3  P 10  T 6 | --  --  -- | -- (--)  -- (--)  -- (--) |
| Stinckens (2009) | WAV-12-P  Bond, 4  Goal, 4  Task, 4 | Belgium/ Flemish | Cross | Struct V  Int C  Constr V | 5-point scale (1=never and 5=always)/  1-5 | Bond 4.0 (0.9)  Goal 3.6 (0.9)  Task 3.7 (0.8)  Total 3.8 (0.8) | Psychotherapy in different settings; outpatients with Axis-I and Axis-II disorders; treated with different approaches | P 256  T 37 | 64 %  68 % | 34.5 (12.4)  32.5 (8.1) |
| Munder (2009) | WAI-SR-P  Bond, 4  Goal, 4  Task, 4 | Germany/ German | Cross | Struct V  Int C  Constr V  Meas Inv | 5-point scale (1=never and 5=always)/  1-5 | Sample 1  Bond 4.0 (0.8)  Goal 4.0 (0.7)  Task 3.4 (0.8)  Total 3.8 (0.8) | Psychotherapy; outpatients with depression, anxiety, adjustment and personality disorders; treated with CBT | P 88 | 63 % | 35.2 (11.4) |
|  |  |  |  |  |  | Sample 2  Bond 3.7 (0.9)  Goal 3.6 (1.0)  Task 3.4 (0.9)  Total 3.6 (0.8) | Psychotherapy; inpatients with depression, eating, adjustment and personality disorders; treated with different types of therapies | P 243 | 75 % | 38.6 (12.4) |
| Perdrix (2010) | WAI-SR-P  Bond, 4  Goal, 4  Task, 4 | Switzerland, French | Transl/ Cross | Cont V  Struct V  Int C | 7-point scale (1=never and 7=always)/  1-7 | Sample 1  Bond 5.6 (0.6)  Goal 5.8 (0.8)  Task 5.5 (0.8)  Total 5.6 (0.8) | Career counseling; school pupils and college or university students; counseling | P 188 | 49 % | 21.4 (7.1) |
|  |  |  |  |  |  | Sample 2  Bond 5.6 (0.9)  Goal 5.6 (0.9)  Task 5.4 (0.9)  Total 5.5 (0.9) | Personal counseling; university students with personal problems in a counseling clinic; counseling | P 95 | 65 % | 23.9 (3.2) |
| Tatman (2010) | WAI-SR-P  Bond, 4  Goal, 4  Task, 4 | USA/ English | Long | Struct V  Int C  Reliab  Constr V | 7-point scale (1=never and 7=always)/  4-84 | Sample1  Bond 23.4 (5.9)  Goal 23.5 (5.0)  Task 23.1 (5.9)  Total 67.0 (15.0) | Offender therapy; offenders on probation or parole for sexual offences or domestic abuse; participating in either court-ordered sex offender treatment or batterers’ education group treatment | P 182 | 0 % | 36.3 (12.2) |
|  |  |  |  |  |  | Sample 2  Total 70.0 (15.0) | idem | P 174 | 0 % | 30.3 (6.3) |
|  |  |  |  |  |  | Sample 3  Total 70.8 (12.2) | idem | P 202 | 0 % | -- (--) |
| Corbella (2011) | WAI-S-P  Bond, 4  Goal, 4  Task, 4 | Spain/ Spanish | Transl/  Cross | Cont V  Struct V  Int C | 7-point scale (1=never and 7=always)/  4-84 | -- (--) | Psychotherapy; outpatients with anxiety, depression, interpersonal/ relational difficulties; psychotherapy treatment | P 229  T 9 | 61 %  -- | 29.2 (8.8)  -- (--) |
| Ross (2011) | WAI-S-P  Bond, 12  Goal, 12  Task, 12 | New Zealand/  English | Cross | Struct V | 7-point scale (1=never and 7=always)/  1-7 | -- (--) | Offender therapy; prisoners nearing end of a prison sentence for a violent offense; participating in offender treatment | P 70  T 11  O 2 | 0%  27%  --(--) | 31 (--)    35 (--)  --(--) |
|  | WAI-S-T  Bond, 12  Goal, 12  Task, 12 |  |  |  |  |  |  |  |  |  |
|  | WAI-S-O  Bond, 12  Goal, 12  Task, 12 |  |  |  |  |  |  |  |  |  |
| Hall (2012) | WATOCI  Total, 16 | Australia/ English | Cross | Struct V  Int C  Constr V | 7-point scale (1=never and 7=always)/ 16-112 | Total 98.9 (13.2)** | Physiotherapy; patients with chronic low back pain; treated with exercise therapy and manual therapy | P 206 | 71 % | 53.5 (14.7) |
| Vöhringer (2013) | WAI-O  Bond, 12  Goal, 12  Task, 12 | Chile/ Spanish | Transl/ Cross | Con V  IntC  Constr V | 7-point scale (1=never and 7=always)/ 12-252 | Bond 54.6 (5.3)  Goal 51.9 (5.9)  Task 53.6 (5.9)  Total 160.1 (15.7) | Psychotherapy; patients with addiction problems and or depression; treated with different types of therapies | P 55  T 15  O -- | 53 %  74 %  -- | 36.4 (11.7)  -- (--)  -- (--) |
| Andrade-González (2015) | WAI-P  Bond, 12  Goal, 12  Task, 12 | Spain/ Spanish | Transl/  Pilot/  Long | Cont V  Int C  Constr V | 7-point scale (1=never and 7=always)/  1-7 | Bond 6.2 (0.8)  Goal 5.9 (1.0)  Task 5.9 (0.9)  Total 6.0 (0.9) | Psychology; outpatients with depression; most commonly used therapy was integrative therapy | Pilot study  P 10  T 10 | -- | -- (--) |
|  | WAI-T  Bond, 12  Goal, 12  Task, 12 |  |  |  |  | Bond 6.0 (0.6)  Goal 5.3 (0.9)  Task 5.4 (0.7)  Total 6.0 (0.7) |  | Clinical study  P 36  T 21 | 83 %    48 % | 42.4 (10.6)  35.0 (10.0) |
| Falkenström (2015)a | WAI-SR-P  Bond, 12  Goal, 12  Task, 12 | Sweden &USA/  Swedish & English | Cross | Struct V  Mea Inv | 7-point scale (1=never and 7=always)/  1-7 | Sample 1  Bond 6.0 (1.4) Goal 5.8 (1.3) Task 5.7 (1.2) Total 5.8 (1.3) | Psychology; outpatients; treated with different types of therapies (mostly CBT or psychodynamics) | P 235 | 71 % | 28.4 (9.9) |
|  |  |  |  |  |  | Sample 2  Bond 6.0 (1.1) Goal 5.8 (1.1) Task 5.5 (1.2) Total 5.8 (1.1) | Psychology; clients from primary care; treated with different types of therapies (mostly CBT or psychodynamics) | P 634 | 74 % | 37.3 (14.3) |
|  |  |  |  |  |  | Sample 3  Bond 5.9 (1.2) Goal 5.8 (1.3) Task 5.4 (1.3) Total 5.7 (1.3) | Psychology; clients from a specialist psychiatric department; treated with different types of therapies | P 234 | -- | -- (--) |
| Falkenström (2015)b | SAI-P  Bond, 3  Go/TA, 3 | Sweden &USA/  Swedish & English | Long | StructV  Int C  Constr V  Mea Inv | 7-point scale (1=never and 7=always)/  1-7 | Sample 1  Bond 6.0 (1.1) Go/Ta 5.8 (1.2)  Total 5.9 (1.2) | Psychology; patients in primary care; treatment mostly CBT or psychodynamics | P1006† | 74 % | 37.3 (14.3) |
|  |  |  |  |  |  | Sample 2  Bond 6.0 (1.1) Go/Ta 5.9 (1.2)  Total 6.0 (1.2) | Psychology; outpatients; treatment mostly CBT or psychodynamics | P 235 † | 71 % | 28.4 (9.9) |
|  |  |  |  |  |  | Sample 3  Bond 6.0 (1.2) Go/Ta 5.8 (1.2)  Total 5.9 (1.2) | Psychology; patients in a specialist psychiatric department; treated with different types of therapies | P234 † | -- | -- (--) |
| Lamers (2015) | WAV-12-P  Bond, 4  Goal, 4  Task, 4 | Netherlands/ Dutch | Transl/  Pilot /  Cross | Cont V  Struct V  Int C  Constr V | 7-point scale (1=never and 7=always)/  1-7 | Pilot study  -- (--) | Residential psychiatry; youth with case-managers and the primary caregivers as informants; receiving multidisciplinary treatment | P 20 | -- | -- (--) |
|  | WAV-12-Team  Bond, 4  Goal, 4  Task, 4 |  |  |  |  | -- (--) | Residential psychiatry; youth with autistic spectrum disorders and the primary caregivers (mostly mothers or two fathers); receiving multidisciplinary treatment | P 93 | 21 % | 10 (3) |
| Miragall (2015) | WAI-VAR-P  Bond, 4  Goal, 4  Task, 4 | Spain/ Spanish | Adapt/  Cross | Cont V  Struct V  Int C  Constr V | 7-point scale (1=never and 7=always)/  4-84 | Bond 22.4 (5.1) Goal 21.2 (6.4)  Task 22.4 (6.1) Total 65.9 (12.7) | Psychotherapy; outpatients with a cockroach phobia and fear of flying or adjustment disorders; treatment with virtual reality and augmented reality | P 75 | 75 % | 34.4 (10.5) |
| Smits (2015) | WAV-12-S-P  Contract, 8  Contact, 4 | Belgium/  Flemish | Cross | Struct V  Int C  Constr V | 5-point scale (1=never and 5=always)/  1-5 | Contract 3.6 (0.8)  Contact 3.9 (0.7) | Psychotherapy; out- and inpatients with different diagnoses; treated with CBT, systematic therapy. and psychodynamic psychotherapy | P 557  T 94 | 60 %  75 % | 38.0 (12.1)  42.2 (12.0) |
| Toste (2015) | CWAI-P  Bond, 4  Goal, 4  Task, 4 | USA/  English | Cross | Struct V  Constr V | 5-point scale (1=never and 5=always)/  1-5 | Bond 4.4 (0.8) Goal 3.9 (0.8) Task 4.2 (0.7) Total 4.1 (0.6) | Education; third-grade students and their teachers from seven schools; teaching | P 430 | 52% | --(--) |
|  | CWAI-T  Bond, 4  Goal, 4  Task, 4 |  |  |  |  | Bond 4.5 (0.6) Goal 4.2 (0.7) Task 4.2 (0.8) Total 4.3 (0.7) |  | T 33 | 94% | --(--) |
| Andrade-González (2016) | WAI-S-P  Bond, 4  Goal, 4  Task, 4 | Spain/ Spanish | Long | Int C  Constr V | 7-point scale (1=never and 7=always)/  1-7 | Bond 6.0 (1.1) Goal 6.1 (1.0)  Task 5.8 (1.1) Total 6.0 (1.0) | Psychology; outpatients with depression; most commonly used approach was integrative | P 36† | 83 % | 42.4 (10.6) |
|  | WAI-S-T  Bond, 4  Goal, 4  Task, 4 |  |  |  |  | Bond 5.8 (0.8) Goal 5.4 (1.0) Task 5.2 (0.9) Total 5.5 (0.9) |  | T 21† | 48 % | 35.0 (10.0) |
| Figueiredo (2016) | WAI-CA-P  Bond, 12  Goal, 12  Task, 12 | Portugal/  Portuguese | Adapt /  Cross | Cont V  Int C  Constr V | 5-point scale (1=never and 5=always)/ 36-180 | Bond 54.0 (5.4) Goal 50.4 (6.4) Task 53.0 (6.2) Total 157.0 (16.2) | Psychology; outpatients, children and adolescents with depression, anxiety or other problems, -- | Pilot Study  P 10 | -- | -- (--) |
|  |  |  |  |  |  |  |  | Clinical study  P 109 | 38 % | 11.3 (2.5) |
| Hukkelberg (2016) | WAI-S-P  Bond, 4  Goal, 4  Task, 4 | Norway/ Norwegian | Long | Struct V | 7-point scale (1=never and 7=always)/  1-7 | -- (--) | Psychology; parents receiving parent management training; the Oregon model (PMTO) | P 259 | -- | -- (--) |
| Hsu (2016) | WAI-S-P  Total, 9 | Hong Kong/ Chinese,  Taiwanese | Adapt /  Cross | Struct V  Int C  Reliab | 7-point scale (1=never and 7=always)/  1-7 | Total 5.1 (0.9) | Psychology; students; following counseling sessions as partial fulfillment of their degree | P 146 | 80 % | 19.2 (1.4) |
| Mallinckrodt (2016) | BAI-P  Bond, 8  Go/Ta, 8 | USA/ English | Cross | Struct V  Int C  Constr V | 5-point scale (1=never and 5=always)/  1-5 | Bonds 3.8 (0.9)  Go/Ta 3.9 (0.8)  Total 3.9 (0.8) | Psychotherapy; out- and inpatients (multiple data sets), community resident; university counseling centers with different problems and treated with different type of therapies | P732†  &  P1054 | 70 % | -- (--) |
| Araujo (2017) | WAI-S-P  Bond, 4  Goal, 4  Task, 4 | Brazil/  Brazilian-Portuguese | Transl/  Pilot/  Long | Cont V  Int C  Reliab  Constr V | 5-point scale (1=never and 5=always)/  4-60 | Total 55.0 (9.0) | Physiotherapy; patients with low back pain; treated by physiotherapists | Pilot Study  P 30 | -- | -- (--) |
|  | WAI-S-T  Bond, 4  Goal, 4  Task, 4 |  |  |  | 7-point scale (1=never and 7=always)/  4-84 | Total 67.5 (8.3) |  | Clinical study  P 100  T 18 | 54 %  78 % | 47.8 (15.4)  25.6 (4.3) |
| Hukkelberg (2017) | WAI-S-P  Bond, 4  Goal, 4  Task, 4 | Norway/ Norwegian | Cross | Struct V  Int C | 7-point scale (1=never and 7=always)/  1-7 | -- (--) | Psychology; parents receiving parent management training; the Oregon model (PMTO) | P 259 | -- | -- (--) |
| Hsu (2017) | WAI-S-T  Bond, 4  Goal/Task, 6 | Honk Kong/ Chinese,  Taiwanese | Long | Struct V  Int C | 7-point scale (1=never and 7=always)/  4-70 | Bond 14.3 (2.6) Goal/Task 21.5 (4.1)  Total 44.7 (7.3) | Psychology; students following counseling sessions as a partial fulfillment of their degree; counseling session given by master students | P 139  T 39 | 79 %  67 % | 19.0 (1.2)  25.8 (4.9) |
| Killian (2017) | WAI-S-P  Bond, 4  Goal, 4  Task, 4 | USA/  English | Cross | Struct V  Int C  Constr V | 7-point scale (1=never and 7=always)/  4-84 | Bond 20.7 (6.9) Goal 21.3 (6.4) Task 19.3 (6.6) Total 61.7 (18.3) | Child protection services; families involved in services; treatment was given by social workers | P 131 | 87 % | 36.0 (10.0) |
|  | WAI-S-T  Bond, 4  Goal, 4  Task, 4 |  |  |  |  | Bond 19.6 (4.8) Goal 19.8 (4.5) Task 18.9 (5.5) Total 58.1 (13.7) |  | T 54 | 81 % | 36.4 (8.5) |
|  | WAI-S-O  Bond, 4  Goal, 4  Task, 4 |  |  |  |  | Bond 19.5 (5.4) Goal 19.9 (5.5) Task 18.5 (5.7) Total 57.8 (15.7) |  | O -- | -- | -- (--) |
| Bat (2018) | AT-WAI-P  Task, 6  Experience, 4  Acceptance, 4 | Israel/  English | Adapt/  Pilot/  Cross | Cont V  Struct V  Intl C  Constr V | 7-point scale (1=never and 7=always)/  1-7 | -- (--) | Art therapy; students following art therapy master program; art therapy | Pilot study  P 40 | -- | -- (--) |
|  |  |  |  |  |  |  |  | P 104 | 92% | 29.2 (5.2) |
| Chen (2018) | WAI-SR-P  Bond, 4  Goal, 4  Task, 4 | China/  Chinese | Transl/  Pilot/  Cross | Struct V  Intl C  Constr V | 5-point scale (1=never and 5=always)/  1-5 | -- (--) | Psychotherapy; inpatients with schizophrenia; treatment in a hospital | Pilot studyP 30 | -- | -- (--) |
|  |  |  |  |  |  |  |  | P 124 | 56 % | 50.8 (14.5) |
| Gülüm (2018) | WAI-S-P  Bond, 4  Goal, 4  Task, 4 | Turkey/ Turkish | Cross | Struct V  Intl C | 7-point scale (1=never and 7=always)/  1-7 | Bond 5.8 (1.0) Goal 6.2 (0.8) Task 5.6 (1.0) Total 5.9 (0.8) | Psychotherapy; patients treated with CBT, schema therapy, or psychodynamic psychotherapy | P 83 | 78 % | 24.2 (6.1) |
|  | WAI-S-T  Bond, 4  Goal, 4  Task, 4 |  |  |  |  | Bond 5.4 (0.8) Goal 5.5 (1.0) Task 4.9 (1.1) Total 5.3 (0.9) |  | T 58 | 88 % | 25.5 (1.4) |
| Karel (2018) | WAV-12-P  Bond, 4  Goal, 4  Task, 4 | Nether-lands/ Dutch | Adapt/  Cross | Cont V  Struct V  Intl C | 5-point scale (1=never and 5=always)/  1-5 | -- (--) | Physiotherapy; patients with shoulder pain in physiotherapy clinics; treated by physiotherapists | P 389 | 57 % | 50.0 (13.0) |
| Paap (2018) | WAI-SR-P-ReD  Bond, 4  Goal, 4  Task, 4 | Nether-lands/  Dutch | Adapt/  Pilot/  Cross | Cont V  Struct V  Intl C  Constr V | 5-point scale (1=never and 5=always)/  1-5 | Bond 4.3 (0.9) Goal 4.4 (0.8) Task 4.0 (0.7) Total 4.2 (0.7) | Rehabilitation; patients with different problems; were treated in an academic rehabilitation center and by different type of rehabilitation professionals | Pilot study  P 25 | -- | -- (--) |
|  |  |  |  |  |  |  |  | P 138 | 54 % | 48 Mdn (32;61 IQR) |
| Santirso (2018) | WAI-S-O  Bond, 4  Goal, 3  Task, 3 | Spain/ Spanish | Cross | Struct V  Intl C  Reliab  ConstrV | 7-point scale (1=never and 7=always)/  1-7 | Bond 4.2 (0.2) Goal 4.2 (0.2) Task 4.2 (0.2) Total 4.2 (0.2) | Psychotherapy; male intimate partner violence offenders; treated with a community-based Batterers’ intervention program | P 140  O 4 | 0 %  -- | 40.3 (11.7)  -- (--) |
| Sturgiss (2018) | WAI-P-GP  Total, 12 | Australia/  English | Adapt/  Cross | Cont V  Struct V  Intl C  Constr V | 5-point scale (1=never and 5=always)/  1-5 | Total 4.3 (0.6) | General practice; patients with different problems; treated in primary care by general practitioners | P 142  T 16 | 62.7 %  50% | --/--  -- (--) |
| Takasaki (2018) | WAI-S-P  Bond, 4  Goal, 4  Task, 4 | Japan/ Japanese | Long | Struct V  Intl C  Reliab | 7-point scale (1=never and 7=always)/  1-7 | -- (--) | Physiotherapy; outpatients with musculoskeletal disorders; undertaking physiotherapy | P 118 | 65 % | 54.5 (16.4) |
| Penedo (2019) | WAI-I-P  Bond, 4  Go/Ta, 8 | Switzerland/  German | Cross | Struct V  Intl C  Constr V | 5-point scale (1=never and 5=always)/  1-5 | Bond 3.6 (1.2) Go/Ta 3.2 (0.9)  Total 3.3 (0.9) | Psychology; patients with mild to moderate depression; followed the online intervention Deprexis | P 223 | 70 % | 44.5 (10.7) |
| Paap (2019) | WAI-SR-P-ReD  Bond, 4  Goal, 4  Task, 4 | Nether-lands/  Dutch | Cross | Constr V | Visual Analog Scale (0= Sometimes and 100= Always)/0-1200 | Bond 340 Mdn (303; 378 IQR)  Goal 364 Mdn (327; 388 IQR)  Task 337 Mdn (307; 376 IQR)  Total 1048 Mdn (931; 1137 IQR) | Rehabilitation; patient with different problems; were treated in an academic rehabilitation center and by different types of rehabilitation professionals | P 152 | 70 % | 51.5 (16.3) |
| Petek (2019) | WAI-SR-P  Bond, 4  Goal, 4  Task, 4 | Slovenia/  Slovene | Transl/  Pilot | Content V | 5-point scale (1=never and 5=always)/  1-5 | -- (--) | Family medicine; practicing and academic family doctors; were invited to participate in the Delphi method | -- | -- | -- (--) |
|  | WAI-SR-T  Bond, 4  Goal, 3  Task, 3 |  |  |  |  |  |  |  |  |  |
| Warlick (2019) | WAIT-3-P  Bond, 1  Goal, 1  Task, 1 | USA/  English | Adapt/  Cross | Struct V  Intl C  Reliab  Constr V | 5-point scale (1=never and 5=always)/  1-5 | Sample 1  Total 3.2 (1.1) | Tobacco counseling; clients with smoking addiction; followed treatment from Amazon’s Mechanical Turk web-based service (MTurk) | P 119 | 37% | 31.4 (--) |
|  | WAIT-12-P  Bond, 4  Goal, 4  Task, 4 |  |  |  |  | Sample 2  Total 3.4 (0.9) | Tobacco counseling; clients with smoking addiction; followed treatment from Amazon’s Mechanical Turk web-based service (MTurk) | P 107 | 35% | P 33.9 (--) |
| Hatcher (2020) | WAI-S-T-IRT  Bond, 4  Goal, 4  Task, 4 | USA/  English | Cross | Struct V  Mea Inv | 7-point scale (1=never and 7=always)/  1-7 | Sample 1  -- (--) | Psychology; patients from 42 college counseling services participating in a psychotherapy research study from 1997-1998 by the National Research Consortium Centers in Higher education,-- | P 688  T 273 | 68%  -- | P 23.3 (--)  --(--) |
|  |  |  |  |  |  | Sample 2  -- (--) | Psychology; patients from alcohol treatment programs from 5 different outpatient sites of Project MATCH in 1998; treated with CBT, TSF, or motivational enhancement therapy | P 610  T 80 | 28%  -- | 38.6 (--)  --(--) |
|  |  |  |  |  |  | Sample 3  -- (--) | Psychology; outpatients with different conditions; treated with psychodynamic, CBT or eclectic/other given by therapists from American Psychological Association Divisions | P251  T 251 | 72%  50% | 40.8 (--)  --(--) |
|  |  |  |  |  |  | Sample 4  -- (--) | Psychology; patients from university outpatient clinical psychology training clinics; psychodynamic treatment | P 231  T 63 | 66%  -- | 28.2 (--)  --(--) |
| Herrero (2020) | WAI-SR-TECH  Bond, 4  Goal, 4  Task, 4 | Multi-center  8 different countries | Cross | Adapt  Struct V  Constr V | 7-point scale (1=never and 7=always)/  1-7 | Total 4.8 (0.9) | Psychology; patients diagnosed with depression included in the clinical trial; treated with blended CBT | P 193 | 64% | 40.4 (12.8) |
| Hunik (2020) | WAI-P-GP  Total, 12 | Australia/English | Cross | Struct V  Intl C  Constr V | 5-point scale (1=never and 5=always)/  1-5 | Bond 4.2 (0.7) Goal 4.3 (0.7) Task 4.4 (0.7)  Total 4.3 (0.7) | General practice; patients with different problems; treated in primary care by general practitioners in 12 general practices | P 146 | 62% | 45 Mdn |
| Miloff (2020) | VTAS-P  Goal/Task 12  Bond 5 | Sweden/  Swedish | Cross | Adapt  Struct V  Intl C  Constr V | 5-point scale (0=Do not agree at all 4=Agree Completely)/  0-4 | Sample 1  Total 44.0 (12.2) | Psychology; patients with spider phobia; gamified virtual reality exposure therapy or virtual therapist support | P 50 | 84% | 34.1 (10.9) |
|  |  |  |  |  |  | Sample 2  Total 45.0 (13.4) | Psychology; patients with spider phobia; gamified virtual reality exposure therapy | P 25 | -- | --(--) |
| Milot-Lapointe (2020) | WAI-S-P  Bond, 4  Goal, 4  Task, 4 | Canada/  French | Long | Struct V  Intl C  Mea Inv | 7-point scale (1=never and 7=always)/  1-7 | Bond 6.0 (0.8) Goal 6.0 (0.8) Task 6.1 (0.8)  Total 6.1 (0.7) | Career counseling; undergraduate or graduate students or participants from 11 organizations; goal-oriented counseling | P 283 | 73% | 28.2 (10.5) |
| Knowles (2020) | CWAI-P  Bond, 4  Goal, 4  Task, 4 | USA/  English | Cross | Struct V  Intl C | 5-point scale (1=never and 5=always)/  1-5 | Bond 4.3 (0.8) Go/Ta 3.9 (0.7)  Total 4.1 (0.8) | Education; students from elementary schools with emotional or behavioral problems without a significant intellectual disability; special education services | P 182 | 24% | --(--) |
|  | CWAI-T  Bond, 4  Goal, 4  Task, 4 |  |  |  |  | Bond 4.1 (0.6) Go/Ta 3.5 (0.6)  Total 3.8 (0.6) |  | T 72 | -- | --(--) |
| Cirasola (2021) | WAI-S-P  Bond, 4  Goal, 4  Task, 4 | UK/  English | Long | Struct V  Mea Inv | 7-point scale (1=never and 7=always)/  1-7 | Total 4.7 (1.8) | Youth psychotherapy; adolescents with depression; treated with CBT, or short-term psychoanalytic psychotherapy, or brief psychosocial interventions | P 338 | 73% | 15.6 (1.4) |
|  | WAI-S-T  Bond, 4  Goal, 4  Task, 4 |  |  |  |  | Total 4.8 (1.3) |  | P 159  T 72 | 69%  -- | 15.6 (1.5)  --(--) |
| Prusińki (2021) | WAI-P  Bond, 12  Goal, 12  Task, 12 | Poland/ Polish | Cross | Adapt  Struct V  Intl C | 7-point scale (1=never and 7=always)/  1-7 | --(--) | Psychotherapy; patients undergoing psychoanalytic, CBT, Ericksonian-, systemic-, and humanistic treatment | P 262 | 50% | 35.2 (11.9) |
|  | WAI-T  Bond, 12  Goal, 12  Task, 12 |  |  |  |  | --(--) |  | T 166 | -- | 42.9 (9.0) |

Legends: Adapt, Adaptation study. AT-WAI, Art Therapy- Working Alliance Inventory. BAI, Brief Alliance Inventory. CBT, Cognitive-Behavioral Therapy. Cont V, Content validity. Constr V, Construct validity. Cross, cross-sectional study. CWAI, Classroom Working Alliance Inventory. Develop, Development study. Go/Ta, Goal and task domain combined. IAT, Inventario de Alianza de Trabajo. IQR, interquartile range. Int C, Internal Consistency. Long, Longitudinal study. Mea Inv, Measurement invariance. Mdn, median. n, number. O, Observer form or observer. P, Patient form or patient (Patients included students, sex offenders, parents, rehabilitation patients, psychiatry patients, parents, families and so forth, anyone who was the client receiving treatment). Par, Parent or guardian. Pilot, Pilot study. Reliab, reliability. SAI, Session Alliance Inventory. SD, standard deviation. Struct V, Structural validity. T, Therapist form or therapists. TAC, Therapeutic Alliance with Clinician. TSF, Twelve-step facilitation. Transl, Translation study. VTAS, Virtual Therapist Alliance Scale. WAI-I, Working Alliance Inventory Internet interventions. WAI-IRT, Working Alliance Inventory Item Response Theory. WAICC, Working Alliance Inventory for Chronic Care (20 versions). WAI-CA, Working Alliance Inventory for Children and Adolescents. WAI, Working Alliance Inventory. WAI-GP, Working Alliance Inventory General Practice. WAI-ReD, Working Alliance Inventory Rehabilitation Dutch Version. WAI-S, Working Alliance Inventory Short Form. WAI-SR, Working Alliance Inventory Short Form Revised. WAI-SR-TECH, Working Alliance Inventory Short Form Revised for online Interventions. WAI-VAR, Working Alliance Inventory applied to virtual and augmented reality. WAIT, Working Alliance Inventory for Tobacco. WAV-12, Werk Alliantie Vragenlijst. WATOCI, Working Alliance Theory of Change Inventory. WAIT, Working Alliance Inventory for Tobacco. -- no information reported in article. *, rounded to 1 decimal. **, based on the original 7-point scale. # for longitudinal studies first reported mean scores. § Child or Adolescent-Medical Doctor versions, other version not noted for improving readability †, overlap (or partially) of participations also included in other studies.

**Table S2.** Content validity of the Working Alliance Inventory and adapted versions.

| **Author ( publication year)** | **WAI-Version** | **Country/ Language** | **Adaption based on version of** | **Asking patients about** | | | **Asking professional about** | | **Methodo-logical quality** | **Overall quality of the develop-ment/ content study #** |
| --- | --- | --- | --- | --- | --- | --- | --- | --- | --- | --- |
|  |  |  |  | **Relevance** | **Compre-hensiveness** | **Compre-hensibility** | **Relevance** | **Compre-hensiveness** |  |  |
| Horvath (1989) | WAI-P | Canada/English | Original Version | N | N | N | N | N | Inadequate | Insufficient |
| Horvath (1989) | WAI-T | Canada/English | Original Version | N | N | N | N | N | Inadequate | Insufficient |
| Tichenor (1989) | WAI-O | USA/English | Horvath (1989) | N | N | N | N | N | Inadequate | Insufficient |
| Ely (2005) | WAICC* | USA/ English | Horvath (1989) | D | N | D | D | D | Doubtful | Insufficient |
| Guédeney (2005) | WAI-P | France/French | Horvath (1989) | N | N | I | I | N | Inadequate | Insufficient |
| Guédeney (2005) | WAI-T | France/French | Horvath (1989) | N | N | I | I | N | Inadequate | Insufficient |
| Bedregal (2006) | TAC | USA/ Spanish | Neale (1995) | N | N | N | N | N | Inadequate | Insufficient |
| Corbiѐre (2006) | WAI-S-P | Canada/ French | Tracey (1989) | N | N | N | D | D | Doubtful | Insufficient |
| Corbiѐre (2006) | WAI-S-T | Canada/ French | Tracey (1989) | N | N | N | D | D | Inadequate | Insufficient |
| Soygüt (2008) | WAI-P | Turkey/Turkish | Horvath (1989) | N | N | D | N | D | Inadequate | Insufficient |
| Soygüt (2008) | WAI-T | Turkey/Turkish | Horvath (1989) | N | N | D | N | D | Inadequate | Insufficient |
| Wilmers (2008) | WAI-SR-P | Germany/German | Hatcher (2006) | N | N | N | N | N | Inadequate | Insufficient |
| Soygüt (2009) | WAI-O | Turkey/Turkish | Horvath (1989) | N | N | N | N | D | Inadequate | Insufficient |
| Perdrix (2010) | WAI-SR-P | Switzerland/French | Hatcher (2006) | N | N | N | N | D | Inadequate | Insufficient |
| Vöhringer (2013) | WAI-O | Chile/ Spanish | Horvath (1989) | N | N | N | N | N | Inadequate | Insufficient |
| Andrade-González (2015) | WAI-P | Spain/Spanish | Horvath (1989) | N | N | N | I | A | Inadequate | Insufficient |
| Andrade-González (2015) | WAI-T | Spain/Spanish | Horvath (1989) | N | N | N | I | A | Inadequate | Insufficient |
| Lamers (2015) | WAV-12R | Netherlands/  Dutch | Tracey (1989)/ Vertommen (1990) | N | N | D | N | D | Inadequate | Insufficient |
| Miragall (2015) | WAI-VAR | Spain/Spanish | Tracey (1989) /Corballa (2011) | N | N | N | N | N | Inadequate | Insufficient |
| Figueiredo (2016) | WAI-CA | Portugal/  Portuguese | Machado (1999) | N | N | D | N | N | Inadequate | Insufficient |
| Araujo (2017) | WAI-S-P | Brazil/Brazilian-Portuguese | Tracey (1989) | N | N | I | N | N | Inadequate | Insufficient |
| Araujo (2017) | WAI-S-T | Brazil/Brazilian-Portuguese | Tracey (1989) | N | N | I | N | N | Inadequate | Insufficient |
| Bat (2018) | AT-WAI | Israel/English | Horvath (1989) | N | N | N | D | N | Inadequate | Insufficient |
| Chen (2018) | WAI-SR-P | China/Chinese | Hatcher (2006) | N | N | D | N | N | Inadequate | Insufficient |
| Karel (2018) | WAV-12 | Netherlands/  Dutch | Tracey (1989)/ Vertommen (1990) | N | N | D | N | D | Inadequate | Insufficient |
| Paap (2018) | WAI-P-ReD | Netherlands/ Dutch | Hatcher (2006)/ Vertommen (1990) | N | N | D | D | D | Doubtful | Insufficient |
| Sturgiss (2018) | WAI-SR-P | Austria/English | Hatcher (2006) | D | D | D | D | D | Doubtful | Doubtful |
| Petek (2019) | WAI-SR-P | Slovenia/Slovene | Hatcher (2006) | N | N | N | D | D | Doubtful | Insufficient |
| Petek (2019) | WAI-SR-T | Slovenia/Slovene | Hatcher (2006) | N | N | N | D | D | Doubtful | Insufficient |
| Warlick (2019) | WAIT-12-P | USA/ English | Hatcher (2006) | N | N | N | N | N | Inadequate | Insufficient |
| Herrero (2020) | WAI-P-SR-TECH | Multicenter study 8 different countries | Hatcher (2006) | N | N | N | N | N | Inadequate | Insufficient |
| Miloff (2020) | VTAS-P | Sweden/Swedish | Horvath (1989)/ Hatcher (2006) | N | N | N | N | N | Inadequate | Insufficient |
| Prusińki (2021) | WAI-P | Poland/Polish | Horvath (1989) | N | N | N | N | N | Inadequate | Insufficient |
| Prusińki (2021) | WAI-T | Poland/Polish | Horvath (1989) | N | N | N | N | N | Inadequate | Insufficient |

Legends: AT-WAI, Art Therapy- Working Alliance Inventory. O, observer form. P, patients form. T, Therapist form. TAC, Therapeutic Alliance with Clinician. VTAS, Virtual Therapist Alliance Scale. WAICC, Working Alliance Inventory for Chronic Care. WAI-CA, Working Alliance Inventory for Children and Adolescents. WAI, Working Alliance Inventory. WAI-I, Working Alliance Inventory Internet interventions. WAI-S, Working Alliance Inventory Short form. WAI-SR, Working Alliance Inventory Short Form Revised. WAI-SR-TECH, Working Alliance Inventory Short Form Revised for online Interventions. WAI-ReD, Working Alliance Inventory Rehabilitation Dutch Version. WAI-VAR, Working Alliance Inventory applied to virtual and augmented reality. WAIT, Working Alliance Inventory for Tobacco. WAV-12, Werk alliantie vragenlijst 12. WAV-12R, Werk alliantie vragenlijst 12 revised. Note: *WAICC included all 16 version. V=very good; A= adequate; D=doubtful; I=inadequate; N= not conducted. #, An overall sufficient , insufficient, or inconsistent rating was determined for relevance, comprehensiveness, and comprehensibility of the WAI-versions by jointly assessing all results and reviewers’ rating on the same version. For more details regarding rating of methodological quality see Table 1.**Table S3.** Structural validity of the Working Alliance Inventory and adapted versions.

| **Author (Publication year)** | ***n* for analysis** | **Method** | **Results** | **Expl. variance** | **Methodo-logical quality** | **Rating#** |
| --- | --- | --- | --- | --- | --- | --- |
| Tracey (1989) | 84 | Confirm | Three models were compared (one-factor, three- factor and hierarchical Bi-level model). Bi-level model fitted the data best; GFI=0.88; TLI=0.91. | N/A | Inadequate | - |
| Hatcher (1995) | 144 | Confirm | Three models were compared one, two, and three-factor model) in a nested design. Three-factor model fitted the data best; GFI= 0.98; CFI= 1.0; RMSR = 0.02. | 67% | Inadequate | + |
| Hatcher (1996) | 231 | Explr | Two factors were extracted (goal and task combined) using principal component analysis with varimax rotation; > 1 eigenvalue (15.0 and 2.5). | 49% | Adequate | - |
| Hatcher (1999) | 251 | Explr | Four factors were extracted (shared goals, bond, goal and task disagreement, and therapist confidence in treatment) using principal component analysis with varimax rotation | 48% | Doubtful | - |
| Andrusyna (2001) | 95 | Explr | Two factors were extracted (goal and task combined) using principal component analysis with varimax rotation; > 1 eigenvalues (7.0 and 1.8). | 73% | Adequate | - |
| Santibánez (2003) | 72 | Explr | IAT-S-P  One factor was extracted using principal components analyses; > 1 eigenvalues (2.5). | 83% | Inadequate | - |
| Santibánez (2003) | 72 | Explr | IAT-S-T  One factor was extracted principal components analyses; > 1 eigenvalues (2.6). | 86% | Inadequate | - |
| Corbella (2004) | 102 | Explr | Three factors were extracted using principal component analysis with varimax rotation; > 1 eigenvalues (5.7, 2.8 and 2.6). Factors did not discriminate between items belonging to different domains. | 65% | Adequate | - |
| Guédeney (2005) | 130 | Explr | Two factors were extracted using principal component analysis with varimax rotation. Factor one reflected the bond and factor two reflected negative emotions roused by the idea of assistance. | 45% | Inadequate | - |
| Corbiѐre (2006) | 150 | Confirm | WAI-S-P  Three models were compared (one, two, and three- factor model) No factor model fitted the acceptance criteria. With adjustments, three-factor model fitted the data best; CFI=0.94; RSMEA=0.09. | N/A | Very good | - |
| Corbiѐre (2006) | 150 | Confirm | WAI-S -T  Three models were compared (one, two, and three- factor model) No factor model fitted the acceptance criteria. With adjustments, three-factor model fitted the data best; CFI=0.95; RSMEA=0.08. | N/A | Very good | - |
| Bedregal (2006) | 103 | Explr | One factor was extracted using principal component analysis; > 1 eigenvalue. | 78% | Doubtful | - |
| Hatcher (2006) | 235 | Confirm | Evaluated factor structure WAI and WAI-S in two samples. The hypothesized structures were not confirmed. An alternative 12-item WAI (WAI-SR) was developed in sample 1 and cross validated in sample 2, which confirmed the three- factor structure; TLI=0.94; CFI=0.95; RSMEA=0.08. | N/A | Adequate | - |
| Wilmers (2008) | 243 | Confirm | Three factors model was tested in inpatients. The tested model fitted the data; CFI=0.95; RSMEA=0.09; SRMR=0.05. | N/A | Very good | - |
|  | 88 | Confirm | Three factors model was tested in outpatients. The tested model fitted the data; CFI=0.95; RSMEA=0.08; SRMR=0.06. | N/A | Very good | - |
| Soygüt (2008) | 63 | Explr | Three factors were extracted using principal component analysis with varimax rotation; task, goal and bond. | 46% | Inadequate | - |
| Soygüt (2009) | 10 | Explr | Two factors were extracted (goal and task combined) using principal component analysis with varimax rotation; > 1 eigenvalues. (5.9 and 4.8). | 89% | Inadequate | - |
| Stinckes (2009) | 256 | Confirm | Three models were compared (one, two, and three- factor model). Two- factor and three - factor model both had an acceptable fit. GFI=0.90; RSMEA=0.09. | N/A | Very good | - |
| Munder (2009) | 88 | Confirm | Three models were compared (one, two, and three- factor model) in outpatients. The three- factor model fitted the data, AIC values were lower, indicating a better fit; CFI=0.95; TLI=0.93; RSMEA=0.08. | N/A | Very good | - |
|  | 234 | Confirm | Three models were compared (one, two, and three- factor model) in inpatients. The three-factor model fitted the data, AIC values were lower, indicating a better fit; CFI=0.95; TLI=0.93; RSMEA=0.09. | N/A | Very good | - |
| Perdrix (2010) | 188 | Explr | WAI  Four factors were extracted using principal component analysis with oblique rotation; > 1 eigenvalues. (8.3, 2.6, 2.2 and 1.8) Factors did not discriminate between items belonging to different domains. | 41% | Adequate | - |
|  | 283 | Explr | WAI-SR  Three factors were extracted (task, goal and bond) using principal component analysis with oblique rotation; > 1 eigenvalues. (4.5, 1.4 and 1.3). | 60% | Adequate | - |
|  | 188 | Confirm | WAI  Two models were compared (three- factor & hierarchical Bi-level model). No model fitted the acceptance criteria. With adjustments, three- factor model fitted the data best; TLI= 0.84; CFI=0.85; RMSEA=0.05. | N/A | Adequate | - |
|  | 283 | Confirm | WAI  Two models were compared (three-factor & hierarchical Bi-level model) for the total sample and for both career and personal sample. No model fitted acceptance criteria. With adjustments both models similar fitted the data; TLI= 0.94; CFI=0.96; RMSEA=0.06. | N/A | Adequate | - |
| Tatman (2010) | 182 | Confirm | Three models were compared (1, 2 and 3 factor model). The 1 factor model fitted the data best; TLI= 0.93; CFI=0.95; RMSEA=0.11. | N/A | Very good | - |
| Corbella (2011) | 229 | Explr | Two factors were extracted using principal component analysis with varimax rotation; > 1 eigenvalues. (7.0 and 1.1) Factors did not discriminate between items belonging to different domains. | 61% | Adequate | - |
| Ross  (2011) | 49 | Confirm | WAI-S-P  Three models were compared (one, two, and three- factor model). The two-factor model (combined goal/task) fitted the data best; GFI= 0.62; CFI=0.80; RMSEA=0.24. Removing Item 4 improves model fit across all perspective. | N/A | Inadequate | - |
|  | 68 | Confirm | WAI-S-T  Three models were compared (one, two, and three- factor model). Two-factor model (combined goal/task) fitted the data best; GFI= 0.68; CFI=0.80; RMSEA=0.17. Removing Item 4 improves model fit across all perspective. | N/A | Inadequate | - |
|  | 68 | Confirm | WAI-S-O  Three models were compared (one, two, and three- factor model). The two- factor model (combined goal/task) fitted the data best; GFI= 0.72; CFI=0.91; RMSEA=0.15. Removing Item 4 improves model fit across all perspective. | N/A | Inadequate | - |
| Hall (2012) | 206 | Rasch | Five items failed to fit the model, these 5 items were removed. Four pairs of items overlapped along the hierarchy; two redundant items were removed. Using principal component analysis of the nine remaining items confirmed no observed sub-dimensions present in the 9-item WATOCI. > 1 eigenvalues (1.7). | 64% | Very good | ? |
| Falkenström (2015)a | Sample 1 235 | Confirm | Three models were compared. The three- factor model fitted the data best. BIC 7795.4 one factor. BIC 7634.1 two factors. BIC 7630.6 three factors. Also AIC was lowest in the three-factor model. | N/A | Doubtful | ? |
|  | Sample 2  634 | Confirm | Three models were compared. The three- factor model fitted the data best. BIC 18123.6 one factor. BIC 17398.4 two factors. BIC 17347.0 three factors. Also AIC was lowest in the three- factor model. | N/A | Doubtful | ? |
|  | Sample 3  234 | Confirm | Three models were compared. The three- factor model fitted the data best. BIC 7422.6 one factor. BIC 7204.0 two factors. BIC 7174.9 three factors. Also AIC was lowest in the three-factor model. | N/A | Doubtful | ? |
| Falkenström (2015)b | Sample 1 1061 | Confirm | Four models were compared. Only the bifactor model with correlation between group factors showed adequate model fit. | N/A | Doubtful | ? |
|  | Sample 1 235 | Confirm | Four models were compared. The bifactor model with orthogonal group factors showed a model fit that was almost as good as the bifactor model with correlated group factors. | N/A | Doubtful | ? |
|  | Sample 3 234 | Confirm | Four models were compared. The bifactor model with orthogonal group factors showed a model fit that was almost as good as the bifactor model with correlated group factors. | N/A | Doubtful | ? |
| Lamers (2015) | 80 | Confirm | WAV-12R Team version  Three models were compared (one, two, and three-factor model). The three- factor model fitted the data best; CFI=0.96; RMSEA=0.09; SRMR= 0.06. | N/A | Adequate | - |
|  | 73 | Confirm | WAV-12R Caregiver version  Three models were compared (one, two, and three-factor model). The two- factor model fitted the data best. After adjustments the three-factor model had a better fit; CFI=0.96; RMSEA=0.08; SRMR= 0.06. | N/A | Adequate | - |
| Mirigall (2015) | 75 | Explr | One factor was extracted using maximum likelihood estimation extraction method; > 1 eigenvalue (6.9). | 54% | Adequate | - |
| Smits (2015) | 557 | Confirm | Four models were compared. The two, three, and bi-level factor models scored similar. The two- factor model was more parsimonious; CFI=0.97; RMSEA=0.10; SRMR= 0.06. | N/A | Very good | - |
| Toste (2015 | 430 | Confirm | CWAI-P  Three models were compared (one, two, and three-factor model). The two-factor model (goal/task combined) fitted the data best CFI=0.98; TLI=0.98; RMSEA=0.02; AIC=14772.8. | N/A | Very good | + |
|  | 430 | Confirm | CWAI-T  Three models were compared (one, two, and three-factor model). The two- factor model (goal/task combined) fitted the data best CFI=0.94; TLI=0.93; RMSEA=0.07; AIC=7057.2. | N/A | Very good | - |
| Hukkelberg (2016) | 206 | Confirm | Three models were compared (one, two, and three-factor model). No model fitted the acceptance criteria. With adjustments oblique three-factor model fitted the data best; CFI=0.98; TLI=0.97; RMSEA=0.09. | N/A | Very good | - |
| Hsu (2016) | 146 | Confirm/ Explr | Three-factor model could not be confirmed. In the Explr analyses two factors were extracted. Factors did not discriminate between items belonging to different domains. | 58% | Doubtful | - |
| Mallinckrodt (2016) | 1786 | Rasch | Two-factor model (bond and task and goal together) fitted the data best and BAI was developed to provide a more stable structure across the domains and items. | N/A | Very good | ? |
| Hukkelberg (2017) | 259 | Confirm | Six models were compared. The Bi-factor exploratory structural equation model fitted the data best; CFI=0.99; TLI=0.99; RMSEA=0.03. | N/A | Very good | + |
| Hsu (2017) | 139 | Explr | One factor was extracted using principal component analysis with varimax rotation. | 61% | Inadequate | - |
| Kilian (2017) | 131 | Confirm | WAI-S Family Reported  Two model were compared (two and three- factor model). The three-factor model fitted the data best; CFI=0.93; TLI=0.90; RMSEA= 0.11. | N/A | Doubtful | - |
|  | 54 | Confirm | WAI-S Social Worker Reported  Two model were compared (two and three-factor model). The three- factor model fitted the data best; CFI=0.97; TLI=0.96; RMSEA= 0.08. | N/A | Doubtful | - |
|  | 131 | Confirm | WAI-S Observer Reported  Two factor models were compared (two-and three-factor model). The three- factor model fitted the data best; CFI=0.97; TLI=0.96; RMSEA= 0.09. | N/A | Doubtful | - |
| Bat (2018) | 104 | Explr | Three factors was extracted using principal component analysis with varimax rotation; > 1.7 eigenvalues. (4.4, 2.3 and 1.7). | 60% | Adequate | - |
| Chen (2018) | 124 | Confirm | Two models were compared. The two-factor (1. bond and 2. task and goal) model higher adjusted goodness of fit index; CFI= 0.94; TFI= 0.92.RMSEA = 0.08. | 64% | Very Good | - |
| Gülüm (2018) | 83 | Confirm | WAI-S-P and WAI-SR-P  Two models were compared. With modification, both models fitted the data in both versions; TLI= 0.95; CFI= 0.97; RMSA= 0.56. | N/A | Inadequate | - |
|  | 83 | Confirm | WAI-S-T and WAI-SR-T  Two models were compared. With modification both models fitted the data in both versions; TLI=0.96; CFI= 0.97; RMSA= 0.60. | N/A | Inadequate | - |
| Karel (2018) | 274 | Rasch | Good discriminative abilities for the lower end of the construct. Due the missing data among the items and observed ceiling effects modification of the WAI was needed. | N/A | Very good | - |
| Paap (2018) | 138 | Confirm | Three models were compared (one, two, and three-factor model). The two-factor model (Bond and task and goal combined) fitted the data best; CFI=0.92; TLI=0.90; RMSEA=0.10. | N/A | Very good | - |
| Santirso (2018) | 140 | Confirm | Five models were compared. The three-factor model (Agreement and Bond) fitted the data best;  BIC -1300.0. | N/A | Doubtful | ? |
| Sturgiss (2018) | 142 | Confirm | Two models (one-factor and three-factor model) were compared. Unable to separate the three domains. One overall factor was identified. | N/A | Doubtful | ? |
| Takasaki (2018) | 118 | Explr | Two factors was extracted using principal component analysis with varimax rotation. | N/A | Doubtful | ? |
| Penedo (2019) | 223 | Confirm | Two models (two- factor and three- factor model) were compared. The two-factor model (goal and task combined) fitted the data best; CFI=0.99; TLI=0.99; RMSEA=0.10; SRMR=0.06. | N/A | Very good | - |
| Warlick (2019) | 107 | Confirm | Two models (one-factor and three-factor model) were compared. Three-factor model fitted the data best; CFI=0.94; TLI=0.93; RMSEA= 0.11; SRMR= 0.04. | N/A | Very good | - |
| Hatcher (2020) | Sample 1  P 686  T 131 | Explr | WAI-S-T-IRT  Development based on WAI-T using multi-level IRT method. Multi-level Bayesian Structural Equation Modeling was used accounting for therapist rated effects. | N/A | Very good | ? |
|  | Sample 2-4 combined  P 1117  T 394 | Confirm | WAI-S-T-IRT  Comparison of WAI-S-T, WAI-SR-T and WAI-S-T-IRT, two-factor model (goal and task combined) was tested. All three measures showed satisfactory fit. The WAI-S-T-IRT fit slightly better. CFI=0.95; RMSEA= 0.72; SRMR= 0.04. | N/A | Very good | - |
| Herrero (2020) | 193 | Explr | WAI-SR-P-TECH  One single factor was extracted using principal component analysis with factorial rotation. | 73% | Adequate | - |
| Hunik (2020) | 142+139 (Sturgiss prev. sample) | Explr | WAI-S-P  One single factor was extracted using principle component analyses. | N/A | Inadequate | - |
| Miloff (2020) | 75 | Explr | VTAS-P  Two factors were extracted (1 task, goal and copresence categories\|2 bond and empathy) using principle component analyses with oblimin rotation. | 53% | Inadequate | - |
| Milot-Lapointe (2020) | 283 | Confirm | WAI-S-P  Eight models were compared (one-factor, two-factor, three-factor, and bilevel hierarchical models). The adjusted three- factor and adjusted Bilevel hierarchical models had the best fit to the data; CFI=0.95; TLI=0.93; RMSEA=0.07; SRMR=0.05 (both models had an identical fit). | N/A | Very good | - |
| Knowles (2020) | P 182 | Confirm | CWAI-P  Evaluation of two-factor model (goal and task combined). CFI=0.97; TLI=0.96; RMSEA=0.07. | N/A | Very good | + |
|  | T 76 | Confirm | CWAI-T  All included students were nested within special educational teachers. Evaluation of two-factor model (goal and task combined); CFI=0.98; TLI=0.97; RMSEA=0.10. | N/A | Very good | - |
| Cirasola (2021) | 338 | Confirm | WAI-S-P  Four models were compared (one-factor, two-factor, three-factor, and bifactor model). The two-factor model (Bond and task and goal combined) fitted the data best; CFI=0.97; TLI=0.95; RMSEA=0.08; SRMR= 0.04; AIC= 8687.9. | N/A | Very good | - |
|  | P 159  T 72 | Confirm | WAI-S-T  Four models were compared (one-factor, two-factor, three-factor, and bifactor model). The two- factor model (Bond and task and goal combined) fitted the data best; CFI=0.92; TLI=0.91; RMSEA=0.11; SRMR= 0.05; AIC= 4327.4. | N/A | Very good | - |
| Prusińki (2021) | 262 | Confirm | WAI-P  three-factor model was tested; ; CFI=0.24; GFI=0.81; RMSEA=0.04. | N/A | Very good | - |
|  | 166 | Confirm | WAI-T  three-factor model was tested; ; CFI=0.27; TLI=0.82; RMSEA=0.04. | N/A | Very good | - |

Legends: AIC, Akiake information criterion. BAI, Brief Alliance Inventory. BIC, Bayesian information criterion. CFI*,* Comparative Fit Index. Confirm, Confirmatory factor analysis. CWAI, Classroom Working Alliance Inventory. Explr, Explorative factor analysis. GFI, Goodness of fit index. IAT, Inventario de Alianza de Trabajo. *n*, number of patients. N/A, Not applicable. O, Observer form. Form. P, Patient form. Rasch, Rasch analysis. RMSR = Root mean square residual. RMSEA*,* root mean square error of approximation. SRMR, standardized root-mean-square residual. RMSR*,* root mean square error of approximation*.* T, Therapists form. VTAS, Virtual Therapist Alliance Scale. WAI, Working Alliance Inventory. WAI-S, Working Alliance Inventory short form. WAI-GP, Working Alliance Inventory General Practice. WAI-SR, Working Alliance Inventory short form revised. WAI-SR-TECH, Working Alliance Inventory Short Form Revised for online Interventions. WAI-IRT, Working Alliance Inventory Item Response Theory. WATOCI, Working Alliance Theory of Change Inventory. WAV-12R, Werk Alliantie Vragenlijst 12 revised. TLI: Tucker-Lewis index. Notes; “+”, sufficient; “?”, Indeterminate; “-“, insufficient. When more types of WAI versions are used in one study the different used versions are reported in the table. #For more details regarding ratings see Table 1.

**Table S4.** Internal consistency (Cronbach’s α) of the Working Alliance Inventory and adapted versions.

| **Author ( publication year)/**  **WAI-Version** | ***n* for analysis** | **Total score (α)** | **Domain scores (α)** | | | | **Methodological quality** | **Rating#** |
| --- | --- | --- | --- | --- | --- | --- | --- | --- |
|  |  |  | **Bond** | **Goal** | **Task** | |  |  |
| Horvath (1989)/WAI-P | 44 | 0.93 | 0.89 | 0.92 | 0.92 | | Doubtful | ? |
| Horvath (1989)/WAI-T | 29 | 0.87 | 0.68 | 0.87 | 0.87 | | Doubtful | - |
| Tichenor (1989)/WAI-P | 8 | 0.96 | -- | -- | -- | | Insufficient | ? |
| Tichenor (1989)/WAI-T | 8 | 0.95 | -- | -- | -- | | Insufficient | ? |
| Tichenor (1989)/WAI-O | 8 | 0.98 | -- | -- | -- | | Insufficient | ? |
| Hatcher (1999)/WAI-T | Sample 1: 251 | -- | 0.75 | 0.82 | | | Doubtful | ? |
|  | Sample 2: 259 | -- | 0.85 | 0.84 | | | Doubtful | ? |
| Cecero (2001)/ WAI-O | 60 | 0.98 | 0.97 | 0.93 | 0.96 | | Very good | ? |
| Cecero (2001)/ WAI-P | 52 | 0.94 | 0.89 | 0.77 | 0.82 | | Very good | ? |
| Cecero (2001)/ WAI-T | 58 | 0.95 | 0.83 | 0.91 | 0.87 | | Very good | ? |
| Busseri (2003)/ WAI-P | 54 | 0.95 | 0.83 | 0.90 | 0.91 | | Very good | ? |
| Busseri (2003)/WAI-T | 54 | 0.94 | 0.71 | 0.92 | 0.87 | | Very good | ? |
| Busseri (2003)/WAI-S-P | 54 | 0.91 | 0.80 | 0.73 | 0.86 | | Very good | ? |
| Busseri (2003)/WAI-P-T | 54 | 0.91 | 0.77 | 0.81 | 0.89 | | Very good | ? |
| Santibánez (2003)/IAT-S-P | 117 | 0.90 | 0.67 | 0.78 | 0.80 | | Very good | - |
| Santibánez (2003)/IAT-S-P | 117 | 0.93 | 0.70 | 0.85 | 0.85 | | Very good | ? |
| Corbella (2004)/WATOCI* | 102 | 0.93 | 0.91 | 0.85 | 0.86 | | Very Good | ? |
| Goldberg (2004)/ WAI-S | 31 | 0.86 | 0.82 | 0.27 | 0.86 | | Doubtful | - |
| Goldberg (2004)/WAI-T | 29 | 0.92 | 0.65 | 0.83 | 0.86 | | Doubtful | - |
| Ely (2005) WAICC scales** | 27 | 0.76-0.88 | -- | -- | -- | | Insufficient | ? |
| Bedregal (2006)/ TAC | 103 | 0.96 | -- | -- | -- | | Very good | ? |
| Corbiѐre (2006)/WAI-S-P | 150 | 0.88 | 0.77 | 0.67 | 0.64 | | Very good | - |
| Corbiѐre (2006)/WAI-S-T | 150 | 0.93 | 0.78 | 0.81 | 0.86 | | Very good | ? |
| Hatcher (2006)/WAI-SR-P | Sample 1: 231 | 0.91 | 0.90 | 0.87 | 0.85 | | Very good | ? |
|  | Sample 2: 235 | 0.92 | 0.85 | 0.85 | 0.87 | | Very good | ? |
| Soygüt (2008)/WAI-P | 63 | 0.90 | 0.78 | 0.74 | 0.81 | | Adequate | ? |
| Soygüt (2008)/WAI-T | 63 | 0.96 | 0.83 | 0.87 | 0.94 | | Adequate | ? |
| Soygüt (2009)/WAI-O | 10 | 0.91 | 0.90 | 0.88 | 0.92 | | Insufficient | ? |
| Stinckens (2009)/WAI-P | 256 | -- | 0.82 | 0.83 | 0.85 | | Doubtful | ? |
| Munder (2009)/WAI-SR-P | Sample 1: 88 | 0.90 | 0.82 | 0.81 | 0.85 | | Very good | ? |
|  | Sample 2: 243 | 0.93 | 0.83 | 0.91 | 0.86 | | Very good | ? |
| Pedrix (2010)/ WAI-SR-P | Sample 1: 188 | 0.76 | 0.52 | 0.58 | 0.71 | | Very good | - |
|  | Sample 2: 95 | 0.92 | 0.80 | 0.88 | 0.86 | | Very good | ? |
| Tatman (2010)/WAI-SR-P | Sample 1: 182 | -- | 0.93 | 0.93 | 0.90 | | Doubtful | ? |
|  | Sample 2: 174 | 0.97 | -- | -- | -- | | Doubtful | ? |
|  | Sample 3: 202 | 0.93 | -- | -- | -- | | Doubtful | ? |
| Corbella (2011)/WAI-S-P | 229 | 0.91 | 0.86 | 0.85 | 0.88 | | Very good | ? |
| Hall (2012) WAIOCI | 206 | 0.82 | -- | -- | -- | | Insufficient | - |
| Vöhringer (2013)/ WAI-O | 59 | 0.97 | 0.93 | 0.95 | 0.94 | | Doubtful | ? |
| Andrade-González (2015)/ WAI-P | 36 | 0.96 | 0.93 | 0.90 | 0.89 | | Doubtful | ? |
| Andrade-González (2015)/ WAI-T | 21 | 0.96 | 0.86 | 0.93 | 0.90 | | Doubtful | ? |
| Falkenström (2015)b/ SAI-P** | 1530 | 0.89-0.94 | 0.85 | 0.90 | | | Very good | ? |
| Lamers (2015)/WAV-12-Team version | 78 | 0.93 | 0.97 | 0.78 | 0.87 | | Adequate | ? |
| Lamers (2015)/WAI-S-P- Parents version | 67 | 0.93 | 0.87 | 0.84 | 0.92 | | Adequate | ? |
| Miragall (2015)/WAI-VAR-P | 75 | 0.91 | 0.86 | 0.92 | 0.70 | | Adequate | ? |
| Smits (2015)/WAV-12-S-P | 557 | -- | 0.81 | 0.90 | | | Doubtful | ? |
| Andrade-González(2016)/WAI-S-P | 36 | 0.93 | 0.86 | 0.80 | 0.84 | | Doubtful | ? |
| Andrade-González(2016)/WAI-S-T | 21 | 0.94 | 0.75 | 0.87 | 0.89 | | Doubtful | ? |
| Figueiredo (2016) WAI-CA | 109 | 0.89 | 0.73 | 0.71 | 0.79 | | Very good | ? |
| Hsu (2016) | 146 | 0.89 | -- | -- | -- | | Very good | ? |
| Mallinckrodt (2016)/BAI | 1786 | -- | 0.93 | 0.89 | | | Doubtful | ? |
| Araujo (2017)/ WAI-S-P | 95 | -- | 0.62 | 0.58 | 0.52 | | Doubtful | - |
| Araujo (2017)/ WAI-S-T | 18 | -- | 0.58 | 0.79 | 0.81 | | Doubtful | - |
| Hukkelberg (2017)/ WAI-S-P | 259 | 0.96† | 0.92† | 0.87† | 0.88† | | Very good | + |
| Hsu (2017)/ WAI-S-T | 139 | 0.91 | 0.77 | 0.88 | | | Very good | ? |
| Kilian (2017)/WAI-S- P | 131 | 0.94 | 0.89 | 0.82 | 0.84 | | Very good | ? |
| Kilian (2017)/WAI-S- T | 131 | 0.95 | 0.85 | 0.79 | 0.95 | | Very good | ? |
| Kilian (2017)/WAI-S- O | 131 | 0.97 | 0.93 | 0.92 | 0.95 | | Very good | ? |
| Bat (2018)/ AT-WAI | 104 | 0.84 | 0.72 | 0.78 | 0.86 | | Very good | ? |
| Chen (2018)/WAI-SR-P | 124 | 0.86 | 0.79 | 0.91 | | | Very good | ? |
| Gülüm (2018)/ WAI-S-P | 83 | 0.86 | 0.67 | 0.65 | 0.71 | | Doubtful | - |
| Gülüm (2018)/ WAI-S-T | 58 | 0.90 | 0.65 | 0.81 | 0.83 | | Doubtful | - |
| Karel (2018)/WAV-12-P | 274 | 0.89 | -- | -- | -- | | Very good | ? |
| Paap (2018)/ WAI-ReD | 138 | 0.93 | 0.80 | 0.86 | 0.85 | | Very good | ? |
| Santirso (2018)/WAI-S-O | 140 | 0.96 | -- | -- | -- | | Very good | ? |
| Sturgiss (2018)/ WAI-SR-P | 142 | 0.95 | -- | -- | -- | | Very good | ? |
| Takasaki (2018)/WAI-S-P | 118 | 0.88 | -- | -- | -- | | Very good | ? |
| Penedo (2019)WAI-I-P | 223 | 0.93 | 0.89 | 0.93 | | | Very good | ? |
| Warlick (2019)/WAIT-12-P | 119 | 0.90** | -- | -- | -- | | Very good | ? |
| Warlick (2019)/WAIT-3-P | 107 | 0.80** | -- | -- | -- | | Very good | ? |
| Hunik (2020)/WAI-S-P-GP | 142 | 0.96 | -- | -- | -- | | Very good | ? |
| Miloff (2020)/VTAS-P | 75 | 0.92 | 0.71 | 0.93 | | | Adequate | ? |
| Milot-Lapointe (2020)/WAI-S-P | 217 | 0.93 | 0.93 | 0.68 | | 0.86 | Very good | - |
| Knowles (2020)/CWAI-P | 182 | -- | 0.82 | 0.79 | | | Doubtful | ? |
| Knowles (2020)/CWAI-T | 76 | -- | 0.81 | 0.89 | | | Doubtful | ? |
| Prusińki (2021)/WAI-P | 262 | 0.98 | 0.93 | 0.93 | | 0.93 | Very good | ? |
| Prusińki (2021)/WAI-T | 166 | 0.97 | 0.94 | 0.92 | | 0.92 | Very good | ? |

Legends: α, Chronbach’s alpha. AT-WAI, Art Therapy Working Alliance Inventory. BAI, Brief Alliance Inventory. CWAI, Classroom Working Alliance Inventory. IAT, Inventario de Alianza de Trabajo. *n*, number of patients. O, observer form. P, patient form. SAI, Session Alliance Inventory. T, therapists form. TAC, Therapeutic Alliance with Clinician. VTAS, Virtual Therapist Alliance Scale. WAI-CA, Working Alliance Inventory for Children and Adolescents. WAICC, Working Alliance Inventory for Chronic Care. WAIOCI, Working Alliance Theory of Change Inventory. WAIT, Working Alliance Inventory Internet interventions. WAI-ReD, Working Alliance Inventory Rehabilitation Dutch Version. WAI, Working Alliance Inventory. WAI-S, Working Alliance Inventory Short form. WAI-SR, Working Alliance Inventory Short Revised Form. Working Alliance Inventory General Practice. WAI-VAR, Working Alliance Inventory applied to virtual and augmented reality. WAIT, Working Alliance Inventory for Tobacco. WAV-12, Werk Alliantie Vragenlijst. Notes: --, data not assessed or not applicable.*, (α) for domain *theory of change* was 0.82.**,This is a ranged α score. †, Omega Coefficient. “+”, sufficient; ”?”, Indeterminate; “-“, insufficient. #For more details regarding ratings see Table 1.

**Table S5.** Cross-cultural validity and measurement invariance of the Working Alliance Inventory and adapted versions.

| **Author ( publication year)** | **Country/ Language** | **Treatment, study population, (n) for analysis** | **Method** | **Results** | **Methodological quality** | **Rating#** |
| --- | --- | --- | --- | --- | --- | --- |
| Hatcher (2006)  WAI-SR-P | USA/ English | Sample 1. Psychotherapy; patients with depression, anxiety and relationship problems, (n=231) | Model fit of the invariance between the criterion sample (1) and the replication sample (2) was tested according to Byrne’s (2001) criteria | The variance of the Goal and Bond factors is allowed to be independent (unconstrained). For this combined model, χ2 (115, n*=* *466*)= 286.1, TLI=0.94, CFI=0.95, RMSEA=0.057, p = 0.09, indicating close fit | Doubtful | ? |
|  |  | Sample 2. Psychotherapy counseling; outpatients, (n=235) |  |  |  |  |
| Munder (2009)  WAI-SR-P | Germany/  German | Sample 1. Psychotherapy; inpatients with depression, eating, adjustment and personality disorders, (n=243) | Multigroup analysis comparing equivalence of the factor structure in outpatients and inpatients, according to the method of Hair et al., (2006) | The comparison of the unconstrained model and the model with constrained factor loadings showed that the WAI-SR measured the same constructs in both groups (p= 0.812) | Inadequate | + |
|  |  | Sample 2. Psychotherapy; outpatients with depression, anxiety, adjustment and personality disorders, (n=88) |  |  |  |  |
| Falkenström (2015)a  WAI-SR-P | USA/ English | Sample 1. Psychotherapy; outpatients; different treatments (mostly CBT or psychodynamics), (n=235) | Measurement invariance by means of analyses among the three samples was tested according to the method of Schoot et al., (2012) | For all comparisons, metric invariance held. As indicated by non-significant chi-square difference tests and ∆CFI smaller than 0.01. When the Swedish and English samples were compared, scalar invariance did not hold, by significant chi-square test and ∆CFI larger than 0.01 | Doubtful | - |
|  | Sweden/ Swedish | Sample 2. Primary care psychotherapy; different treatments (mostly CBT or psychodynamics), (n=634*)* |  |  |  |  |
|  | Sweden/ Swedish | Sample 3. Specialist psychiatric care; different treatments *(n=*234*)* |  |  |  |  |
| Falkenström (2015)b  SAI-P | Sweden/ Swedish | Primary care psychotherapy treatments (mostly CBT or psychodynamics), t1 (n=1061*) / t10 (*n=120*)* | Longitudinal measurement invariance | The longitudinal measurement invariance analyses for the first ten sessions indicated that strong measurement invariance generally held. Apart from session 1, factor loadings were stable except for a few minor deviations | Doubtful | ? |
| Hatcher (2020)  WAI-S-T  WAI-SR-T  WAI-S-T-IRT | USA/  English | Sample 1. Psychology; Therapists from 42 college counseling services treating outpatients; P (n=686) T (n=131) | Measurement invariance across therapists i.e., the test of “cluster bias” Jak, Oort, & Dolan (2013) | All ∆CFI models were smaller than -.01; therefore, the first test of the cluster was passed. Testing equality of intercepts across groups in multigroup analysis showed significant and large reductions in model fit according to both χ2 difference tests and CFI differences (ΔCFI WAI-SR-T = −0.151, WAI-S-T = −0.102, WAI-S-T-IRT = −0.135), indicating that all models failed the second test of cluster bias regarding item intercepts. | Doubtful | - |
|  |  | Sample 2-4 combined. Psychology, therapists from 3 different study samples; P(n=1117) T(n= 394) |  |  |  |  |
| Milot-Lapointe (2020)  WAI-S-P | Canada/  French | Career counseling; undergraduate or graduate students or workers from 11 organizations, clients with different career counseling goals; t1 first session (n=283) / t2 second session (n=217) | Longitudinal measurement invariance, tested according to Kim & Wilson (2014) | Between two sessions nonsignificant difference were found (χ2 = 18.18), a change in CFI (ΔCFI= 0.01) was lower than the criteria 0.01, and a change in RMSEA (ΔRMSEA= 0.002) lower than 0.015. Result showed that the WAI-S-P is invariant between the first and the third career counseling session. | Doubtful | ? |
| Cirasola (2021)  WAI-S-P | UK/English | Youth psychotherapy; adolescents with depression; t1 6 weeks (n=223), t2 12 weeks (n=247), 36 weeks (n=222) | Longitudinal measurement invariance, tested according to Cheung & Rensvold (2003) | Between three time points nonsignificant differences were found(χ2 = 22.09 p=0.335), a change in CFI (ΔCFI= 0.001) was lower than the criteria 0.01. Two-factor model consistently had the best fit to the data across time | Doubtful | ? |
| WAI-S-T |  | Youth psychotherapy; adolescents with depression; t1 6 weeks (n=139), t2 12 weeks (n=119), 36 weeks (n=63) |  | Between three time points nonsignificant difference were found (χ2 = 21.63 p=0.361), a change in CFI (ΔCFI= 0.001) was lower than the criteria 0.01. Two-factor model consistently had the best fit to the data across the time | Inadequate | ? |

Legends: CBT, Cognitive-Behavioral Therapy. CFI, Comparative fit index. n, number. RMSEA, root mean square error of approximation. SAI-P, Session Alliance Inventory Patient form. TLI, Tucker-Lewis index. WAI-IRT, Working Alliance Inventory Item Response Theory. WAI-S-T, Working Alliance Inventory Short Therapist Form. WAI-SR-P, Working Alliance Inventory Short Revised Patient Form. WAI-SR-T, Working Alliance Inventory Short Revised Therapist Form. “+”, sufficient; “?”, Indeterminate; “-“, insufficient. # For more details regarding ratings see Table 1.

**Table S6.** Test-retest reliability, inter- rater reliability and measurement error of the Working Alliance Inventory and adapted versions.

| **Author (publication year)/ WAI-Version** | **(n) for analysis/sessions ia/ (raters ia)** | **Measure / Model/ Calculation** | **Time interval** | **Results*** | **Methodological quality** | **Rating#** |
| --- | --- | --- | --- | --- | --- | --- |
| Tichenor (1989)/WAI-O | (4) sessions (6 raters) | ICC_(--)_ for inter-rater reliability /-- | -- | ICC 0.92 | Inadequate | + |
| Cerero (2001)/WAI-O | (60) sessions (6 raters) | Fixed & random-effects ICC_(--)_ for inter-rater reliability/- | -- | ICC 0.81 | Inadequate | + |
| Fenton (2001)/WAI-O | (8) sessions ( 6 raters) | Random-effect ICC_(--)_ for inter-rater reliability/-- | -- | ICC 0.70 | Inadequate | + |
| Goldberg (2004)/WAI-S-P | (23) | ICC_(--)_ for test-retest reliability/ -- | 2 weeks | ICC 0.78 | Inadequate | + |
| Goldberg (2004)/WAI-S-T | (18) sessions (4 raters) | ICC_(--)_ for test-retest reliability/ -- | 2 weeks | ICC 0.91 | Inadequate | + |
| Ely (2005)/WAICC versions | (15) child-versions\|  (10) adolescent versions | Correlation between t1 and t2 for test-retest reliability | 1 months | *r* range (-.19 to 0.99 ) | Inadequate | - |
| Soygüt (2009)/WAI-S-O | 10 (3 raters) | ICC_(--)_ for inter-rater reliability/ -- | -- | ICC 0.75 (95% CI 0.54;0.88) | Inadequate | + |
| Tatman (2010)/WAI-SR-P | 166 | Correlation for test-retest reliability/-- | 2 weeks | *r* 0.70 | Doubtful | ? |
| Hsu (2016)/ WAI-S-P | 123 | Correlation between t1 and t3 for test-retest reliability | 2 sessions | *r* 0.56 | Inadequate | - |
| Araujo (2017)/WAI-S-P | 89 | ICC_(2,1)_ for test-retest reliability/ SEM = sd √ 1-ICC/ MDC = 1.96 √ 2. SEM | 2 months | ICC 0.74 (95% CI 0.66;0.81)  SEM 3.30  MDC 9.14 | Inadequate Inadequate  Inadequate | +  ?  ? |
| Araujo (2017)/WAI-S-T | 18 | ICC_(2,1)_ for test-retest reliability/ SEM = sd √ 1-ICC/ MDC = 1.96 √ 2. SEM | 2 months | ICC 0.85 (95% CI 0.79;0.89)  SEM 3.20  MDC 8.87 | Inadequate Inadequate  Inadequate | +  ?  ? |
| Santirso (2018)/WAI-S-O | 140 (4 raters) | ICC_(2,4)_ for test-retest reliability | -- | ICC 0.82 | Adequate | + |
| Tabasaki (2018)/WAI-S-P | 101 | ICC_(--)_ for test-retest reliability/-- /MDC = 1.96 √ 2. SEM | ± 8 Days | ICC 0.84  MDC 2.10 | Doubtful  Doubtful | +  ? |
| Warlick (2019)/WAIT-3-P | 119 | ICC_(--)_ for test-retest reliability/ -- | -- | ICC 0.90 | Inadequate | + |
| Warlick (2019)/WAIT-12-P | 107 | ICC_(--)_ for test-retest reliability/ -- | -- | ICC 0.96 | Inadequate | + |

Legends: CI, confidence interval. ia, if applicable. ICC, Intraclass Correlation Coefficient. MDC, Minimal Detectable change. n, number of patients. O, observer form. P, patient form. sd, standard deviation. SEM, Standard error of the measurement. T, therapist form. WAICC, Working Alliance Inventory for Chronic Care (12 different versions). WAI, Working Alliance Inventory. WAI-S, Working Alliance Inventory Short Form. WAI-SR, Working Alliance Inventory Short Revised Form. WAIT, Working Alliance Inventory for Tobacco. *, rounded to 2 decimals. +, sufficient;?, Indeterminate; -, insufficient. (--), not specified. # For more details regarding ratings see Table 1.

**Table S7.** Construct validity of the Working Alliance Inventory and adapted versions.

| **Author (publication year)** | **Hypothesis** | **Results** | | | | | | | | | | | | | | | | | | | **Methodolo-gical quality** | **Rating#** |
| --- | --- | --- | --- | --- | --- | --- | --- | --- | --- | --- | --- | --- | --- | --- | --- | --- | --- | --- | --- | --- | --- | --- |
| Horvath (1989) | Study 1:  WAI correlates stronger with CPQ scales than Empathy scales (Attractiveness, Trustworthiness, Expertness, Empathy)  Study 2:  WAI and domains correlate moderately with CPQ scales  WAI task scale correlates with the indecision scale bears, STAI State scale and Target Complains  Study 3:  The WAI correlates with outcome variance measures of the CPQ and other outcome related variables | Convergent and divergent validity expressed in r | | | | | | | | | | | | | | | | | | | Doubtful | ? |
|  |  | Study 1  Task WAI  Bond WAI  Goal WAI  Total score WAI  Attractiveness  Trustworthiness  Expertness  Empathy | | Satisfaction  0.65 *  0.32 ns  0.40 *  0.50 *  0.07 ns  0.02 ns  0.15 ns  0.11 ns | | | | | | Client Change CPQ  0.45 *  0.23 ns  0.24 ns  0.33 *  -0.06 ns  -0.10 ns  0.09 ns  0.05 ns | | | | | | | | Adjustment CPQ  0.31 ns  0.21 ns  0.09 ns  0.22 ns  0.03 ns  0.16 ns  0.14 ns  0.26 ns | | |  |  |
|  |  | Study 2 a  Task WAI  Bond WAI  Goal WAI  Total score WAI  Study 2 b  Task WAI  Expertness  Trustworthiness  Attractiveness  Empathy | | Satisfaction  0.68 *  0.48 *  0.60 *  0.66 *  Indecision  0.68 **  0.28 ns  -0.17 ns  -0.08 ns  -0.45 ns | | | | | | Client Change CPQ  0.37 *  0.47 *  0.22 ns  0.38 *  STAI  0.55 **  -0.08 ns  0.11 ns  -0.08 ns  -0.29 ns | | | | | | | | Adjustment CPQ  0.32 ns  0.16 ns  0.25 ns  0.27 ns  Target Complaints  0.65 **  0.26 ns  0.19 ns  -0.02 ns  -0.34 ns | | |  |  |
|  |  | State Anxiety  Self-Concept  Target Complaint  Satisfaction CPQ  Client Change CPQ  Adjustment CPQ  Total score CPQ | | Bond  -0.16 ns  0.11 *  -0.51 *  0.71**  0.25 ns  0.21 ns  0.46 * | | | | | | Task  -0.13 ns  0.21*  0.53 **  0.63 **  0.30 ns  0.35 ns  0.50 ** | | | | | | | | Goal  -0.05 ns  0.02 ns  0.33 ns  0.50 **  0.16 ns  0.32 ns  0.37 * | | |  |  |
| Tichenor (1989) | WAI versions correlate strongly (*r* = 0.70) with CALPAS, Penn and VTAS  It was expected that CALPAS, Penn, WAI-O, and VTAS relied on judgment by observers, whereas the WAI-T and WAI-P were rated by therapists and patients | Convergent validity expressed in r | | | | | | | | | | | | | | | | | | | Inadequate | ? |
|  |  | CALPAS  Penn  VTAS WAI-O  WAI-C | | WAI-O  0.82 **  0.71 *  0.84 **  -- | | | | | | WAI-P  -0.33 ns  0.02 ns  0.13 ns  -0.18 ns  -- | | | | | | | | WAI-T  -0.22 ns  0.20 ns  0.09 ns  0.03 ns  -0.09 ns | | |  |  |
|  |  | Discriminative or known-groups validity  Comparison (One-Way Anova) between different measures: CALPAS, F(1,7)= 17.21, P<0.01; Penn, F(1,7)= 3.33, P<0.01; VTAS, F(1,7)=3.48, P<0.01; WAI-O, F(1.7) 8.77, P<0.01; WAI-P, F(1,7)=9.53, P<0.01; WAI-T, F(1,7)= 4.87, P<0.01 | | | | | | | | | | | | | | | | | | |  |  |
| Hatcher (1995) | No hypothesis | Convergent validity expressed in r | | | | | | | | | | | | | | | | | | | Inadequate | ? |
|  |  | CALPAS  HAq-II  WAI-T | | | WAI-P  0.83 ***  0.85 ***  0.29 *** | | | | | WAI-T  0.86 ***  0.76 *** | | | | | | | | | | |  |  |
| Hatcher (1996) | No hypothesis | Convergent validity expressed in r | | | | | | | | | | | | | | | | | | | Inadequate | ? |
|  |  | HAq-II  CALPAS | | | WAI- P  0.74 ***  0.85 *** | | | | | | | | | | | | | | | |  |  |
| Hatcher (1999) | It was expected to find an association between the WAI-T and therapist ratings of progress in treatment | Convergent validity expressed in r | | | | | | | | | | | | | | | | | | | Doubtful | ? |
|  |  | WAI-T | | | CALPAS  0.75 | | | | | | TUI  0.63 | | | | | | | PC  0.72 | | EI  0.54*** |  |  |
| Fenton (2001) | No hypothesis | Convergent validity expressed in r | | | | | | | | | | | | | | | | | | | Inadequate | ? |
|  |  | Outcome assessment | | | WAI-P  0.03 ns | | | | | WAI-T  r=0.27 ns | | | | | | | | WAI-O  0.39 *** | | |  |  |
| Stiles (2002) | 1. Strong associations exist between Bond scales from WAI and ARM 2. Strong associations exist between Task scales from WAI and ARM 3. Strong associations exist between WAI Bond, Task, Goal scales and ARM Bond, Partnership, Confidence scales 4. Weak associations exist between ARM Openness and Initiative domains and the WAI | Convergent validity expressed in r  90 correlations between the WAI patient, therapist and observer form and ARM domains  Hypotheses 1 confirmed  Hypotheses 2 rejected  Hypotheses 3 confirmed  Hypotheses 4 confirmed | | | | | | | | | | | | | | | | | | | Doubtful | + |
| Busseri (2003) | WAI-P and WAI-T scores have a similar predictive value as WAI-S-P and WAI-S-T, respectively | Convergent validity expressed in r | | | | | | | | | | | | | | | | | | | Inadequate | ? |
|  |  | WAI-P  WAI-S-P  WAI-T  WAI-S-T | | | Improvement  0.36 **  0.34 **  0.40 **  0.42 ** | | | | | Symptoms  0.14 ns  0.15 ns  0.15 ns  0.19 ** | | | | | | | | | | |  |  |
| Cerero (2001) | Strong correlations exist between different measures and observer-rated measures, but correlations between different perspectives (observer, patient, and therapist) are weaker | Convergent validity expressed in r  In total, 274 correlations between WAI-O, WAI-P, WAI-T, CALPAS, Penn, VTAS, were compared and it was concluded that the measures were strongly correlated. A pattern of strong positive correlations existed between the four observer-rated measures and the therapist versions of the WAI. Correlations between observer-ratings and participant ratings were low | | | | | | | | | | | | | | | | | | | Inadequate | ? |
| Guédeney (2005) | No hypothesis | Convergent validity  WAI- outcome assessment (GHQ) after 4 month (r=0.48,p=0.001)  Divergent validity by comparison between subgroups  Mean scores were significantly lower (p=0.03) between domains of the WAI and no indication of relational ruptures except for those patients with potential relational ruptures | | | | | | | | | | | | | | | | | | | Inadequate | ? |
| Bedregal (2006) | Correlation between TAC and TCS is strong | Convergent validity expressed in r | | | | | | | | | | | | | | | | | | | Inadequate | + |
|  |  | TCS | | | TAC  0.71 * | | | | | | | | | | | | | | | |  |  |
| Hatcher (2006) | No hypothesis | Convergent validity expressed in r | | | | | | | | | | | | | | | | | | | Inadequate | ? |
|  |  | HAq-II  CALPAS Total  EI filled in by P  EI filled in by T | | | Bond  0.59 nr  0.65 nr  0.40 ***  0.14 * | | | | Task  0.74 nr  0.79 nr  0.67 ***  0.25 *** | | | | | | Goal  0.57 nr  0.63 nr  0.40 ***  0.07 ns | | | | Total  0.74 nr  0.80 nr  0.56 ***  0.17 * | |  |  |
| Soygüt (2008) | No hypothesis | Convergent validity expressed in r | | | | | | | | | | | | | | | | | | | Inadequate | ? |
|  |  | Rationality of the service  Success in problem-solving  Success in decreasing problems  Recommending another person  Satisfaction with service on that day | | | | | | | | Bond  0.28 *  0.42 *  0.37 *  0.28 *  0.48 * | | | | Task  0.12 ns  0.41 *  0.30 *  0.30 *  0.16 ns | | | | | Goal  0.50 *  0.52 *  0.18 ns  0.44 *  0.21 * | |  |  |
| Wilmers (2008) | A higher correlation exists between WAI-SR scores and the satisfaction domain of the HAq-II than with the total score of the HAq-II | Convergent validity and divergent validity expressed in r | | | | | | | | | | | | | | | | | | | Doubtful | ? |
|  |  | HAq-II total  HAq-II satisfaction  Inpatients score  Out-patients score | | | Bond  0.56 ***  0.65 ***  0.72 ***  0.57 *** | | | | Task  0.70 ***  0.64 ***  0.90 ***  0.85 *** | | | | | | Goal  0.63 ***  0.69 ***  0.79 ***  0.61 *** | | | | Total  0.71 ***  0.75 *** | |  |  |
| Stinckes (2009) | No hypothesis | Divergent validity tested with MANOVAs  No effects were found for patient and therapeutic characteristics such as age, gender, education level and therapeutic orientation on WAI (domain) scores  Significantly higher task domain scores (p<0.05) were found for therapists with fewer years of experience | | | | | | | | | | | | | | | | | | | Inadequate | ? |
|  |  | Convergent validity | | | | | | | | | | | | | | | | | | |  |  |
|  |  | Rating of change  Depression (BSI) | | | Bond  0.36 **  -- | | | | Task  0.56 **  -0.13 ** | | | | | | Goal  0.47 **  -- | | | | Total  0.51 **  -0.13** | |  |  |
| Munder (2009) | No hypothesis | Convergent validity expressed in r | | | | | | | | | | | | | | | | | | | Inadequate | ? |
|  |  | Relationship (HAq-II)  Outcome (HAq-II)  Total (HAq-II) | | | Bond  0.65 ***  0.26 ***  0.56 *** | | | | Task  0.64 ***  0.55 ***  0.70 *** | | | | | | Goal  0.69 ***  0.36 ***  0.63 *** | | | | Total WAI-SR  0.75 ***  0.44 ***  0.71 *** | |  |  |
| Tatman (2010) | WAI-SR-P scores correlate with offender risk (LSI-R) scores | Divergent validity expressed in r | | | | | | | | | | | | | | | | | | | Doubtful | - |
|  |  | LSI-R | | | WAI-SR-P  -0.15 ns | | | | | WAI-SR-P retest scores  -0.19 * | | | | | | | | | | |  |  |
| Vöhringer (2013) | No hypothesis | Convergent validity expressed in r | | | | | | | | | | | | | | | | | | | Inadequate | ? |
|  |  | OQ45 total  VTAS-R | | | WAI-P  -0.53 ***  0.57 ns | | | | | WAI-T  -0.44 **  0.54 ns | | | | | WAI-O  -0.10 ns  0.59 ns | | | | | |  |  |
| Andrade-González (2015) | No hypothesis | Convergent validity expressed in r  Correlations between WAI-P and WAI-T on the one hand and HAq-II-P and HAq-II-T on the other were ≥ 0.73*  Divergent validity expressed in r  WAI-P - WAI-T did not correlate with the majority of demographic variables. Correlations between WAI-P and WAI-T on the one hand and EUS-P were ≥ .62*  Predictive validity expressed in *r* and tested with regression  Correlations between WAI-P and WAI-T on the one hand and BDI residual gain scores on the other hand were ≤ -.37*. WAI-P predicted patient change in the BDI [F change (1.28) = 5.26, R^2^ Change = .16, p = 0.03]. WAI-T predicted patient change in the BDI [F change (1.28) = 7.98, R^2^ Change = .22, p = 0.01] | | | | | | | | | | | | | | | | | | | Inadequate | ? |
| Falkenström (2015)b | Replication of previous findings | Divergent validity  SAI-P scores (session-by-session 1 to 10) predicted symptom reduction (CORE-OM) and analyses of an Autoregressive Latent Trajectory model showed that previous findings could be replicated | | | | | | | | | | | | | | | | | | | Doubtful | + |
| Lamers (2015) | Correlations between subscales of the WAV-12R and FEQ and EUQ are moderate to strong (≥ 0.3 = moderate ≥0.50 strong) | Convergent validity expressed in r | | | | | | | | | | | | | | | | | | | Doubtful | + |
|  |  | Team version  FEQ  Parent version  EUQ | | | Bond  0.57 **  0.50 ** | | | | Task  0.48 **  0.78 ** | | | | | | Goal  0.53 **  0.54 ** | | | | Total  0.56 **  0.75 ** | |  |  |
| Miragalll (2015) | No hypothesis | Convergent validity expressed in r | | | | | | | | | | | | | | | | | | | Inadequate | ? |
|  |  | WAI-VAR | | | WAI-S total  0.70 *** | | | | | | | | | | | | | | | |  |  |
|  |  | Divergent validity by comparison between subgroups  WAI-VAR –outcome assessment (total score r_s_ = 0.55 ***)  Patients who had not changed scored lower on the WAI-VAR compared with improved and recovered patients F(2,72)= 17.25, p ¸0.001, η² = 0.32 | | | | | | | | | | | | | | | | | | |  |  |
| Smits (2015) | Correlations are negative between alliance and symptomatic distress and interpersonal functioning measured with the OQ45  Correlations are positive between alliance and Extraversion, Agreeableness and Conscientiousness and are negative between alliance and Neuroticism measured with DAPP-SF | Divergent validity expressed in r | | | | | | | | | | | | | | | | | | | Doubtful | - |
|  |  | OQ45  Symptomatic Distress  Interpersonal functioning  DAPP-SF  Neuroticism  Agreeableness  Extraversion  Conscientiousness | | | | WAI-S-Contract (Task - Goal)  -0.15 **  -0.19 **  -0.18 **  -0.13 **  -0.07 **  -0.04 ** | | | | | | | | | | WAI-S-Contact (Bond)  -0.09 ns  -0.08 ns  -0.08 ns  -0.08 ns  -0.06 ns  0.03 ns | | | | |  |  |
| Toste (2015) | Correlations are positive between alliance and students’ self-perceptions in the academic, social, and behavioral domains of the Self-Perception Profile For Children | Convergent validity expressed in r | | | | | | | | | | | | | | | | | | | Doubtful | ? |
|  |  | CWAI-S  Bond  Task/Goal  CWAI-T  Bond  Task/Goal | Scholastic Competence  0.12 ns  0.14*  0.10 ns  0.125ns | | | | | | | | | Social Acceptance  0.09 ns  0.13*  0.14 ns  0.10 ns | | | | | | Behavioral Conduct  0.22*  0.21**  0.22**  0.21 ns | | |  |  |
| Andrade-González (2016) | No hypothesis | Convergent validity expressed in r  Correlations between WAI-S-P and WAI-S-T on the one hand and HAq-II-P - HAq-II-T on the other were all ≥0.74**  Divergent validity expressed in r  WAI-S-P and WAI-S-T did not correlate significantly with demographic variables  Correlations between WAI-S-P - WAI-S-T on the one hand and EUS-P all ≥ 0.57**)  Predictive validity expressed in r  Correlations between WAI-S-P - WAI-S-T on the one hand and BDI residual gain scores on the other were -0.37* and -0.41*  Results of a stepwise regression analysis WAI-S-T explained 17% of the variance in patient change in the BDI residual gain. Other domains and WAI-S-P were excluded in the model | | | | | | | | | | | | | | | | | | | Inadequate | ? |
| Figueiredo (2016) | No hypothesis | Convergent validity expressed in r | | | | | | | | | | | | | | | | | | | Inadequate | ? |
|  |  | Child/ adolescent  WAI-P Goal  WAI-P Task^1^  WAI-P Bond^1^  WAI-P Total^1^  Parent  WAI-P Goal  WAI-P Task  WAI-P Bond  WAI-P Total | | WAI-CA Goal  0.83 ***  0.63 ***  0.55 ***  0.76 ***  0.29 *  0.32 *  0.22 ns  0.30 * | | | | | WAI-CA Task  0.58 ***  0.66 ***  0.63 ***  0.65 ***  0.31 *  0.29 *  0.26 ns  0.31 * | | | | | | WAI-CA Bond  0.77 ***  0.76 ***  0.86 ***  0.84 ***  0.16 ns  0.17 ns  0.30 *  0.21 ns | | | | WAI-CA Total  0.82 ***  0.77 ***  0.76 ***  0.86 ***  0.28 *  0.29 *  0.29 *  0.31 * | |  |  |
| Mallinckrodt (2016) | No hypothesis | Convergent and divergent validity expressed in r | | | | | | | | | | | | | | | | | | | Inadequate | ? |
|  |  | WAI-SR Bond  WAI-SR Task  WAI-SR Goal  WAI-SR Total  CATS secure  CATS Avoidant  SEQ Depth  SEQ Smoothness  OQ45 | | | BAI Bonds  0.96 **  0.69 **  0.73 **  0.89 **  0.77 **  -0.66 **  0.50 **  0.32 **  -0.28 ** | | | | | BAI Task/Goal  0.68 **  0.82 **  0.86 **  0.89 **  0.75 **  -0.58 **  0.59 **  0.35 **  -0.29 ** | | | | | | BAI Total  0.88 **  0.80 **  0.84 **  0.95 **  0.81 **  -0.67 **  0.60 **  0.37 **  -0.30 ** | | | | |  |  |
| Araujo (2017) | No hypothesis | Convergent validity expressed in r | | | | | | | | | | | | | | | | | | | Inadequate | ? |
|  |  | SRS  WAI-S-T | | | WAI-S-P  0.39 ***  0.09 ns | | | | | | | | | | | | | | | |  |  |
| Killian (2017) | WAI-S , and all three versions (P-T-O) correlated well with the Yatchmenoff total and subscales  The therapeutic alliance is not associated with factors surrounding the family such as alcohol problems, depression, illicit substance use, domestic violence, and possible learning disabilities | Convergent validity expressed in r | | | | | | | | | | | | | | | | | | | Doubtful | ? |
|  |  | WAI-S-P  WAI-S-T  WAI-S-O | Working Relationship  0.82 ***  0.31 ***  0.57 *** | | | | | | | | | | Mistrust  -0.82 ***  -0.34 ***  -0.47 *** | | | Total Yatchmenoff Scale  0.77 ***  0.41 ***  0.56 *** | | | | |  |  |
|  |  | Discriminant and known-group validity  No associations were found between versions of the WAI-S and alcohol problems, depression, illicit substance use, domestic violence, or possible learning disabilities  Social workers reported lower total scores on the WAI-S for those families having suspected problems with depression, F(2.262)=7.285, P=0.001, η²=0.05; however, differences were not found for the other versions of the WAI-S for these families | | | | | | | | | | | | | | | | | | |  |  |
| Bat (2018) | No hypothesis | Convergent validity expressed in r | | | | | | WAI-P | | | | | | | | | | | | | Inadequate | ? |
|  |  | AT-WAI-P  Task  Experience  Acceptance | | | Bond  0.52 **  0.35 **  0.40 ** | | | | | Task  0.56 **  0.18 ns  0.25 ** | | | | | | Goal  0.43 **  0.18 ns  0.32 ** | | | | |  |  |
| Chen (2018) | No hypothesis | Convergent validity expressed in r | | | | | | | | | | | | | | | | | | | Inadequate | ? |
|  |  | PDRQ-15  WFPTS | | | Bond  0.71 ***  0.50 *** | | | | | Task  0.54 ***  0.32 ** | | | | | | Goal  0.73 ***  0.53 *** | | | | |  |  |
| Paap (2018) | Correlation of the total WAI-ReD score with HAq-II is (*r*≥0.70) (1)  Correlation of the total WAI-ReD score with total score SRS is (*r*≥0.70) (2)  Correlation of the WAI-ReD domains with SRS domains is (0.50*≤r*≤0.70) (3)  Correlation of the total WAI-ReD score with VAS_pain_ is (0.00*≤r*≤ 0.30) (4)  Differences in WAI-ReD total scores between males and females are not significant (5)  Difference in WAI-ReD total scores between two age groups (below and above mean age) are not significant (6)  Differences in WAI-ReD total scores between different types of treating therapists are not significant (7) | Convergent validity expressed in r | | | | | | | | | | | | | | | | | | | Adequate | ? |
|  |  | SRS  Approach  Goal and topics  Relationship  SRS Total  HAq-II  VAS-Pain | | Bond  0.52**  0.55 **  0.52 **  --  --  -- | | | | | Task  r 0.52 **  r 0.51 **  r 0.52 **  --  --  -- | | | | | | Goal  r 0.59 **  r 0.58 **  r 0.55 **  --  --  -- | | | | Total  --  ---  --  0.69 **  0.74 **  -0.23 * | |  |  |
|  |  | Discriminant and known-group validity  Difference between males and females in WAI-ReD total scores were not significant (P=0.243)  Differences between the two ages groups in WAI-ReD total scores were not significant (P=0.118)  The mean in WAI-ReD total scores was significantly different between the type of treating therapist (F4,125=9.48, P=0.001) | | | | | | | | | | | | | | | | | | |  |  |
| Santirso (2018) | No hypothesis | Convergent validity expressed in r | | | | | | | | | | | | | | | | | | | Inadequate | ? |
|  |  | Pro-therapeutic group behavior  Stage of change  Motivation of change | | Goal/Task WAI-S-  0.69**  0.29 **  0.33 ** | | | | | | Bond WAI-S-O  0.73 **  0.17 *  0.22 * | | | | | | | | Total WAI-S-O  0.73**  0.25 *  0.29 ** | | |  |  |
| Sturgiss (2018) | The patient measure of WAI-GP is strongly associated with Dyadic OPTION (measuring shared decision perceptions) and with the Patient-Doctor Depth of Relation scale and not correlated with the measures of social desirability | Convergent and divergent validity expressed in r | | | | | | | | | | | | | | | | | | | Inadequate | + |
|  |  | Patient-Doctor Depth of Relation scale  Dyadic OPTION  Crowne-Marlow Social Desirability scale  Haghhighat Brief Social Desirability Scale | | | | | | | | Total score WAI-GP  0.591 ***  0.705 ***  0.105 ns  0.009 ns | | | | | | | | | | |  |  |
| Paap (2019) | The strength of the correlations between WAI-ReD total scores and the SRS and HAq-II scores is ≥ 0.60 | Convergent validity expressed in r | | | | | | | | | | | | | | | | | | | Very good | + |
|  |  | SRS  HAq-II | | | Total WAI-ReD  0.85 **  0.75 ** | | | | | | | | | | | | | | | |  |  |
| Penedo (2019) | Correlations above 0.85 indicate convergent validity. Correlations below 0.50 indicate evidence for discriminant validity | Convergent validity expressed in r | | | | | | | | | | | | | | | | | | | Very good | ? |
|  |  | ZUF-8  APOI Total | | | Bond WAI-I  0.54 ***  0.26 *** | | | | | Goal/Task WAI-I  0.82 ***  0.27 *** | | | | | | Total WAI-I  0.75 ***  0.29 *** | | | | |  |  |
| Warlick (2019) | WAIT-3 and WAIT-12 are significantly correlated with post-counseling attempts to quit smoking. | Discriminative or known-groups validity tested with logistical regression  WAIT-3 was significantly associated with post-counseling attempts to quit smoking; B=1.01,Exp(B)= 2.75, 95% CI= 1.75- 4.31, R^2^range= 0.18- 0.27, p < 0.001 in sample 1. Results were replicated in sample 2 (odds 3.71)  WAIT-12 was significantly associated with post-counseling attempts to quit smoking; B=1.23,Exp(B)= 3.43, 95% CI= 1.93- 6.08, R^2^range= 0.20- 0.28, p < 0.001 in sample 2  Further analysis showed a greater working alliance is associated with a higher increase in the odds of a 7-day point-prevalence smoking abstinence (odds 2.59 in sample 1 and 2.50 in sample 2), and less cigarettes per day post-interventions (odds 0.9 in sample 1, and 0.17 in sample 2) | | | | | | | | | | | | | | | | | | | Doubtful | + |
| Herrero (2020) | Higher WAI-SR-P-TECH scores predict therapeutic outcomes (i.e., change in depressive symptoms scores) and satisfaction with the treatment | Predictive and known-groups validity tested with logistical regression | | | | | | | | | | | | | | | | | | | Doubtful | + |
|  |  | Regression analyses showed that the WAI-SR-P-TECH predicted changes in depression symptoms significantly F(1.118)= 14.42, p < 0.001, R^2^= 6.7%  Also satisfaction with the treatment was significant in the model F(1.187)= 185.53, p < 0.001, R^2^= 49.7% | | | | | | | | | | | | | | | | | | |  |  |
| Hunik (2020) | It was hypothesized that the WAI-S-P-GP scores would correlate with the CARE and PPPC scores | Convergent validity expressed in *ρ* | | | | | | | | | | | | | | | | | | | Inadequate | + |
|  |  | WAI-S-P-GP | | | | | CARE  0.56** | | | | | | | | | | PPPC  -0.51** | | | |  |  |
| Miloff (2020) | It was hypothesized that the VTAS-P scores would correlate significantly with treatment outcomes and offer insights into what components of the virtual therapist are most important for treatment efficacy | Convergent validity expressed in r | | | | | | | | | | | | | | | | | | | Doubtful | ? |
|  |  | System Usability Scale  Fear of Spiders Questionnaire  Pre-post  Post-follow-up | | | | | | | | | VTAS-P  0.351**  -0.21*  -0.31* | | | | | | | | | |  |  |
|  |  | Predictive and known-groups validity tested with multiple regression  Of the four covariates, user friendliness, presence, and follow-up, Fear of Spiders Questionnaire changes scores were all significantly associated with the VTAS-P | | | | | | | | | | | | | | | | | | |  |  |

Legends: APOI, Attitudes towards Psychological Online- Interventions Questionnaire. ARM, Agnew Relationship Measure. AT-WAI, Art Therapy- Working Alliance Inventory. BAI, 16-items Brief Alliance Inventory. BDI, Beck Depression Inventory. BSI, Brief symptom index. CALPAS, California Psychotherapy Alliance Scale. CARE, Consultation and Relational Empathy. CATS, The Client Attachment to Therapist. CORE-OM, Clinical Outcomes in Routine Evaluation. CPQ, Client Posttherapy Questionnaire. CWAI, Classroom Working Alliance Inventory. DAPP-SF, Dimensional Assessment of Personality Pathology- Short form. EI, Estimate of improvement. EUS-P, Empathic Understanding Scale of the Relationship Inventory Patient Version. EUQ, Empathy and Understanding Questionnaire. FEQ, Family Engagement Questionnaire. GHQ, General Health Questionnaire 28. HAq-II, Helping Alliance Questionnaire-II. LSI-R, Level of Service Inventory-Revised. O, Observer form. OQ, Outcome Questionnaire. OQ45, Outcome Questionnaire 45. P, Patient form. PDRQ-15, Patient-Doctor Relationship Questionnaire-15. PC, Patient confidence and Commitment. Penn, Pennsylvania Helping Alliance Rating Scale. PPPC, Patient Perception of Patient-Centeredness. SAI-P, Session Alliance Inventory Patient form. SEQ, The Session Evaluation Questionnaire. SRS, Session Rating Scale. STAI, State- Trait Anxiety Inventory. T, Therapist form. TAC, Therapeutic Alliance with Clinician. TUI, Therapist understanding and involvement. VAS, Visual Analog Scale. TCS, Therapeutic Collaboration Scale. VTAS, Vanderbilt Therapeutic Alliance Scale Revised version. VTAS-R, Vanderbilt Therapeutic Alliance Scale Revised version. VTAS-P, Virtual Therapist Alliance Scale patient version. WAI, Working Alliance Inventory. WAI-CA, Working Alliance Inventory for Children and Adolescents. WAI-GP, Working alliance Inventory for General Practice. WAIT, Working Alliance Inventory for Internet Interventions. WAI-S, Working Alliance Inventory Short form. WAI-SR, Working Alliance Inventory short revised form. WAI-SR-TECH, Working Alliance Inventory Short Form Revised for online Interventions. WAI-ReD, Working Alliance Inventory Rehabilitation Dutch Version. WAI-VAR, Working Alliance Inventory applied to virtual and augmented reality. WAIT, Working Alliance Inventory for Tobacco. WAV-12R, Working Alliance Inventory Dutch Version short form (revision). WFPTS, Wake Forest Physician Trust Scale. ZUF-8, Patient satisfaction Questionnaire. r, Pearson Correlation Coefficient.r_s,_ Spearman's Rank Correlation Coefficient. ns: not significant, *p≤0.05,**p≤ 0.01, ***p≤ 0.001. nr, significance not reported. “+”, sufficient; “?”, Indeterminate; “-“, insufficient. # For more details regarding ratings see Table 1.

**Table S8.** Responsiveness of the Working Alliance Inventory and adapted versions.

| **Author ( publication year)** | **Hypothesis / type of approach** | **Results** | **Methodological quality** | **Rating#** |
| --- | --- | --- | --- | --- |
| Araujo (2017) | No hypothesis formulated / Before and after intervention (ES method) | WAI-S-P (ES = 0.15; 84% CI: 0.04 to 0.29)  WAI-S-T (ES = 0.37; 84% CI:-0.29 to 0.49) | Inadequate | - |

Legends: WAI-S- P, Working Alliance Inventory Short Patient Form. WAI-S-T, Working Alliance Inventory Short Therapist form. ES, Effect Size. CI, Confidence Interval. “-“, insufficient. # For more details regarding rating see Table 1.
